# Once a week consumption of Western diet over twelve weeks promotes sustained insulin resistance and non-alcoholic fat liver disease in C57BL/6 J mice

**DOI:** 10.1038/s41598-023-30254-2

**Published:** 2023-02-21

**Authors:** Thainá Magalhães Demaria, Leticia Diniz Crepaldi, Emylle Costa-Bartuli, Jessica Ristow Branco, Patricia Zancan, Mauro Sola-Penna

**Affiliations:** The Metabolizsm’ Group, Departamento de Biotecnologia Farmacêutica, Faculdade de Farmácia, Universidade Federal Do Rio de Janeiro, Rio de Janeiro, RJ 21941-902 Brazil

**Keywords:** Metabolism, Diabetes, Metabolic syndrome

## Abstract

The Western diet (high in fat and sucrose) consumption is a highly prevalent feature in the whole world, mainly due to the increasing consumption of ultra-processed foods (UPF), which are cheaper and easier-to-eat, as compared to fresh and highly nutritive meals. Epidemiological studies have associated UPF consumption with development of obesity, non-alcoholic fat liver disease (NAFLD) and insulin resistance. For molecular studies, mice fed with Western diets have been used to characterize signaling pathways involved in these diet-induced pathologies. However, these studies fed mice continuously with the diets, which is not compatible with what occurs in real life, when consumption is occasional. Here, we fed mice once-a-week with a high fat, high sucrose (HFHS) diet and compared these animals with those fed continuously with HFHS diet or with a standard diet. Our results show that after a single day of consuming HFHS, animals presented impaired oral glucose tolerance test (oGTT) as compared to control group. Although this impairment was reversed after 24 h consuming regular diet, repetition of HFHS consumption once-a-week aggravated the picture such as after 12-weeks, oGTT impairment was not reversed after 6 days under control diet. Liver steatosis, inflammation, impaired insulin signaling pathway and endoplasmic reticulum stress are similar comparing animals that consumed HFHS once-a-week with those that continuously consumed HFHS, though weekly-fed animals did not gain as much weight. Therefore, we conclude that regimen of one day HFHS plus 6 days normal diet over 12 weeks is sufficient to induce insulin resistance and NAFLD in mice.

## Introduction

The Metabolic Syndrome (MS), which comprises obesity, Type 2 Diabetes Mellitus (T2DM), cardiovascular diseases (CVD), non-alcoholic fat liver disease (NAFLD) and non-alcoholic steatohepatitis (NASH), has reached pandemic proportion more than fifty-years ago and continue dramatically increasing worldwide^1–3^. This pandemics evolution directly correlates to the dietary pattern change of the population that augmented the consumption of processed and ultra-processed foods (UPF), rich in simple sugars and saturated fats^4^. This type of food characterizes the so-called Western diets, named after its origin in Western industrialized countries, although currently highly consumed globally, specially by low-income population due to its attractive price as compared to highly nutritive fresh nourishments^1–4^. Indeed, epidemiologic studies link the consumption of Western diets to augmented risk of developing different conditions, such as obesity^5,6^, T2DM^2,7^, CVD^8,9^, liver diseases^2^, cancers^1,3^, Alzheimer’s disease^10^ among others^4^.

Animal models have been frequently used to evaluate the effects of Western diets on development of obesity and its comorbidities^11–14^. For these studies, animals were subjected to a continuous long-term diet enriched in saturated fat and simple sugars. One feebleness of these studies, as compared to human Western diet ingesting regimen, is that, normally, the regular consumption is not continuous, but intercalated with different kinds of nourishments^15^. Nevertheless, different studies have reported that acute consumption of different high-fat and/or high-sucrose diets triggers similar responses to those observed in long-term ingestion^16–21^. Considering this, we raised two main questions concerning acute ingestion of Western diets: first, are the effects reversible?; and second, what would be the effects simulating the sporadic, but long-term consumption of this diet, like people that eat fast-food on Sundays, for instance?

In the current work, we fed C57BL/6 J mice a high-fat, high-sucrose (HFHS) diet once a week during twelve consecutive weeks and compared to animals consuming regular Chow diet or a continuous HFHS diet. The results show that after this period, the occasional consumption of the HFHS diet presents similar effects to continuous consumption regarding insulin resistance and liver inflammation.

## Results

**Figure 1:**
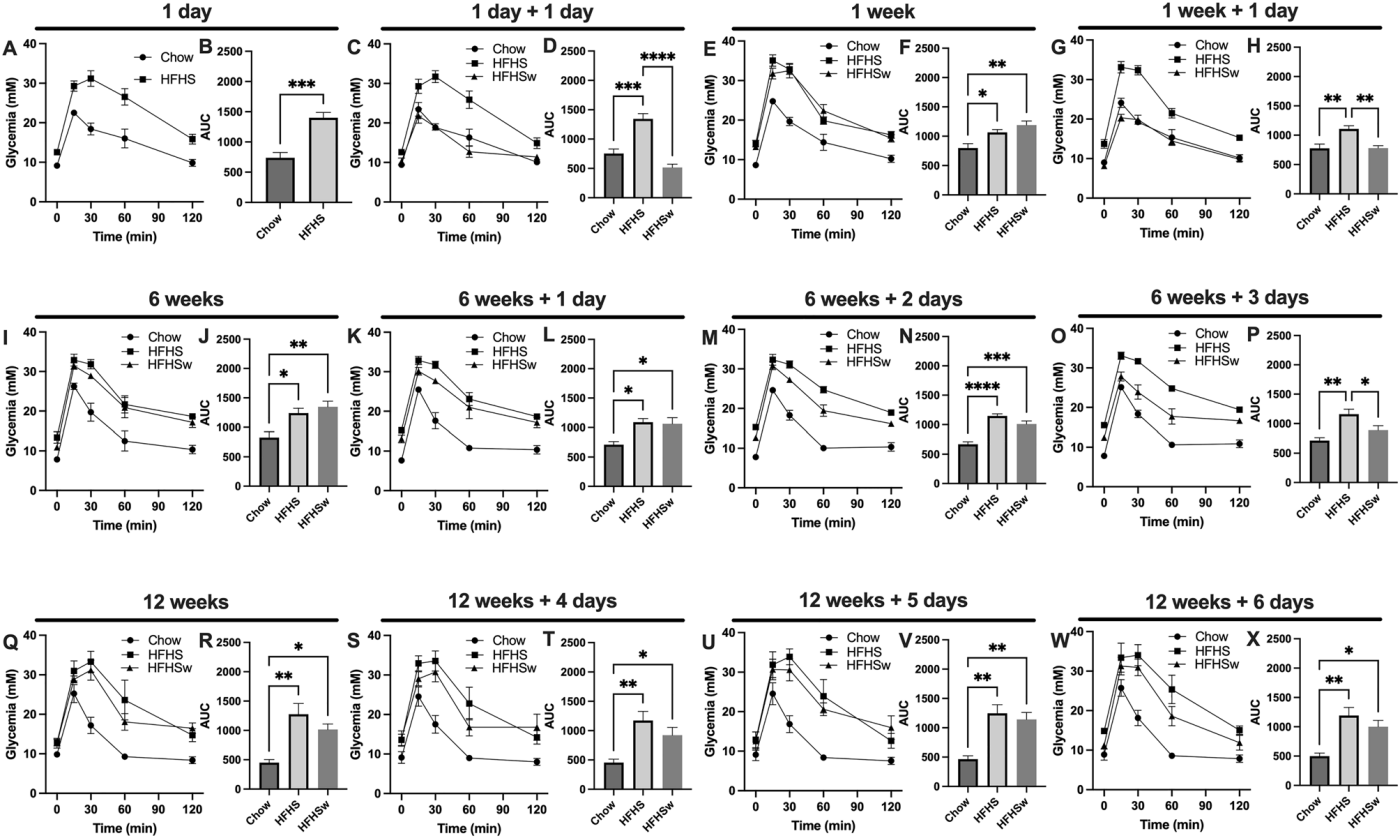
Once-a-week consumption of HFHS diet impairs oral glucose tolerance test in C57BL/6 J mice. Panels were paired as the oGTT curves and the area under the curves for the three groups, Chow, HFHS and HFHSw, except Panels A and B were there was no HFHSw group. Each test was performed with 6 animals (n = 6) and plotted values are mean ± S.E.M. *P < 0.05; **P < 0.01; ***P < 0.001; ****P < 0.0001. For Panel B, a Student’s t-test was applied. For the other panels reporting A.U.C, One-way ANOVA followed by Tukey’s post-test was performed.

Aiming to simulate low-frequency Western diet consumption, C57BL/6 J mice were subjected to the HFHS diet once a week for 12 continuous weeks., *i.e.,* the animals were fed an HFHS diet for 24 h followed by 6 days (144 h) receiving Chow diet for 12 cycles. This group of animals, fed an HFHS diet once a week, will be hereon referred to as HFHSw. As a matter of comparison, a group fed continuously with the chow diet and a group fed continuously with the HFHS diet will be used and referred to as Chow and as HFHS, respectively. When first challenged with the HFHS diet for 24 h, the mice presented an impaired oGTT as compared to the control Chow group (Fig. 1A,B). When these animals were refed with a chow diet for the next 24 h (HFHSw group), the oGTT profile were not different from the control Chow group (Fig. 1C,D). It is important to note that the oGTT profile of the HFHS mice remains impaired as compared to Chow and HFHSw groups (Fig. 1C,D). After 6 days receiving the chow diet, HFHSw mice were fed with the HFHS diet for 24 h, which again promoted the impairment of their oGTT as compared to Chow mice (Fig. 1E,F). Curiously, after this second challenge with the HFHS diet for 24 h, the oGTT from HFHSw mice was as impaired as those from mice that continuously received the HFHS diet (Fig. 1E,F). Again, when HFHSw mice were refed with the chow diet, the oGTT profile has recovered to a pattern like the Chow group and different from the HFHS group (Fig. 1G,H). The oGTT tests were repeated after 6 weeks of treatment and again the impairment observed for the HFHSw mice was like those observed for the HFHS group (Fig. 1I,J). However, after being fed with the chow diet, the HFHSw mice did not recover glucose tolerance either after 24 h (Fig. 1K,L) or 48 h (Fig. 1M,N). Indeed, only 3 days after being refed with the chow diet the animals from the HFHSw group responded to oGTT like the Chow group (Fig. 1O,P). After 12 weeks of treatment, animals that received the HFHS diet once a week (HFHSw group) were as intolerant to glucose as the animals that had continuously consumed the HFHS diet (Fig. 1Q,R). Moreover, this picture remained even after these animals were refed with the chow diet for 4 (Fig. 1S,T), 5 (Fig. 1U,V) or 6 days (Fig. 1W,X). It is important to note that the interval between the HFHS diet consumption by the animals from the HFHSw group was 6 days, *i.e.*, after 12 weeks of treatment, HFHSw mice presented a sustained glucose intolerance.

**Figure 2:**
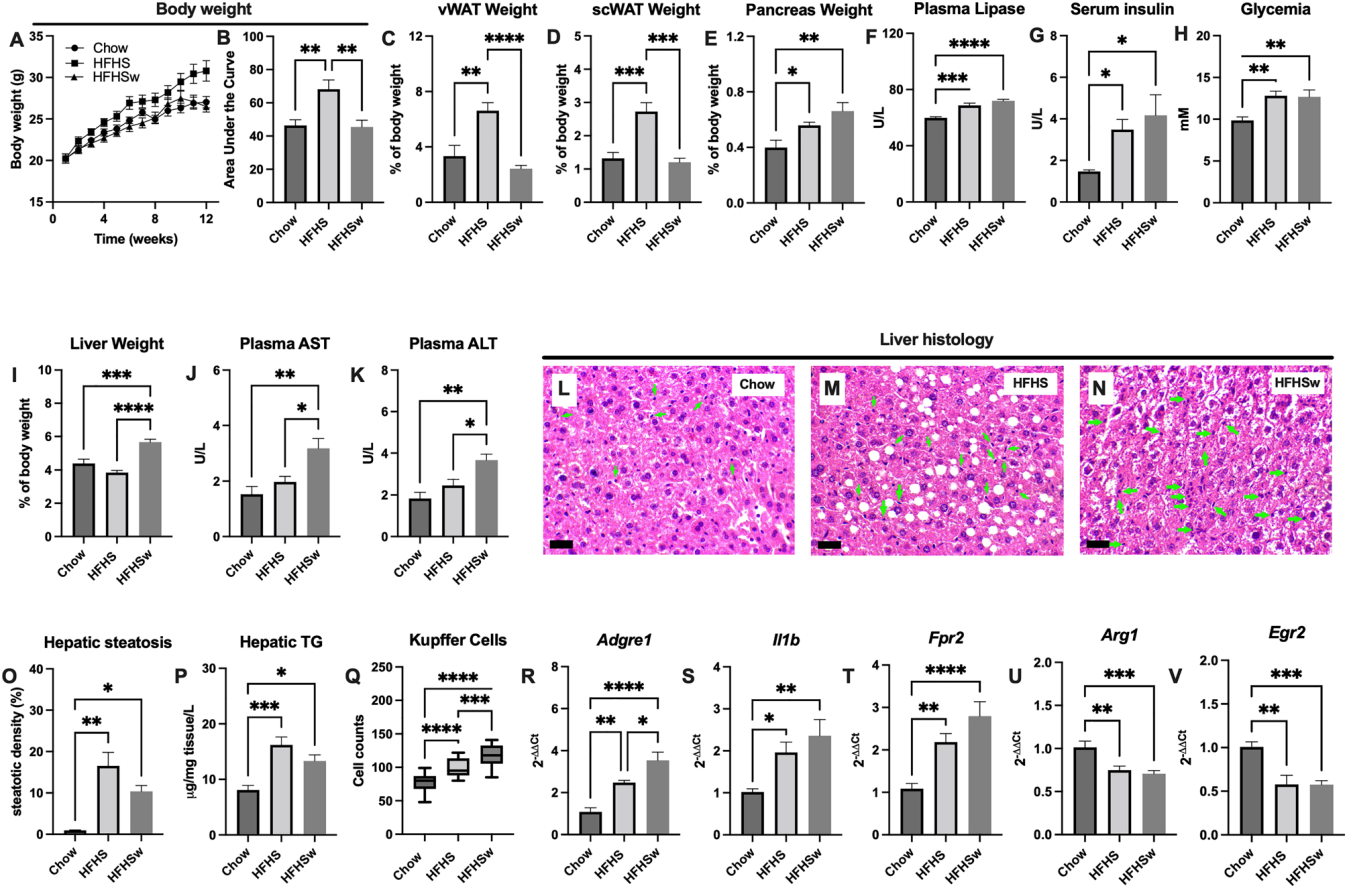
Effects of HFHSw treatment on body mass distribution, plasma biochemistry and liver steatosis and inflammation. Panel **A**: Body weight measurements during the protocol. Panel **B**: area under the curves presented on Panel **A**. The weight of epidydimal WAT (Panel **C**), inguinal WAT (Panel **D**) and pancreas (Panel **E**). Panel **F**: plasma lipase levels. Panel **G**: serum insulin levels. Panel **H**: fasting glycemia before euthanasia. Panel **I**: weight of the liver. Plasma levels of AST (Panel **J**) and ALT (Panel **K**). Representatives H&E stained micrographies from the livers of Chow (Panel **L**), HFHS (Panel **M**) and HFHSw (Panel **N**) groups. The black bars in the bottom left of Panels **L**–**N** represent a 50 μm scale. The green arrows in Panels **L**–**N** indicate some identified Kupffer cells. Panel **O**: percentile of hepatic steatosis. Panel **P**: hepatic triglycerides. Panel **Q**: Kupffer cells count. Panel The authors declare no competing interests. Panel **R** mRNA quantification for *Adgre1*. Panel **S**: mRNA quantification for *Il1b*. Panel **T**: mRNA quantification for *Fpr2*. Panel **U**: mRNA quantification for *Arg1*. Panel **V**: mRNA quantification for *Egr2*. All plotted values are mean ± S.E.M of independent animals. For panels A-K, n = 24. For Panels O-Q, n = 8. For Panels R-V, n = 12. *P < 0.05; **P < 0.01; ***P < 0.001; ****P < 0.0001, One-way ANOVA followed by Tukey’s post-test.

Despite being intolerant to glucose, the HFHSw mice did not gain weight compared to Chow group (Fig. 2A,B). Animals from the HFHS group gained on average 47% more weight than those from the Chow group, while the HFHSw mice presented no significant difference in weight gain compared to Chow (Fig. 2A,B). A similar pattern is observed when analyzing the weight of visceral white adipose tissue (vWAT; Fig. 2C) and sub-cutaneous white adipose tissue (scWAT; Fig. 2D). In contrast, the pancreas weight was increased in both, HFHS and HFHSw groups, as compared to the Chow group (Fig. 2E), which might reflect the increased plasma lipase levels in HFHS and HFHSw groups, as compared to Chow (Fig. 2F). In addition, serum insulin (Fig. 2G) and glycemia (Fig. 2H) were also elevated in both, HFHS and HFHSw groups, as compared to the Chow group. These last data, together with the impaired oGTT, support that HFHS and HFHSw mice were resistant to insulin at the end of the protocol. Conversely, the relative weight of the livers from HFHSw mice was higher than those from Chow and HFHS groups (Fig. 2I). Please notice that these results are expressed as tissue weight relative to the body weight, and since the HFHS group gained more body weight, the relative liver weight was similar to those from Chow group, despite the fact that the net weight of the livers of HFHS group is higher than the Chow group. Enhanced liver weight is reflected in the levels of AST (Fig. 2J) and ALT (Fig. 2K) that were elevated in the HFHSw group as compared to Chow or HFHS. The results presented so far are indicative of increased liver damage in the HFHSw group. Indeed, the histological analyses of the livers from Chow (Fig. 2L), HFHS (Fig. 2M) and HFHSw (Fig. 2N) groups revealed that both HFHS and HFHSw groups presented steatotic livers, as compared to Chow (Fig. 2O). Hepatic steatosis was confirmed by evaluating the triglycerides levels in these livers (Fig. 2P). Moreover, the livers from HFHS and HFHSw groups presented elevated Kupffer cells infiltration, as evaluated by counting these cells in histological analyzes (Fig. 2Q), as well as by evaluating the expression of *Adgre1* mRNA (coding for Kupffer cells membrane protein marker F4/80) in the livers (Fig. 2R). It is important to note that Kupffer cells infiltration in the livers of HFHSw mice were elevated as compared to the HFHS group too (Fig. 2Q,R). In addition to the increased Kupffer cells infiltration, we also observed an increase in the expression of proinflammatory M1 markers *Il1b* (Fig. 2S) and *Fpr2* (Fig. 2T) as well as a decrease in the expression of resolutive (M2) markers *Arg1* (Fig. 2U) and *Egr2* (Fig. 2V) in HFHS and HFHSw groups as compared to Chow.

**Figure 3:**
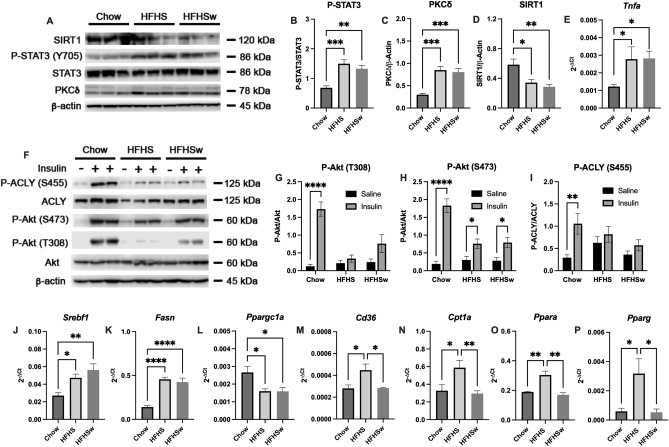
Effects of HFHSw treatment on regulation of liver inflammation, insulin signaling and fat metabolism**.** Panel **A**: representative Western blotting for SIRT1, total and phosphorylated STAT3 and PKCδ. β-actin was used as loading control. Five Western blots for different animals (n = 5) were quantified and results are presented as mean ± S.E.M. for phosphorylated STAT3 (Panel **B**), PKCδ (Panel **C**) and SIRT1 (Panel **D**). Panel **E**: mRNA quantification of *Tnfa* (n = 12). Panel **F**: representative Western Blotting for total and phosphorylated ACLY and AKT in animals that received or not an insulin shot before euthanasia. β -actin was used as loading control. Five Western blots for different animals (n = 5) were quantified and results are presented as mean ± S.E.M. for phosphorylated AKT at Thr308 (Panel **G**), phosphorylated AKT at Ser473 (Panel **H**) and phosphorylated ACLY (Panel **I**). Quantification of mRNA level of *Srebf1* (Panel **J**), *Fasn* (Panel **K**), *Ppargc1a* (Panel **L**), *Cd36* (Panel **M**), *Cpt1a* (Panel **N**), *Ppara* (Panel **O**) and *Pparg* (Panel **P**). For Panels **J**–**P**, samples from 12 different animals were used (n = 12) and results are presented as mean ± S.E.M. *P < 0.05; **P < 0.01; ***P < 0.001; ****P < 0.0001. For Panels **B**–**E** and **J**–**P**, one-way ANOVA followed by Tukey’s post-test was performed. For Panels G-I, two-way ANOVA followed by Šidak’s post-test was performed.

The increased hepatic inflammation is corroborated by phosphorylation of STAT3 at Y705 (Fig. 3A,B) and expression of PKCδ (Fig. 3A,C), which are increased in both HFHS and HFHSw groups, as compared to Chow. The upregulation of PKCδ is confirmed by the downregulation of its downstream target SIRT1 (Fig. 3A,D). Moreover, TNFα, is also upregulated in both HFHS and HFHSw groups, as evaluated by means of *Tnfa* mRNA expression (Fig. 3E). Liver insulin resistance is confirmed by the impaired phosphorylation of Akt at both residues, Thr308 (Fig. 3F,G) and Ser473 (Fig. 3F,H), as well as the impaired phosphorylation of ACLY at Ser455 (Fig. 3F,I), a downstream target of Akt. Additionally, genes related to fatty acid synthesis, such as *Srebf1* (coding for SREBP1c; Fig. 3J) and *Fasn* (coding for Fatty acid synthase; Fig. 3K), are upregulated, while mitochondria biogenesis-related gene *Ppargc1a* (coding for PGC1α; Fig. 3L) is downregulated in HFHS and HFHSw, as compared to Chow. Curiously, genes related to fatty acid catabolism, such as those coding for CD36 (Fig. 3M), CPT1α (Fig. 3N), PPARα (Fig. 3O) and PPARγ (Fig. 3P), are upregulated in HFHS group but not in HFHSw, as compared to Chow.

**Figure 4:**
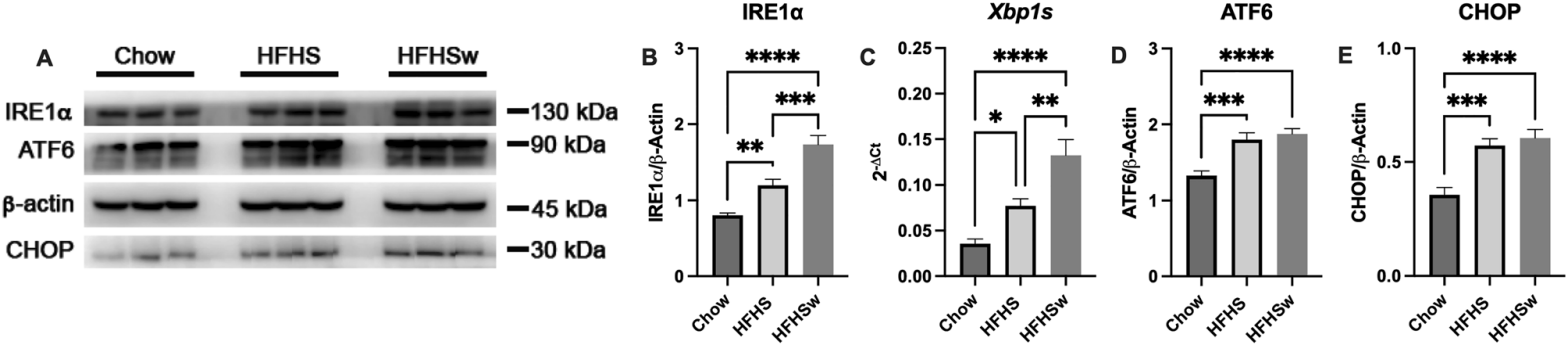
Effects of HFHSw treatment on UPR pathway**.** Panel **A**: representative Western Blotting for IRE1α, ATF6 and CHOP. β-actin was used as loading control. Five Western blots for different animals (n = 5) were quantified and results are presented as mean ± S.E.M. for IRE1α (Panel **B**), ATF6 (Panel **D**) and CHOP (Panel **E**). Panel **C**: mRNA quantification of spliced *XBP1* (mean ± S.E.M., n = 6). *P < 0.05; **P < 0.01; ***P < 0.001; ****P < 0.0001, One-way ANOVA followed by Tukey’s post-test.

Finally, we assessed Unfolded Protein Response (UPR) through endoplasmic reticulum (ER) stress markers in the livers of mice. IRE1α was upregulated in HFHS and HFHSw groups, as compared to Chow (Fig. 4A,B). Interestingly, this protein upregulation in the HFHSw group surpasses the effect of continuous diet observed in the HFHS group (Fig. 4A,B). This result is confirmed by assessing the downstream target of IRE1α RNAse activity, the splicing of *Xbp1* (*Xbp1s*), which is increased in HFHSw as compared to HFHS or Chow groups (Fig. 4C). It is important to note that splicing of *Xbp1* is also increased in the HFHS group, as compared to Chow (Fig. 4C). Moreover, ATF6 is also upregulated in HFHS and HFHSw, as compared to Chow (Fig. 4A,D), indicating the activation of different branches of the UPR pathway. Corroborating these results, C/EBP Homologous Protein (CHOP), a downstream target of the different branches of UPR pathway, was also upregulated upon both treatments, HFHS and HFHSw (Fig. 4A,E), confirming that the continuous low-frequency HFHS consumption promotes and sustains several markers of Western diet-induced insulin resistance in mice.

## Discussion

The world is facing pandemics of obesity which have more than tripled in the last 50 years, reaching a global prevalence of 41.5% for central obesity, *i.e.*, a waist circumference of greater than 94 cm and 80 cm for males and females, respectively^22,23^. It is a consensus that obesity must be treated, especially among children, which have presenting the most significant increase in prevalence during the last decade^24–26^. Although considered to be largely preventable, obesity is mostly caused by the existence of an obesogenic environment, which is defined by consumption of UPF associated with low levels of physical activity^27,28^. Indeed, the obesogenic environment is especially dangerous among children due to their in-development capacity to identify the better food and physical activity^29,30^. Among several approaches, nutritional education is recognized as an efficient method to change the obesogenic environment and treat obesity among families^31^.

The “Cheat Meal” is an emerging dietary behavior where people are allowed to sporadically consume a large amount of food in a day, claiming that this uncontrolled eating behavior would not impact health or body adiposity^32,33^. A recent study about eating disorders among adolescents and young adults (30 years old or less) involving more than 2000 individuals revealed an engagement with “cheat meal” of more than 70% of participants^34^. This behavior is explained, since different social media publications have claimed that “cheat meals” promote muscle mass gain and weight loss, without any scientific basis for this^32^. Notwithstanding, it is very common that, at least once a week, people just relax and consume some kind of “junk food”. Indeed, “junk food” consumption has increased during the SARS-CoV-2 pandemics, such as obesity^35^. Despite the clear correlation between “junk food” consumption and obesity growth, there is no study evaluating the effects of occasional (and why not to say, more acceptable) consumption of high-caloric foods (“junk food”) on the co-morbidities associated to the exposition to an obesogenic environment.

Many animal models have been used to study the effects of obesogenic environments, especially the consumption of high-caloric Western diets^11–14^. These diets are rich in saturated fat and simple sugar, especially sucrose, and thus are comparable to the so-called “junk food”^35^. However, evaluation of the effects of Western diet on animal health is achieved by the constant consumption of these diets^11–14^, which is not what normally happens^15^. In the current work, we have mimicked “cheat meal” consumption by giving to mice a HFHS diet once a week, somehow comparing it to people that sporadically eat “junk food”. It has been described that the punctual or short-term consumption of fat and/or sugar enriched diets impairs glucose utilization^18,20,21,36–39^. Our data here show that consumption of HFHS diet for 24 h impairs glucose tolerance tests in mice. This effect occurs in a shorter period as compared to the 3–4 days reported effect for similar diets^20,39^. Nevertheless, our group has reported that citrate added as a food additive can promote similar effects in the same time period^21^. However, to our knowledge, none of these works have tested the durability of the effects of these short-term treatments. Here, we showed that oGTT impairment induced by acute (24 h) HFHS diet was reversed after 24 h of regular chow ingestion. The reversal of diet-induced insulin resistance can be achieved by changing diet, promoting physical exercise, pharmacologically, among other approaches^40–42^. However, this reversal is a time-dependent process that also depends on the severity of the condition^43^. Thus, it was expected that acutely induced GTT impairment would be shortly reversed after revoking the obesogenic environment, in this case, the HFHS diet. Nevertheless, when this acute exposition to the obesogenic environment was hebdomadally repeated, the reversal of GTT impairment required longer revoking periods, such as after twelve consecutive weeks, a 6-days consumption of regular normal diet was not enough to reverse GTT impairment. Pragmatically, this means that, after longer periods of a once-a-week consumption of “junk food” can sustainably induce insulin resistance, at least to some extent.

The comparison between animals that were fed continuously with the HFHS diet and those that consumed the diet once-a-week gives very interesting results. For instance, although they are equivalently resistant to insulin, animals from the HFHSw group did not gain as much weight as the HFHS group, on the contrary, their weight gain during the protocol was compared to the control Chow group. This same difference in weight gain can be seen in the weight of both visceral and subcutaneous white adipose tissues. Recently, we have reported that mice fed for 12 weeks with an HFHS diet enriched with citrate have also developed insulin resistance without gaining weight, especially in white adipose tissues^44^. Here, despite not gaining as much weight as the HFHS group, animals from the HFHSw group displayed an augmented liver weight as compared to both, Chow and HFHS groups. Moreover, the elevated plasma AST and ALT in HFHSw as compared to the other two groups suggests increased liver damage in HFHSw, even when compared to animals that received the diet continuously (HFHS group). This increased liver damage is supported by assessing Kupffer cells infiltration, which is higher in HFHSw group as compared to HFHS or Chow groups (note that Kupffer cells infiltration is higher in HFHS group as compared to Chow group, as expected). Regardless of administered continuously or once-a-week, consumption of HFHS favored the polarization of infiltrated Kupffer cells to a pro-inflammatory M1 profile, which is associated with liver damage^45^, explaining the above mentioned effects. Nevertheless, other pro-inflammatory markers, such as IL1β and TNFα, were equally upregulated in both HFHS and HFHSw as compared to Chow. The effects of increased TNFα are directly related to cellular insulin resistance, and these effects are potentiated by PKCδ^46,47^. This later enzyme is upregulated in animals under HFHS diet^48^, being correlated to insulin resistance^48^, liver inflammation^49^, and non-alcoholic steatohepatitis^50,51^. Moreover, PKCδ promotes downregulation of SIRT1, which is also related to insulin resistance, leading to liver damage and fibrosis^49^. Furthermore, liver inflammation can be triggered by phosphorylated STAT3, which also induces the lack of liver responsiveness to insulin^52^. In the current work, insulin-induced phosphorylation of AKT at Thr308 was impaired in HFHS and HFHSw groups, as compared to Chow. This phosphorylation occurs via PDK activation by PI3K, which in turn is activated directly downstream to insulin receptor activation^53^. However, the second AKT phosphorylation, at Ser473, which is mediated by mTORC2^53^, was still responsive to insulin in the three groups, at the end of the treatment. Nonetheless, phosphorylation of ACLY at Ser455, which is directly downstream to AKT^53^, was also impaired in HFHS and HFHSw groups, supporting that AKT signaling is compromised in these two groups. All these effects were observed in the current work and were equally responsive in HFHS and HFHSw groups, supporting that, even when occasionally taken, Western diet favors liver inflammation, insulin resistance and damage.

The lipid metabolism (synthesis and degradation) is an insulin-dependent process and thus is entirely compromised during insulin resistance. In the current work, we observed that genes related to lipid anabolism (*Srebf1*, coding for SREBP1c, and *Fasn*, coding for fatty acid synthase) were upregulated in both, HFHS and HFHSw groups as compared to Chow group. On the other hand, *Ppargc1a*, coding for PGC1α, a major regulator of mitochondria biogenesis and thus fatty acid catabolism^54^, is downregulated in both HFHS and HFHSw groups. These anabolic genes are normally upregulated in the liver of mice submitted to long periods under high-fat diets^55^, while PGC1α is normally downregulated under the same conditions^56^. Here, we show that all these regulations occur after 12-weeks of treatment with HFHS diet, regardless of consuming the diet continuously or once-a-week. On the other hand, other genes related to lipid catabolism, such as *Cd36*, *Cpt1a*, *Ppara* and *Pparg*, coding for the plasma membrane fatty acid transporter, carnitine-palmitoyl transferase, PPARα and PPARγ, respectively, were upregulated only in HFHS group, while their expressions in HFHSw group were comparable to the Chow group. Among these results, the expression pattern of *Ppara* and *Pparg* correlates with weight gain by these animals, since upregulation of both is related to redistribution of fat to white adipose tissues^57^.

The ER is a nutrient sensing organelle which adapts to metabolic changes aiming to regulate cellular homeostasis. Metabolic disorders, especially obesity-induced insulin resistance, are normally associated with disturbances in ER homeostasis in a process known as “ER stress” or the unfolded protein response (UPR) pathway^58–60^. ER stress can lead to different signals that contribute to severity of insulin resistance and cellular inflammation^58–60^. Here, we showed that different markers of ER stress were triggered after the treatment with the HFHS diet once-a-week for 12 weeks. Some of them, IRE1α and its downstream effector spliced *Xbp1* (*Xbp1s*), were elevated in HFHSw group as compared to HFHS group, supporting that the occasional consumption was more adverse than continuous consumption of HFHS. This effect can be explained since ER stress depends on homeostasis which is more perturbed after punctual stimulus than after continuous stimulation. Indeed, we propose here that the repetitive perturbation of cellular homeostasis by changing the diet to a highly obesogenic one is promoting stable and sustained insulin resistance and liver inflammation, supporting induction of NAFLD in mice.

**Figure 5:**
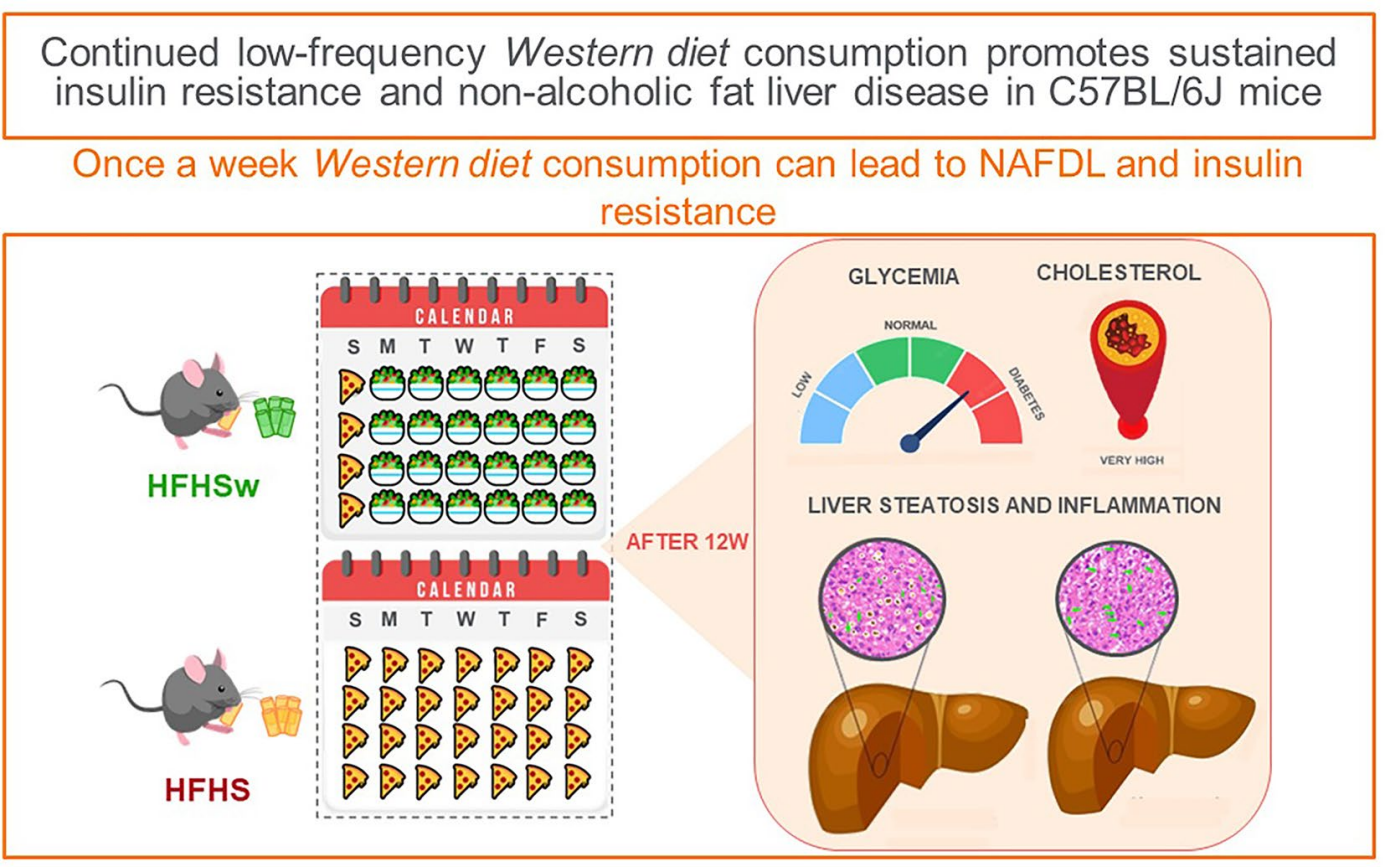
Graphical summary of the findings**.**

Finally, we suggest here that regular consumption of UPF, even when not continuously, can lead to NAFLD and insulin resistance, which is summarized in Fig. 5. Our results suggest that punctual, or sporadic, consumption of UPF, due to the high content in simple sugar and saturated fat, can trigger a transient impaired oGTT, which is indicative of insulin resistance. However, the weekly repetition of the insult turns this transient effect into a sustained one, compatible with a continuous consumption of UPF. Additionally, based on our previous works^21,44,61,62^, it is very possible that food additives, which are highly present in UPF, would aggravate diet-induced insulin resistance and NAFLD. Therefore, our work sounds a note of caution about consumption of UPF, even when it is sporadically consumed.

## Methods

### Animal protocol

Animal protocol was performed according to the criteria approved by the Animal Care and Use Committee from the Health Sciences Center of the Federal University of Rio de Janeiro (CEUA/CCS/UFRJ 177/18). All information reported here were elaborated according to the ARRIVE guidelines^63^. For this study, seventy-two C57BL/6 J mice (6-weeks-old males; acquired from CEMIB, UNICAMP, Campinas, SP, Brazil; the appropriate sample size was calculated according to previously described^64^) were acclimated for one week, where they were individually housed and received AIN-93 M chow pellets and water ad libitum. During the protocol, animals were kept under controlled conditions (24 ± 1 °C; 55 ± 15% humidity; and 12 h/12 h light/dark cycle). After the acclimation period, animals were randomly divided into three groups of 24 animals each that were subjected to the different treatments proposed as follows. The control group, named Chow, continuously received a powdered chow diet for twelve continuous weeks. A second group continuously received a HFHS diet and was named as HFHS group. The third group received this same HFHS diet for 24 h, once-a-week, and was fed the powdered chow diet for the rest of the week. This later group was named after a weekly treatment with an HFHS diet as HFHSw group. A schematic representation of the protocol is presented in Supplementary Material (Fig. S1). The HFHS diet used contained 40% (w/w) fat and 27% (w/w) sucrose and was prepared as previously described^60^. HFHS diet content is available in Table S1. Food intake was measured each day at 6:00 am by weighing the remaining food. Water was given ad libitum during the entire protocol. For the oral glucose tolerance tests (oGTT), groups of 6 animals from each group were randomly chosen and submitted to the test to not repeat the test with the same animal in an interval shorter than two weeks. The oGTTs were performed simultaneously for the three groups in the day after the HFHSw receive the HFHS diet and in the subsequent days until reversal of oGTT impairment in HFHSw group (tests were performed after the first 24 h, and after 1, 6 and 12 weeks of the protocol. By the end of the protocol, animals were euthanized, and serum and tissues were collected and weighed. Small samples of the liver were taken for microscopy and fixed with buffered formalin (4% weight/vol formalin, 0.4% weight/vol NaH_2_PO_4_, 0.65% weight/vol Na_2_HPO_4_), and the remaining material was immediately frozen in liquid N_2_. Frozen livers were powdered in liquid N_2_ using a ceramic mortar and pestle. This powdered material was kept under -80 °C until its final use. At euthanasia, each group was randomly subdivided into two sub-groups (n = 12) to receive an intra-venous injection of either saline (0.9% weight/vol NaCl) or 2 U/kg insulin aiming to evaluate insulin signaling. Plasma alanine aminotransferase (ALT), plasma aspartate aminotransferase (AST), plasma lipase and serum insulin were evaluated as described previously^21^.

### Oral glucose tolerance test

Tests were performed as previously described^21^. Briefly, animals were fasted for 5 h before performing the oGTT, where 2 g/Kg dextrose was administered by gavage. Blood samples were collected from the caudal vein immediately before and 15, 30, 60 and 120 min after administration of a single dose of dextrose. Glycemia was evaluated using a FreeStyle Precision Neo glucometer (Abbott Laboratories, Chicago, IL, USA).

### Western blotting

Western blotting was conducted as previously described^21^. Briefly, total proteins of liver were extracted from approximately 50 mg of powdered tissues using a mild-extraction RIPA buffer (50 mM Tris–HCl pH 7.5, 150 mM NaCl, 1 mM EDTA, 1 mM EGTA, 1% vol/vol NP-40, 10 mM NaF, 2 mM Na_3_VO_4_, 2.5 mM Na_4_P_2_O_7_, 1 mM b-glycerophosphate) supplemented with protease inhibitor cocktail (Sigma-Aldrich, St. Louis, MO, USA). After homogenization and centrifugation (15 min, 8000 x*g*), supernatants were submitted to SDS-PAGE (8% acrylamide gels) followed by overnight transfer to nitrocellulose membranes at 30 V. Membranes were stained with Ponceau S, processed by precutting the blots and de-stained by washing with distilled water. The precut membranes were used for hybridization with the different antibodies, thus we are not able to provide full-length stained membranes, but originals and replicate precut stained membranes can be found in Supplementary Material to this publication. Incubation with primary antibodies was performed overnight. Afterwards, membranes were washed and incubated for 1 h with the appropriate secondary antibody and stained using Amersham ECL Western Blotting Reagent (Cat# RPN2124, GE Healthcare Biosciences, Pittsburg, PA, USA). Staining was evaluated using a C-DiGit Blot Scanner (LiCor, Lincoln, NE, USA) and quantifications of the blots were performed using the software Image J64 (Rasband, W.S., ImageJ, U. S. National Institutes of Health, Bethesda, Maryland, USA, https://imagej.nih.gov/ij/, 1997–2018; NIH, USA)^65^. The complete list of antibodies used is presented in Table S2.

### Quantitative PCR (qPCR)

Total RNA was extracted from approximately 50 mg of powdered liver using Trizol (ThermoFisher, Carlsbad, CA, USA) as previously described^21^. Total RNA was used to synthesize cDNA using High-Capacity cDNA Reverse Transcription Kit (ThermoFisher, Carlsbad, CA, USA). All qPCR reactions were performed on a QuantiStudio 5 apparatus (ThermoFisher, Carlsbad, CA, USA) by using probe-based TaqMan Universal Master Mix II with UNG (ThermoFisher, Carlsbad, CA, USA) or dye-based GoTaq qPCR Master Mix (Promega, Fitchburg, WI, USA) according to the oligo pairs availability. The programs for dye-based reactions are described elsewhere^21^. Relative mRNA expression of all genes of interest was calculated based on ribosomal protein L7 (*Rpl7*) expression, here used as reference gene, calculated by the 2^-^${\Delta}$^Ct^ or 2^-^${\Delta}$^Ct^ method, as described previously^66^, following high standards for the technique^67^. The complete list of oligos pairs used is presented in Table S3.

### Histology

Samples from the liver were fixed in buffered formalin and stained with hematoxylin and eosin (H&E) for histopathological analyses, as previously described^21^. Kupffer cells were identified and counted using a cell counting device as previously described^21^. Average steatotic area was evaluated by using ImageJ64 software (Rasband, W.S., ImageJ, U. S. National Institutes of Health, Bethesda, Maryland, USA, https://imagej.nih.gov/ij/, 1997–2018; NIH, USA)^65^., as previously described^68^.

### Statistics

All graphs and statistical analyses were performed with software GraphPad Prism version 9 for Mac (GraphPad Software, San Diego, CA, USA, www.graphpad.com). Student’s t-test, one-way ANOVA followed by Tukey’s post-tests, or two-way ANOVA followed by Šidak’s post-test were used as appropriate.

### Ethical approval

Animal protocol was performed in accordance to the fundamental principles indicated by the International Council for Laboratory Animal Science (ICLAS) and the criteria approved by the Animal Care and Use Committee from the Health Sciences Center of the Federal University of Rio de Janeiro receiving the following approved protocol number: CEUA/CCS/UFRJ 177/18.

## Supplementary Information

Supplementary Information.

## Data Availability

All data and resources are available upon request to the correspondence author.

## Abbreviations

ALT: Alanine aminotransferase;
AST: Aspartate aminotransferase
CHOP: C/EBP Homologous protein
CVD: Cardiovascular disease
ER: Endoplasmic reticulum
H&E: Hematoxylin and eosin
HFHS: High-fat high-sucrose
HFHSw: Group of animals treated with HFHS diet once-a-week
MS: Metabolic syndrome
NAFLD: Non-alcoholic fat liver disease
NASH: Non-alcoholic steatohepatitis
oGTT: Oral glucose tolerance test
qPCR: Quantitative real-time polymerase chain reaction
scWAT: Subcutaneous white adipose tissue
T2DM: Type-2 diabetes mellitus
UFP: Ultra-processed foods
UPR: Unfolded protein response
vWAT: Visceral white adipose tissue
WAT: White adipose tissue
