# Supplementary material for: Once a week consumption of Western diet over twelve weeks promotes sustained insulin resistance and non-alcoholic fat liver disease in C57BL/6 J mice
Source: Sci Rep. 2023 Feb 21;13:3058. doi: 10.1038/s41598-023-30254-2 (PMC9942638; doi:10.1038/s41598-023-30254-2)
Supplement: Supplementary file 1 — Supplementary Information. [file 41598_2023_30254_MOESM1_ESM.pdf]

Supplementary Material to:

Continued low-frequency Western diet consumption promotes sustained insulin resistance and non-alcoholic fat liver disease in C57BL/6J mice

Thainá Magalhães Demaria, Leticia Diniz Crepaldi, Emylle Costa-Bartuli, Jessica Ristow Branco, Patricia Zancan and Mauro Sola-Penna

The metaboliZSm' grouP, Departamento de Biotecnologia Farmacêutica, Faculdade de Farmácia, Universidade Federal do Rio de Janeiro, Rio de Janeiro, RJ, 21941-902, Brazil. Instagram: @ZSPGroup

Keywords: Ultra-processed food; type 2 diabetes; intermittent hypercaloric diet; NAFLD; inflammation

Correspondence: Mauro Sola-Penna, Departamento de Biotecnologia Farmacêutica, Faculdade de Farmácia, Universidade Federal do Rio de Janeiro, Rio de Janeiro, RJ, 21941-902, Brazil. msolapenna@me.com; @maurosolapenna

Table S1: Chow and High-Fat High-Sucrose (HFHS) diets composition

Diet	Chow			HFHS		
**Ingredients**	**g/100g**	**Energy provided (kcal/100g)**	**Energy (%)**	**g/100g**	**Energy provided (kcal/100g)**	**Energy (%)**
Casein	20.00	80.0	21.5	20.00	80.0	14.7
L-cystine	0.18	0.7	0.2	0.18	0.7	0.1
Sucrose	10.00	40.0	10.8	28.69	107.6	19.8
Alpha-cellulose	5.00			5.00		
Starch	40.00	160.0	43.0			
Dextrinized Starch	13.09	53.4	14.3			
Mineral mix (AIN-93)	3.50			4.90		
Vitamin mix (AIN-93)	1.00			1.40		
Lard	-			19.80	178.2	32.7
Soy oil	7.00	38.1	10.2	19.80	178.2	32.7
Choline bitartrate	0.20			0.20		
Tert-butylhydrytoluene (BHT)	0.03			0.03		
Total	100.00	372.2	100	100.00	544.7	100

Table S2. Antibodies used

Antibody	Brand	Catalog #	Dilution
ACLY	Abcam&	40793	1:1000
Akt	Cell Signaling*	9272	1:1000
Anti-mouse	Jackson Labs#	115-035-146	1:10000
Anti-rabbit	Jackson Labs	115-035-144	1:20000
ATF6	SCBT@	22799	1:1000
CHOP	Cusabio$	CSB-PA001639	1:1000
IRE1α	Cell Signaling	3294	1:1000
Phospho-ACLY (S455)	Abcam	46796	1:1000
Phospho-Akt (S473)	Cell Signaling	9271	1:1000
Phospho-Akt (T308)	Cell Signaling	9275	1:1000
Phospho-STAT3(Y705)	Cell Signaling	9131	1:1000
PKCδ	Cell Signaling	2058	1:1000
SIRT1	SCBT	15404	1:1000
STAT3	Cell Signaling	9138	1:1000
β-actin	Cell Signaling	4970	1:1000

* Cell signaling Technology, Danvers, MA, USA

& Abcam, Cambridge, UK

Jackson ImmunoResearch Labs, West Grove, PA, USA

@ Santa Cruz Biotechnology, Santa Cruz, CA, USA

$ Cusabio Technology, Wuhan Shi, China

Table S3. Oligo pairs.

Gene	Forward sequence	Reverse sequence	Amplicon length (bp)	Efficiency (%)
Adgre1	CCGTCAGGTACGGGATGAAT	AGAAGTCTGGGAATGGGAGC	73	97
Arg1	CCTCGAGGAGGGGTAGAGAAA	GGTCTCTCACGTCATACTCTGTTT	91	93
Cd36	AGAATTCTCAGCTGCTCCGC	CACATTTCAGAAGGCAGCAAC	137	109
Cpt1a	TGGACCCAAATTGCAGTGGT	CTCCCACCAGTCACTCACATAA	73	97
Egr2	GTGGCGGGAGATGGCATGAT	TCGGATACGGGAGATCCAGG	70	97
Fasn	CCATGGCAGCTGTTGGTTTG	GTGTCCTCAGAGTTGTGGCA	88	98
Fpr2	CCAGTGATTCAAGCACCAGTT	TCACAGACTTCATGGGGCCTT	71	99
IL1b	AGCTTCAGGCAGGCAGTATC	ACGGGAAAGACACAGGTAGC	70	102
Ppargc1a	CTFTGGAACTGCAGGCCTAA	CAAGAGGGCTTCAGCTTTGG	96	99
Ppara	GCAACAACCCGCCTTTTGTC	TTGGCCACAAGCGTCTTCTC	70	99
Pparg	GCCTATGAGCACTTCACAAGAAAT	GCTGATTCCGAAGTTGGTGG	76	97
Rpl7	GAAGCTCATCTATGAGAAGGC	AAGACGAAGGAGCTGCAGAAC	202	105
Srebf1	GGAGCCATGGATTGCACATT	GGCCCGGGAAGTCACTGT	70	101
Tnfa	TGAGAAGTTCCCAAATGGCCT	CCACTTGGTGGTTTGTGAGTG	74	101
Xbp1	AAGAACACGCTTGGGAATGG	ACTCCCCTTGGCCTCCAC	67	99
Xbp1s	GAGTCCGCAGCAGGTG	GTGTCAGAGTCCATGGGA	65	102

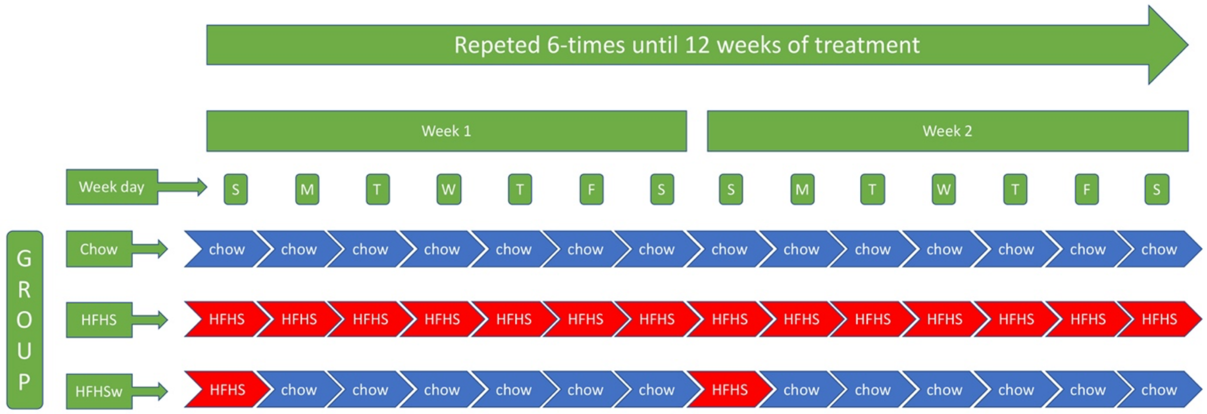

Figure S1. Schematic representation of the animal protocol. The three different animal groups, named Chow, HFHS and HFHSw received different dietetic treatment for 12 weeks. The Chow group continuously received regular chow diet. The HFHS group continuously received the High-Fat High-Sucrose (HFHS) diet, while the HFHSw group received the HFHS diet once-a-week and the regular chow diet for the rest of the week.

Uncropped Gels

Figure 3A Originals cropped membranes

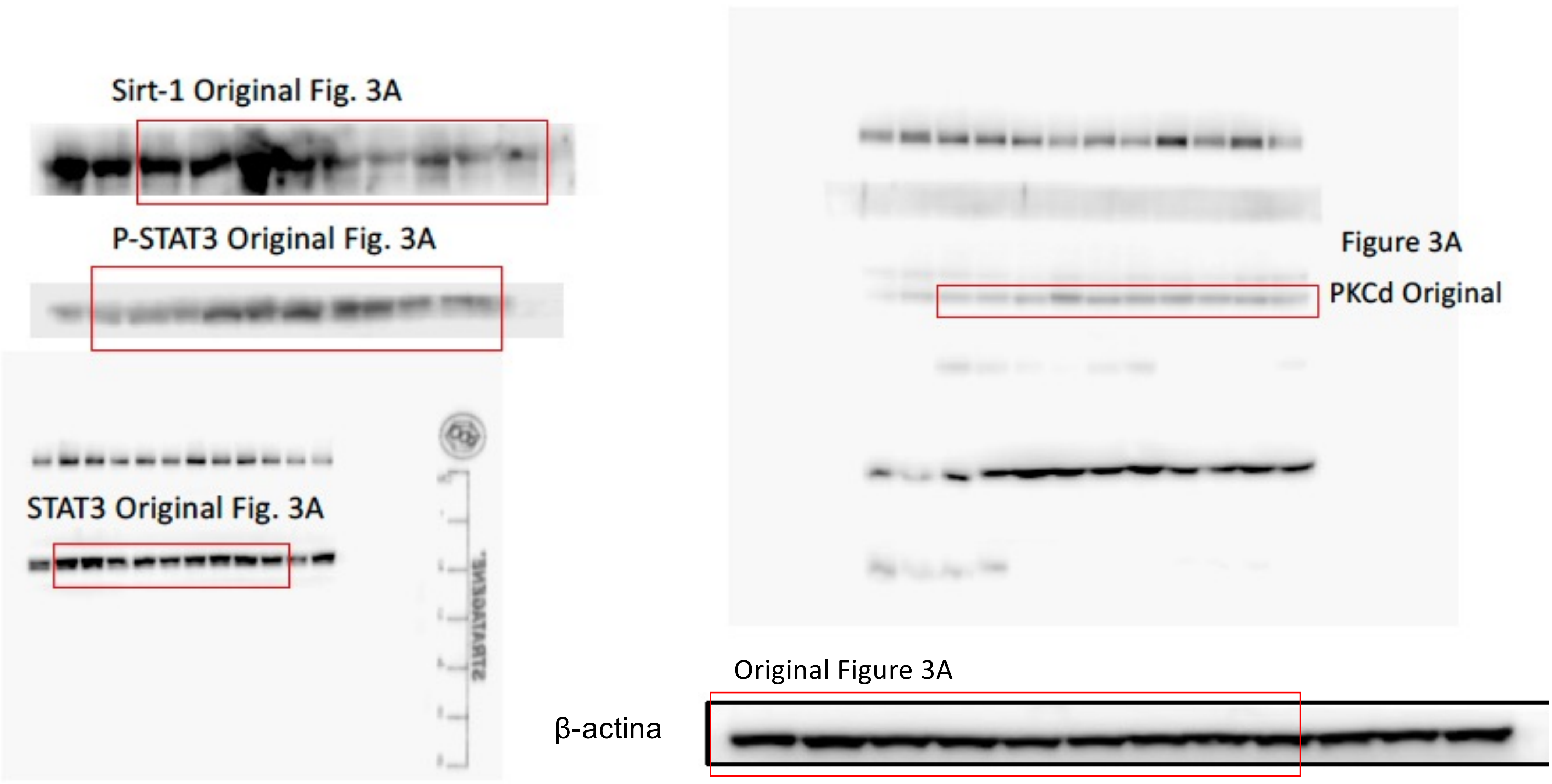

Figure 3F Originals cropped membranes

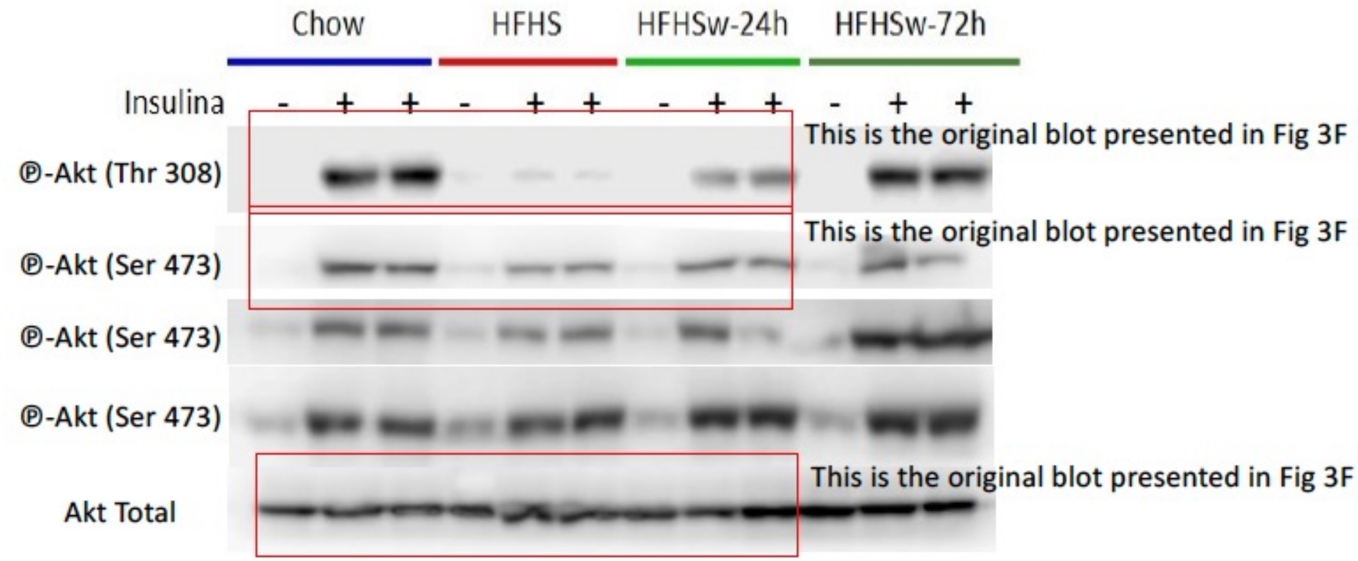

This is the original blot presented in Fig 3F

This is the original blot presented in Fig 3F

This is the original blot presented in Fig 3F

Original Figure 3A

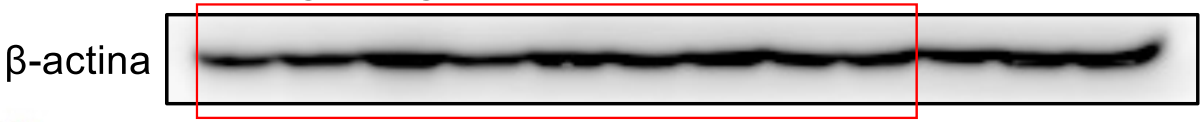

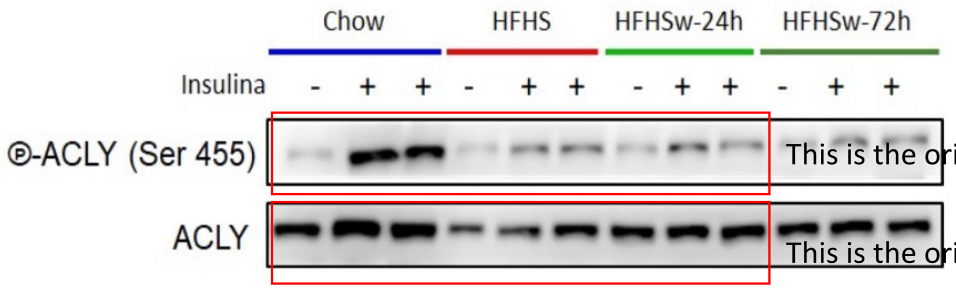

This is the original blot presented in Fig. 3F

This is the original blot presented in Fig. 3F

Figure 4A Originals cropped membranes

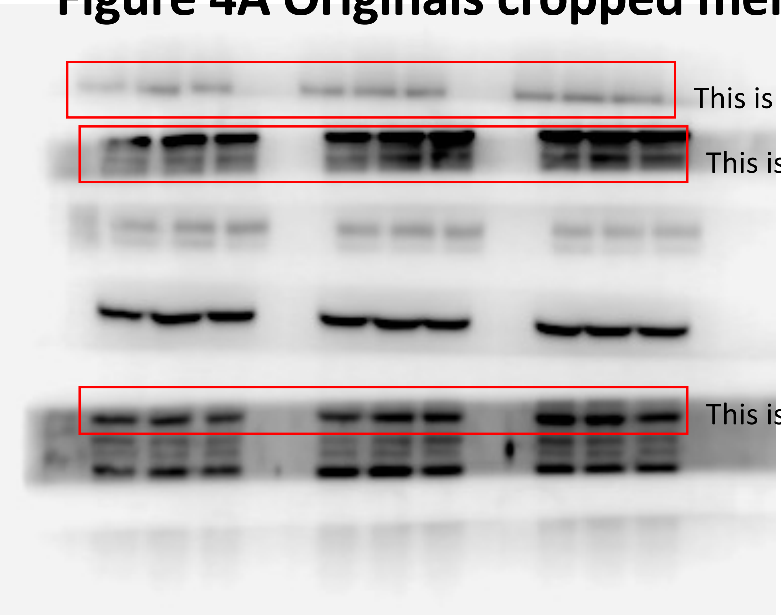

This is the original CHOP blot presented in Fig. 4A

This is the original ATF6 blot presented in Fig. 4A

This is the original IRE1 blot presented in Fig. 4A

This is the original Actib blot presented in Fig. 4A

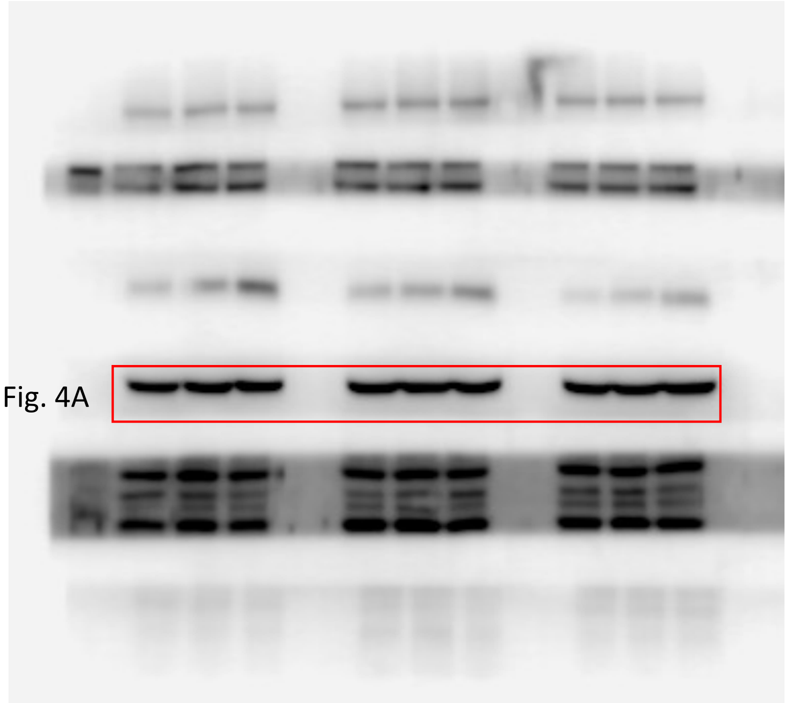

2018

13/03/2018

Gel 1

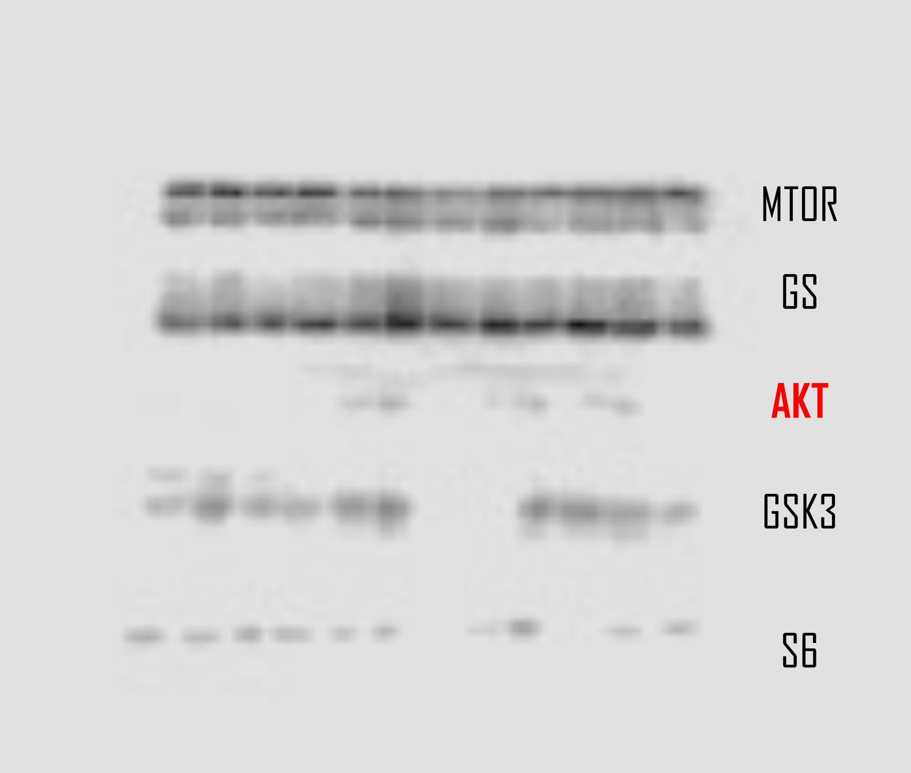

2018

03/09/2018

Gel 3

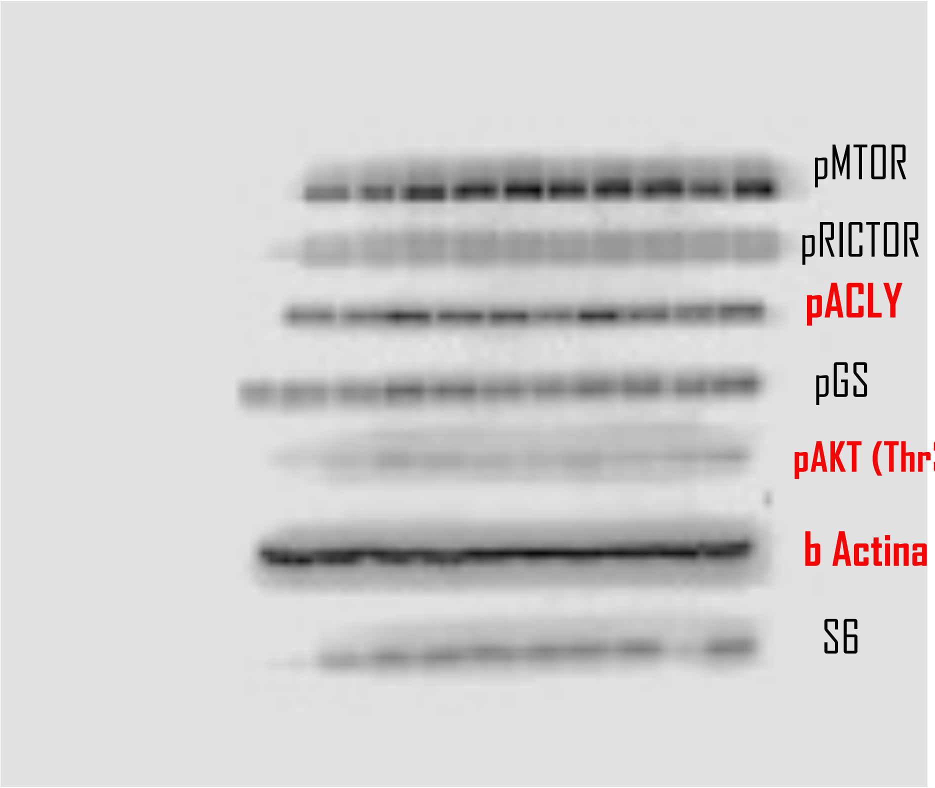

Gel 4

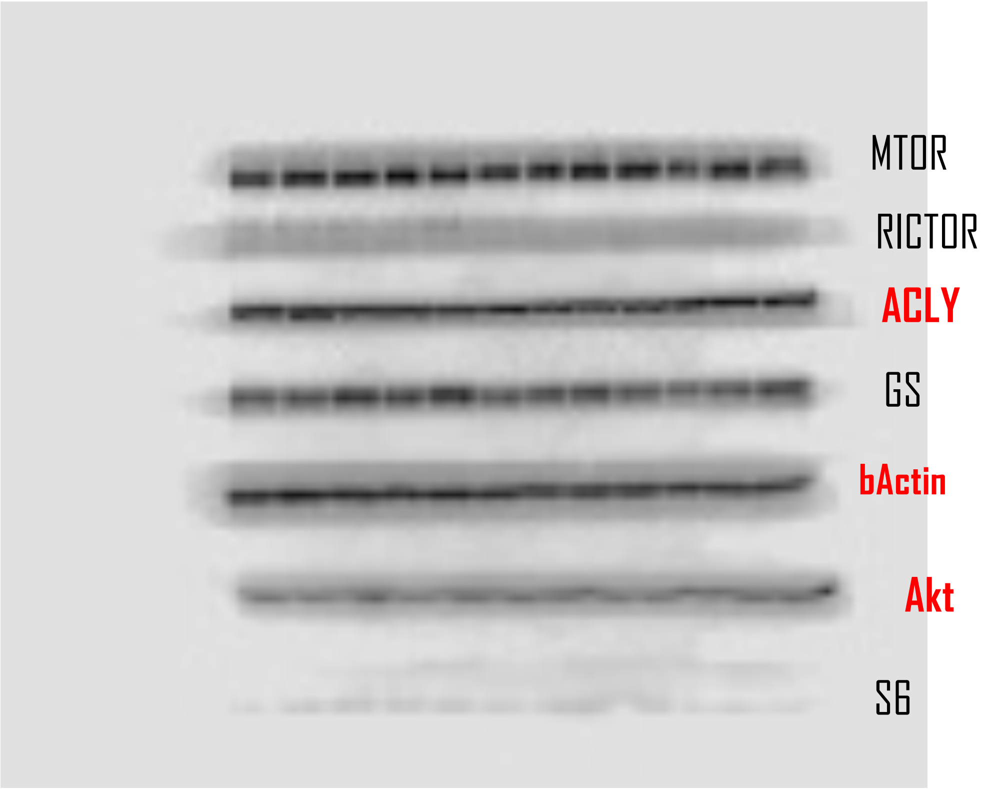

2018

10/04/2018

Gel 1

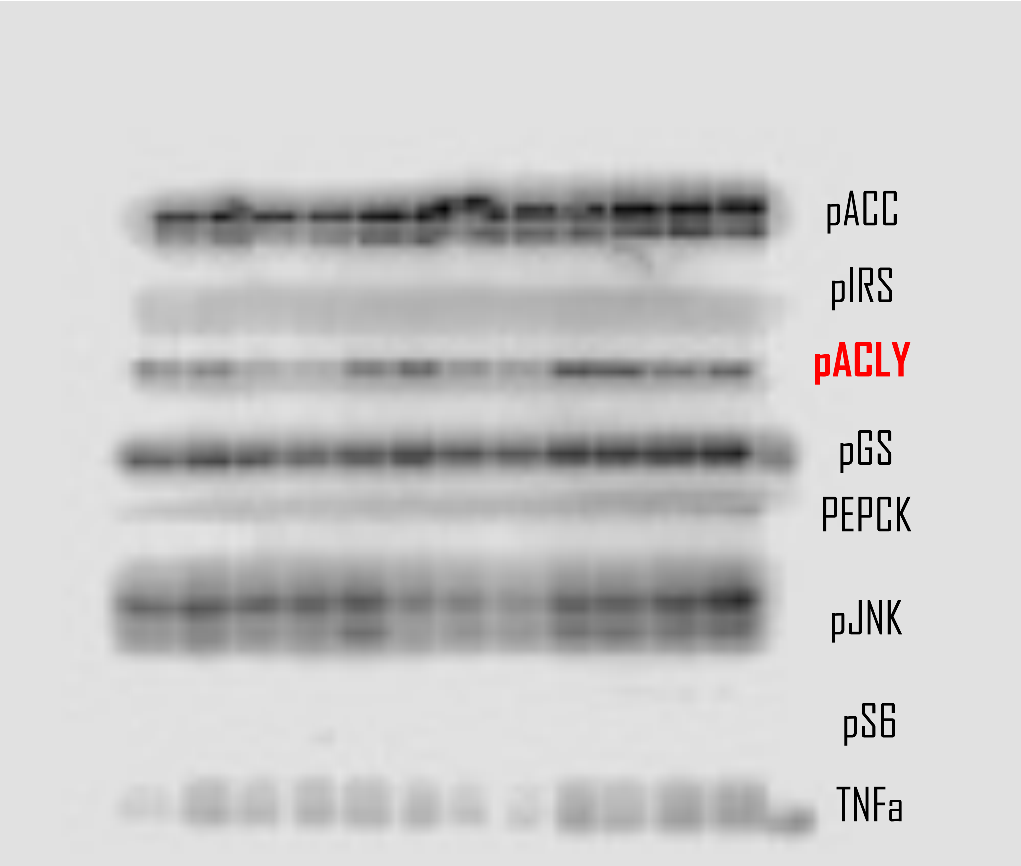

Gel 2

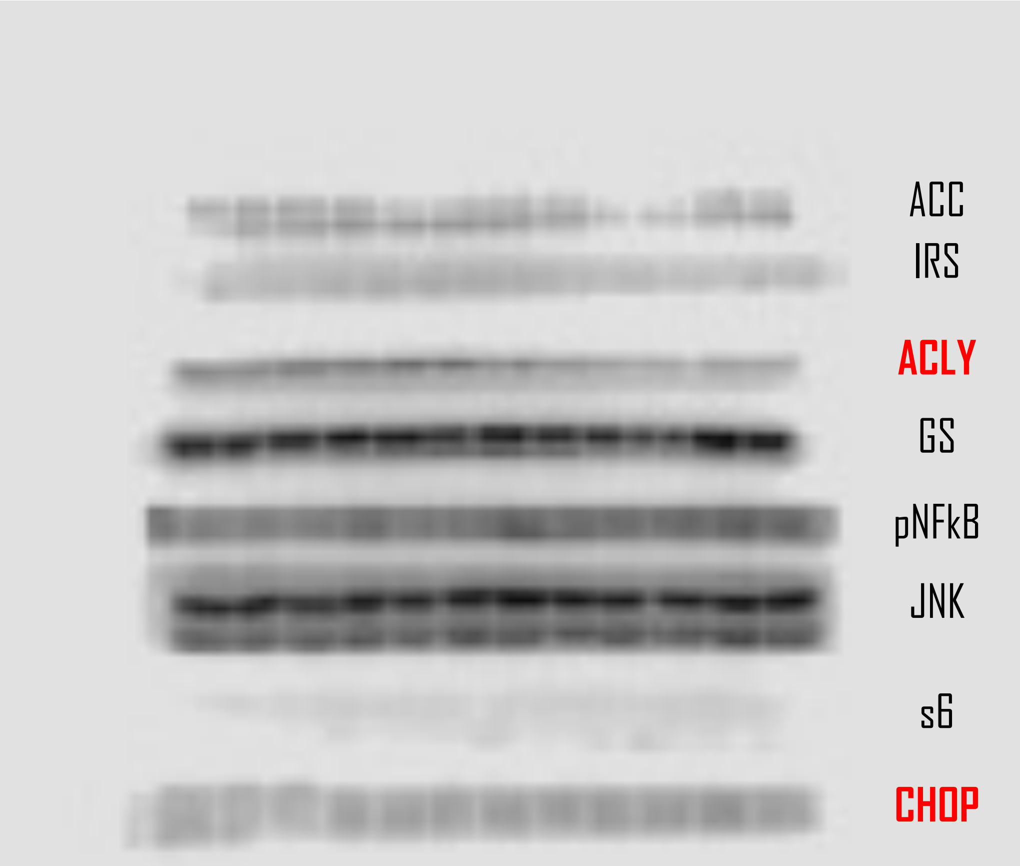

2018

10/04/2018

Gel 3

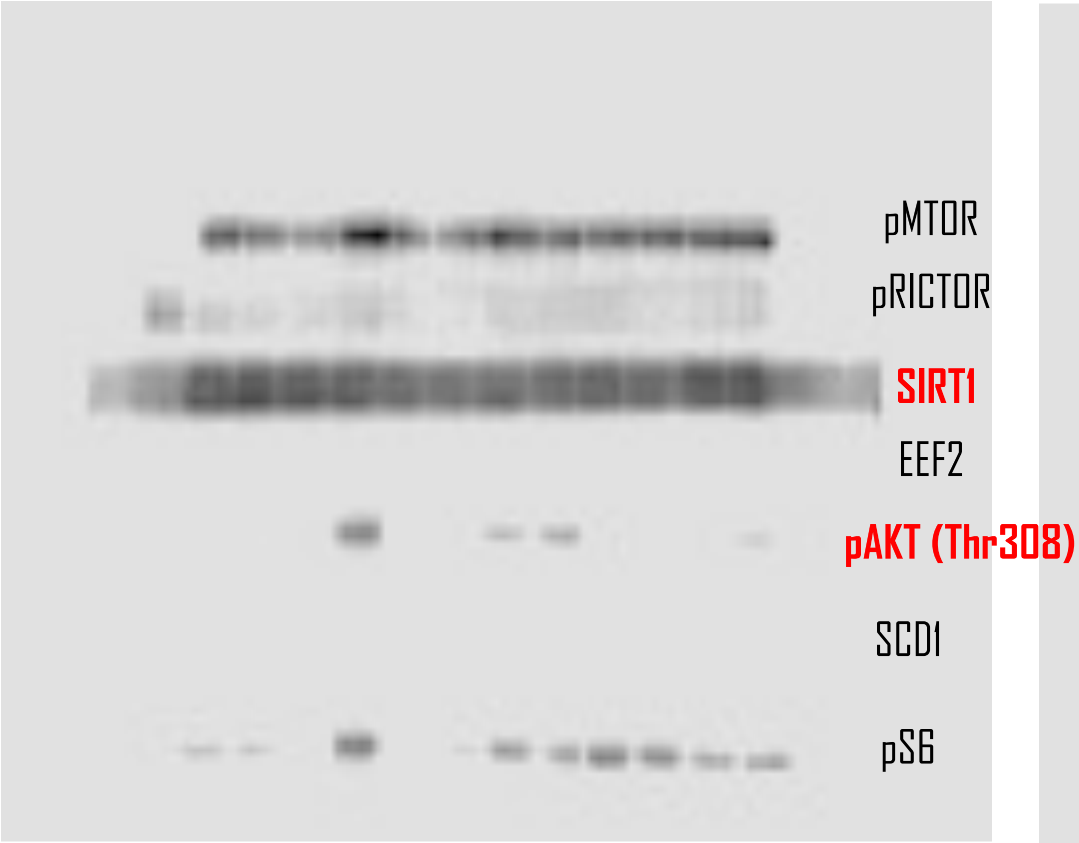

Gel 4

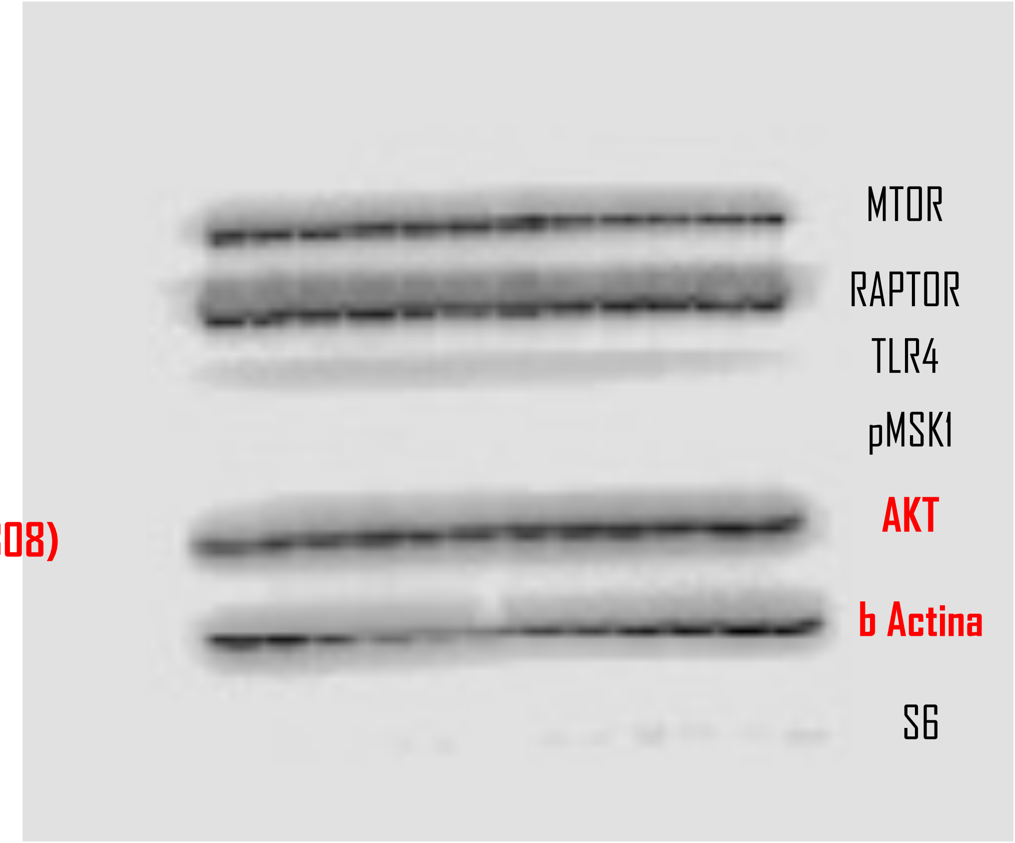

2018

16/04/2018

Gel 1

Gel 2

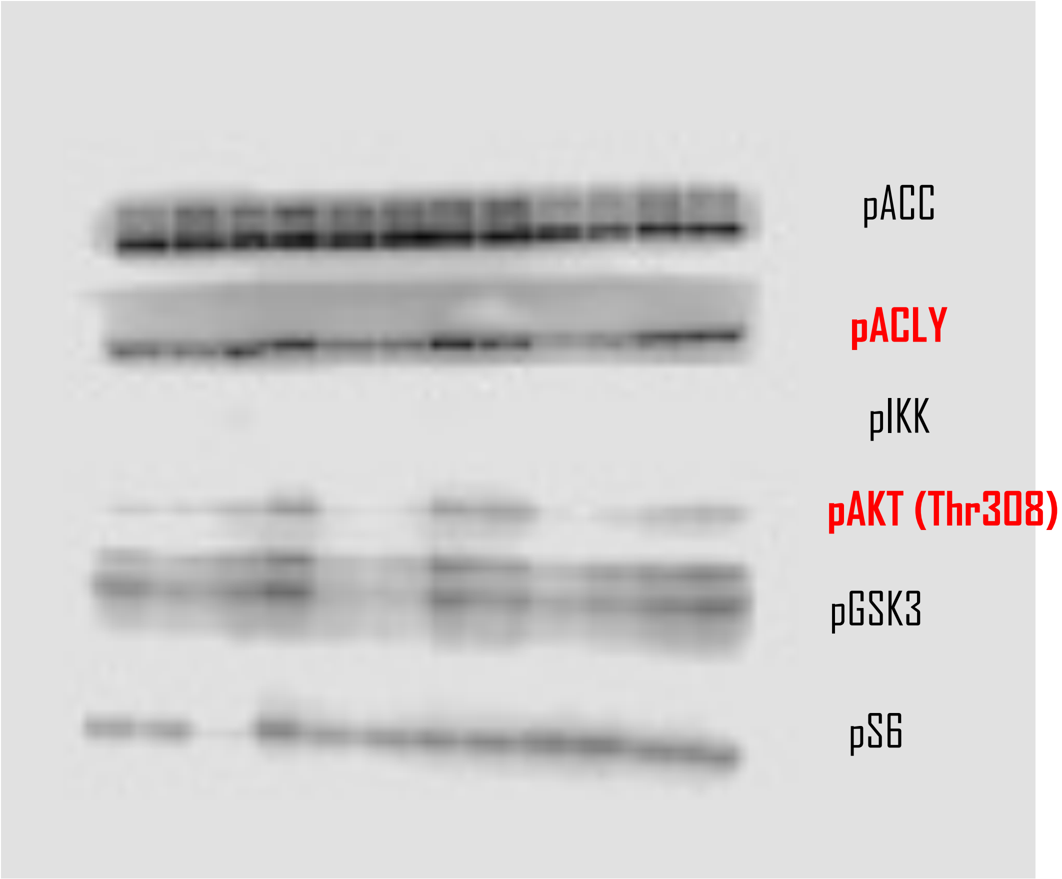

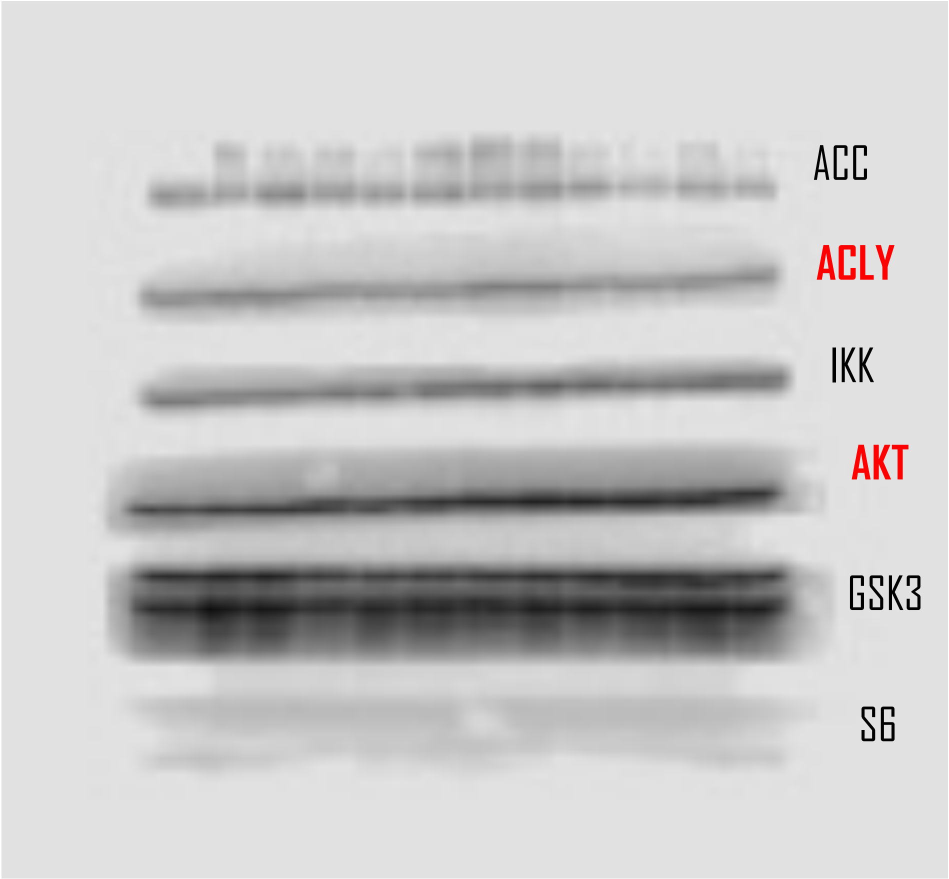

2018

17/04/2018

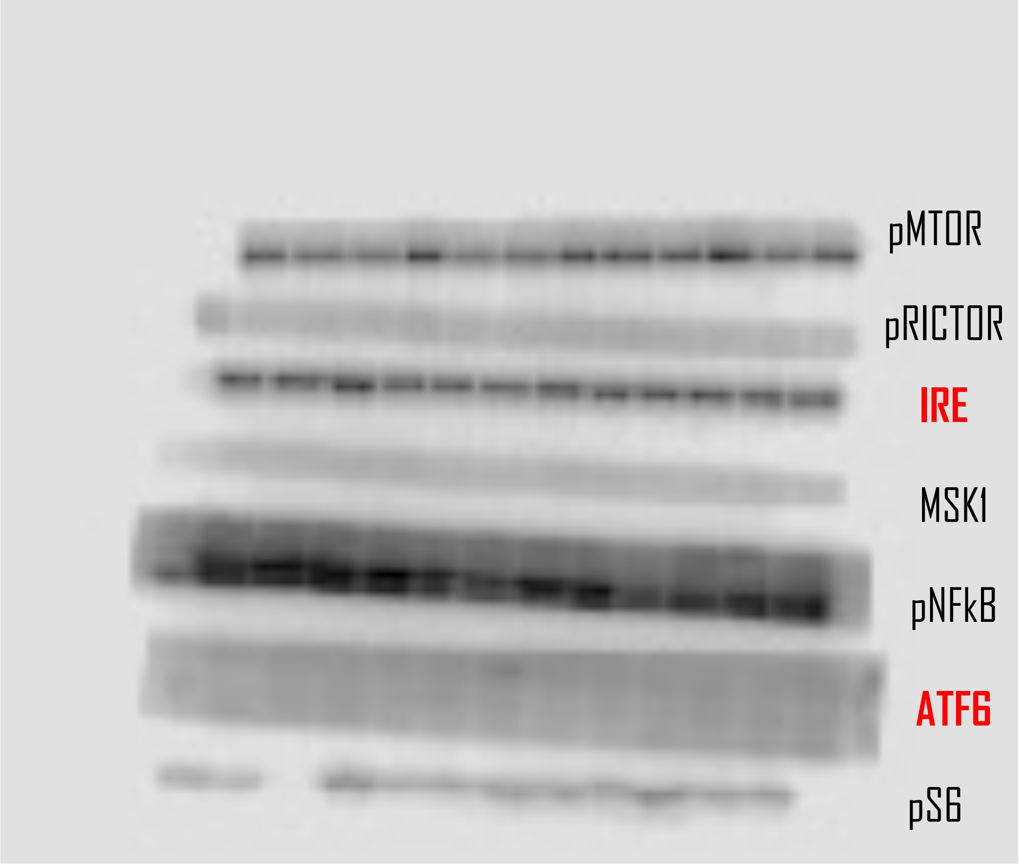

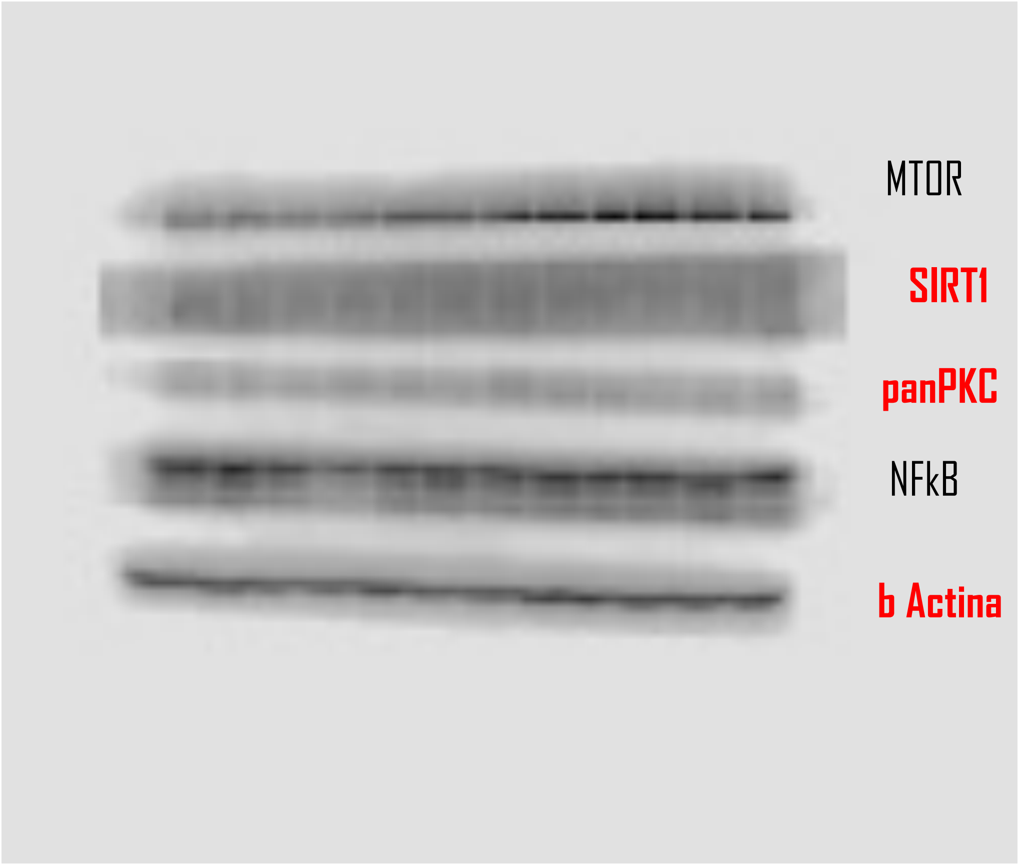

2018

18/04/2018

Gel 1

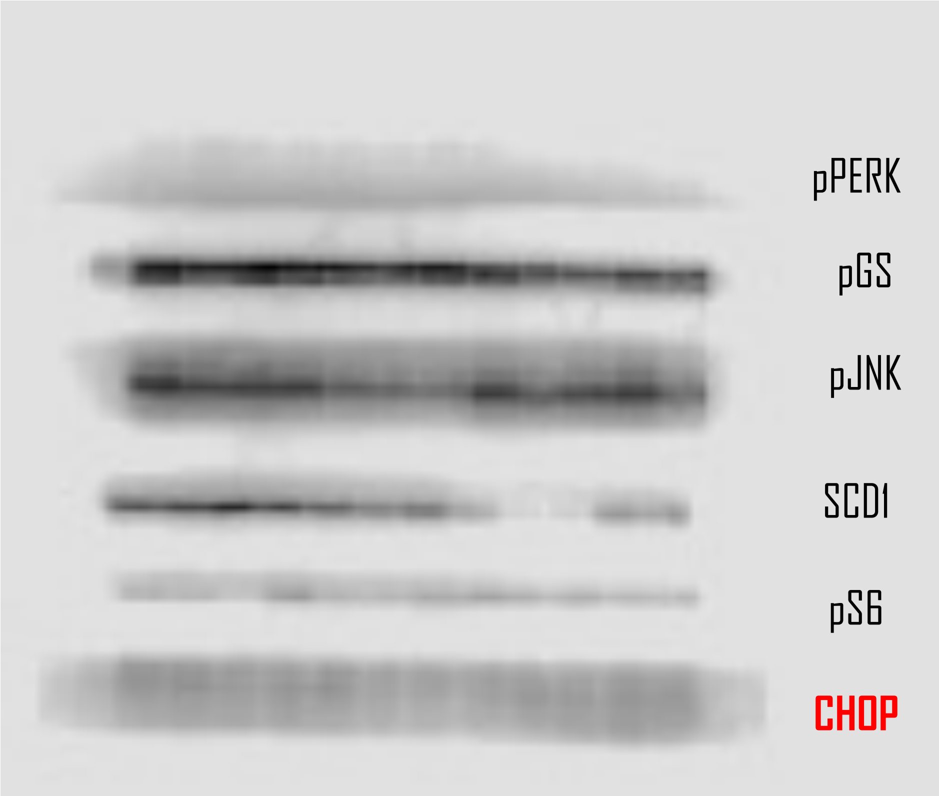

2018

12/06/2018

Gel 1

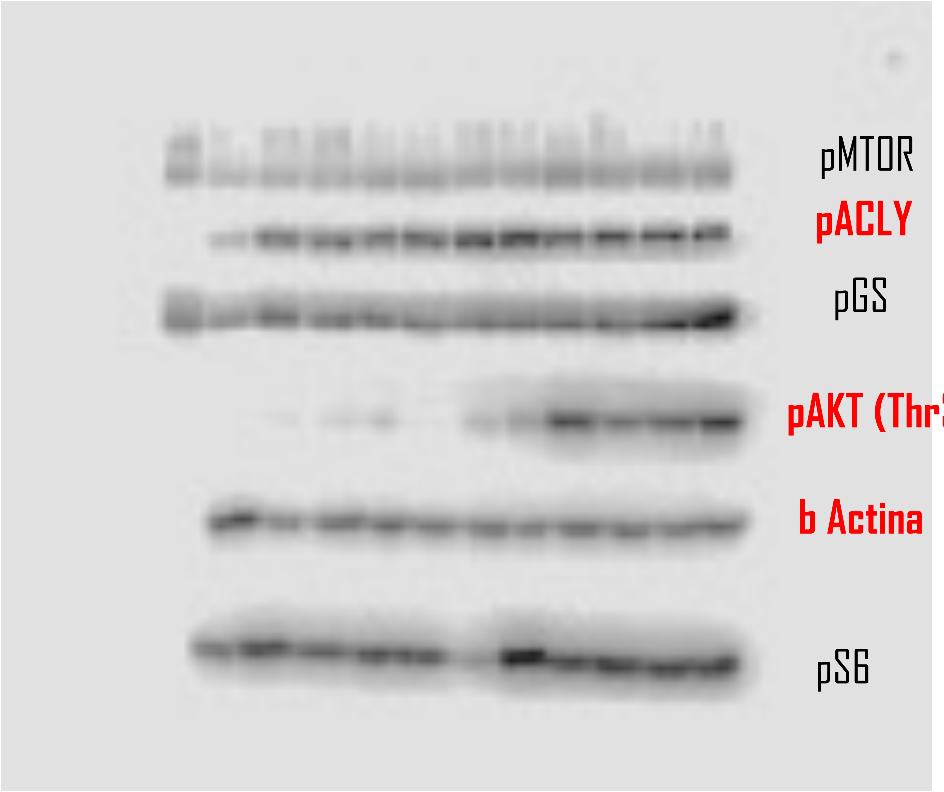

Gel 2

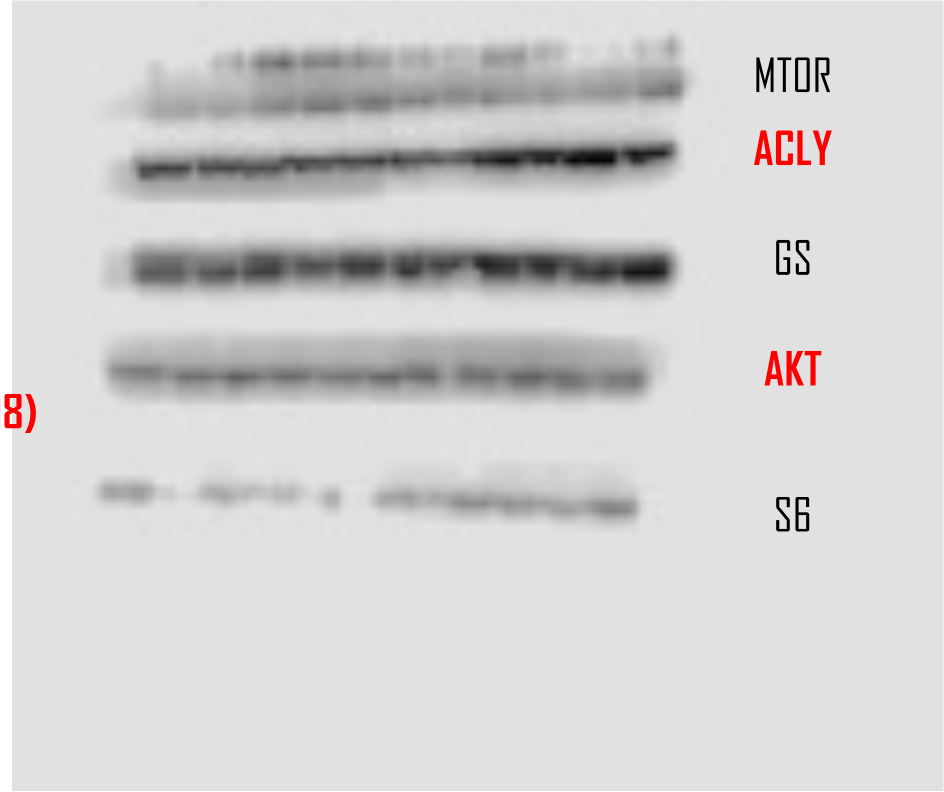

2018

18/06/2018

Gel 1

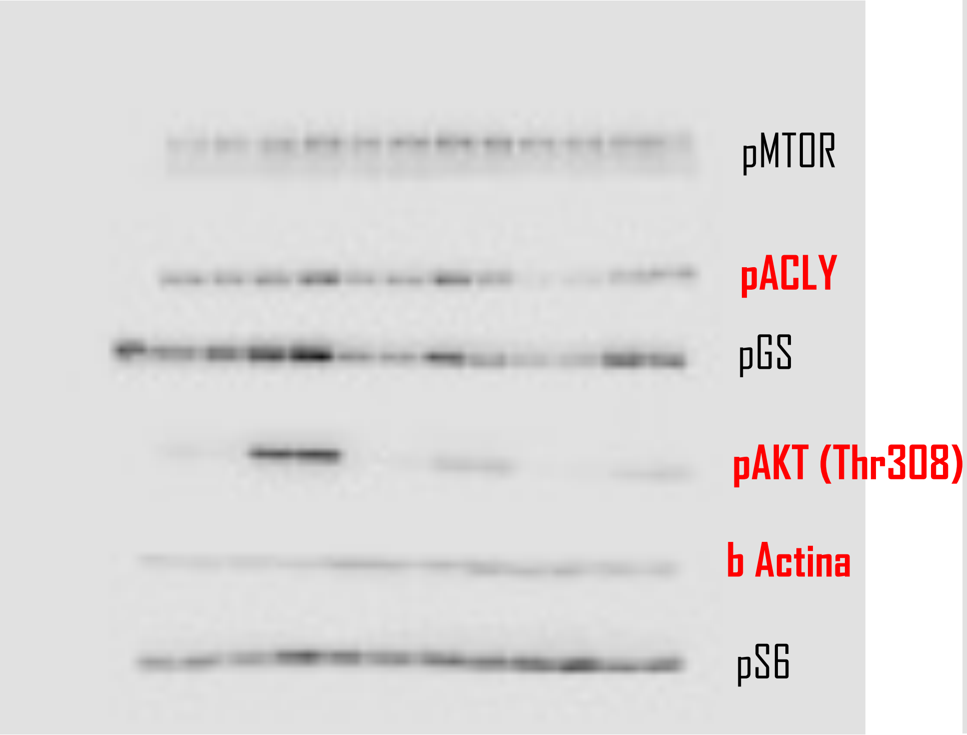

Gel 2

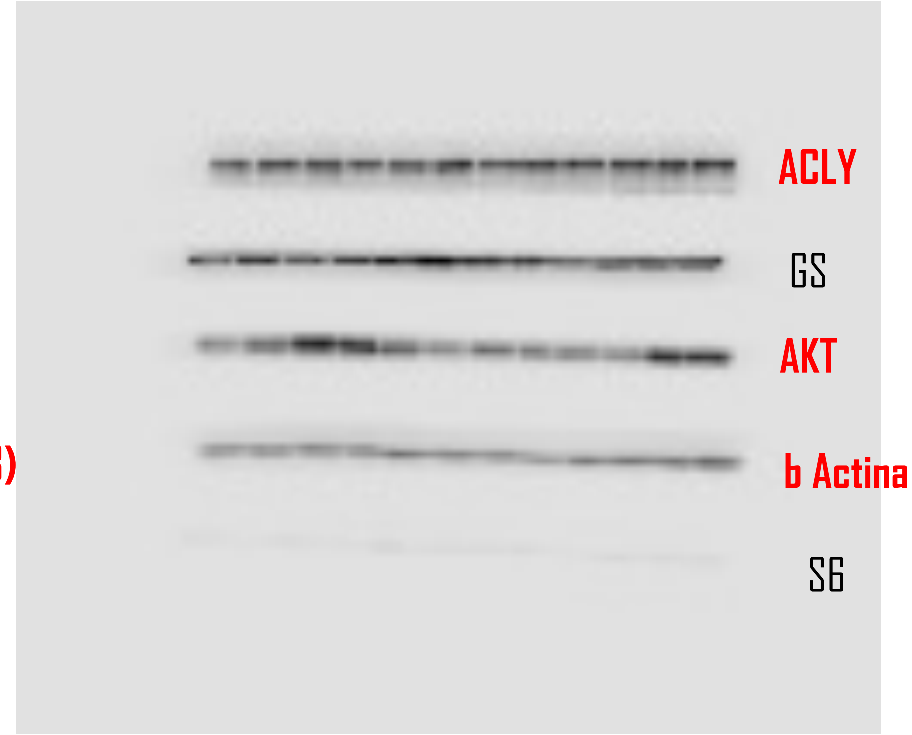

2018

18/06/2018

Gel 3

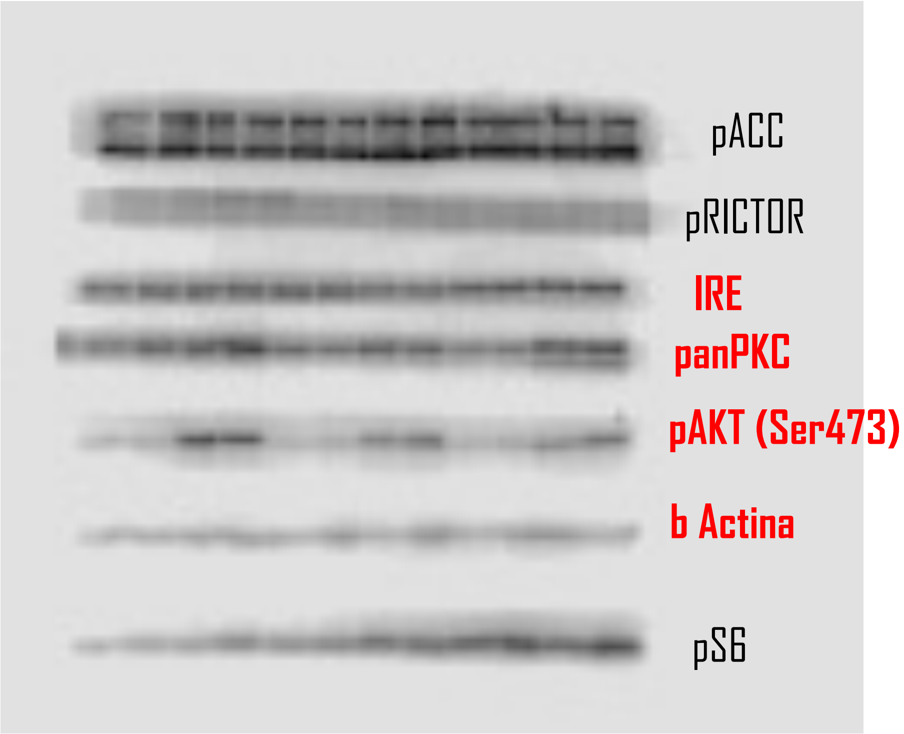

2018

03/09/2018

Gel 1

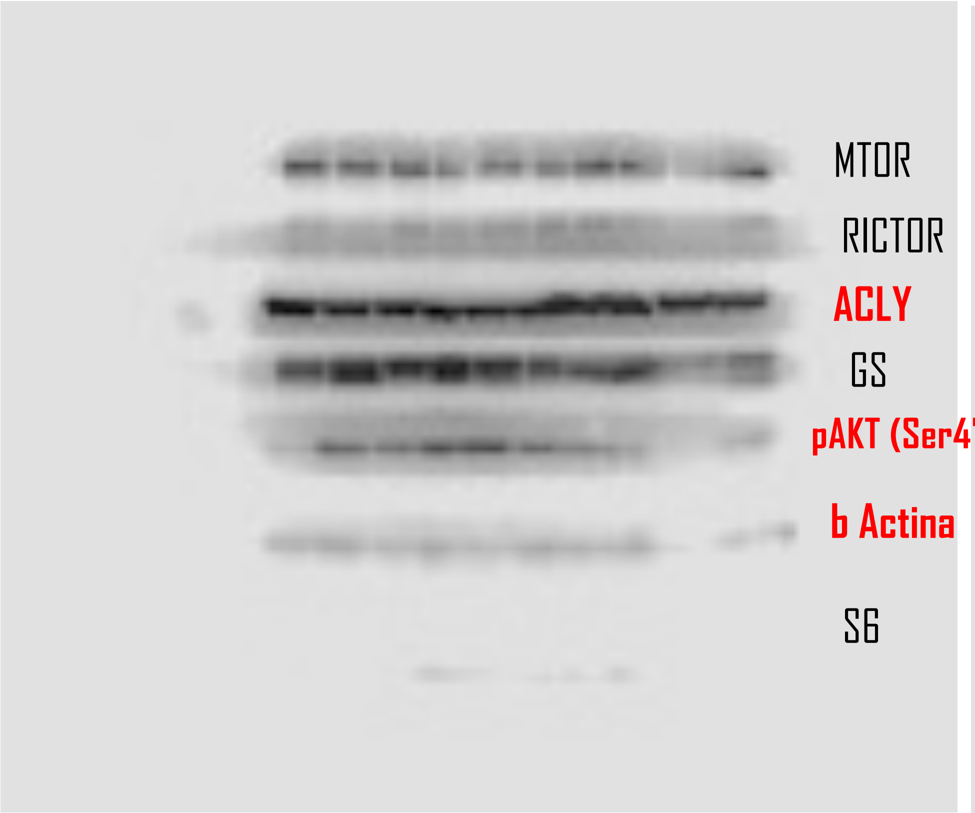

Gel 2

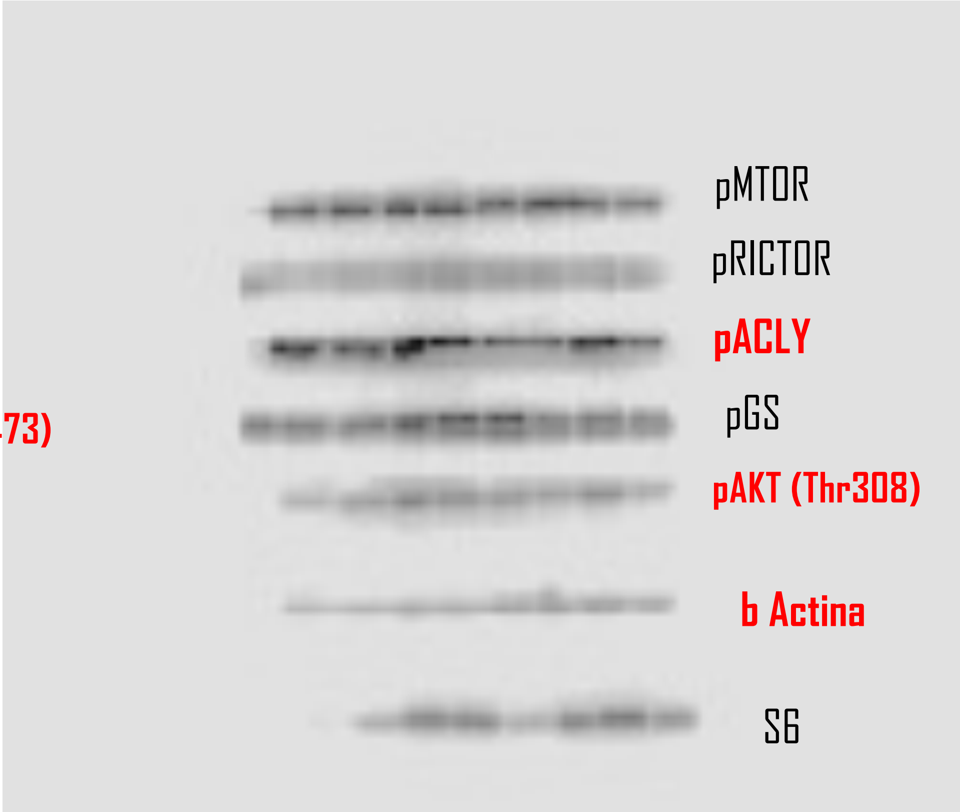

2018

02/10/2018

Gel 1

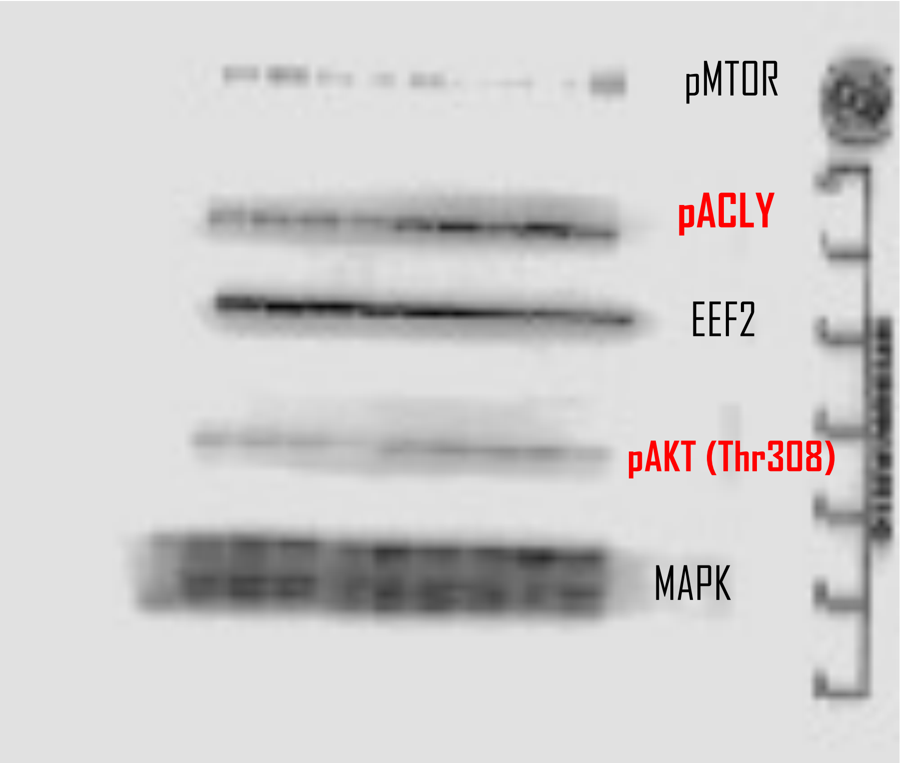

Gel 2

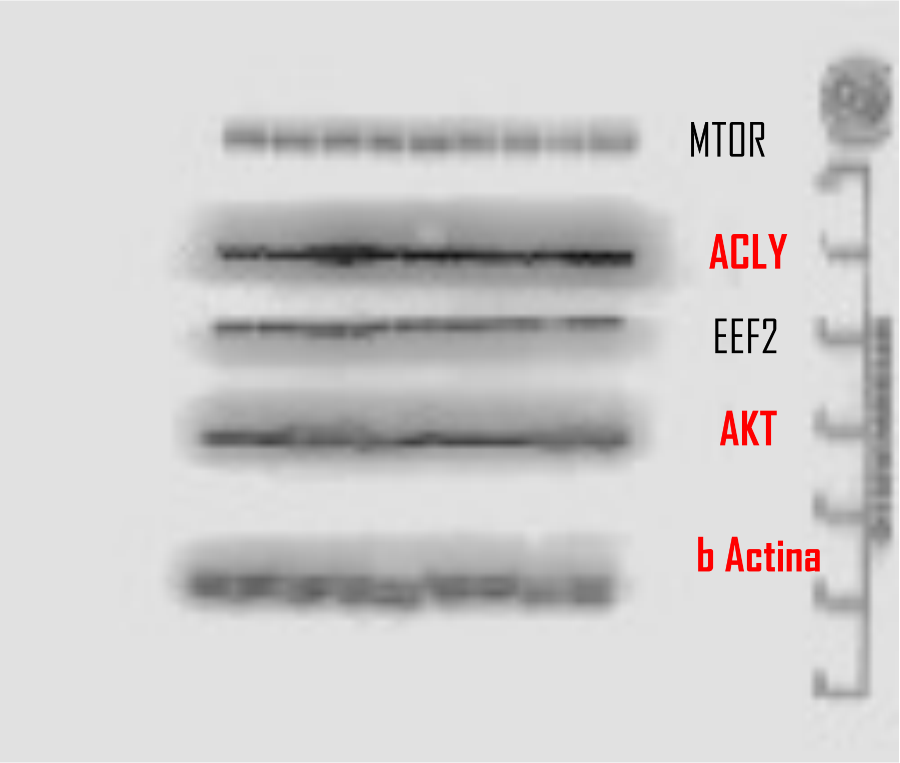

2018

16/10/2018

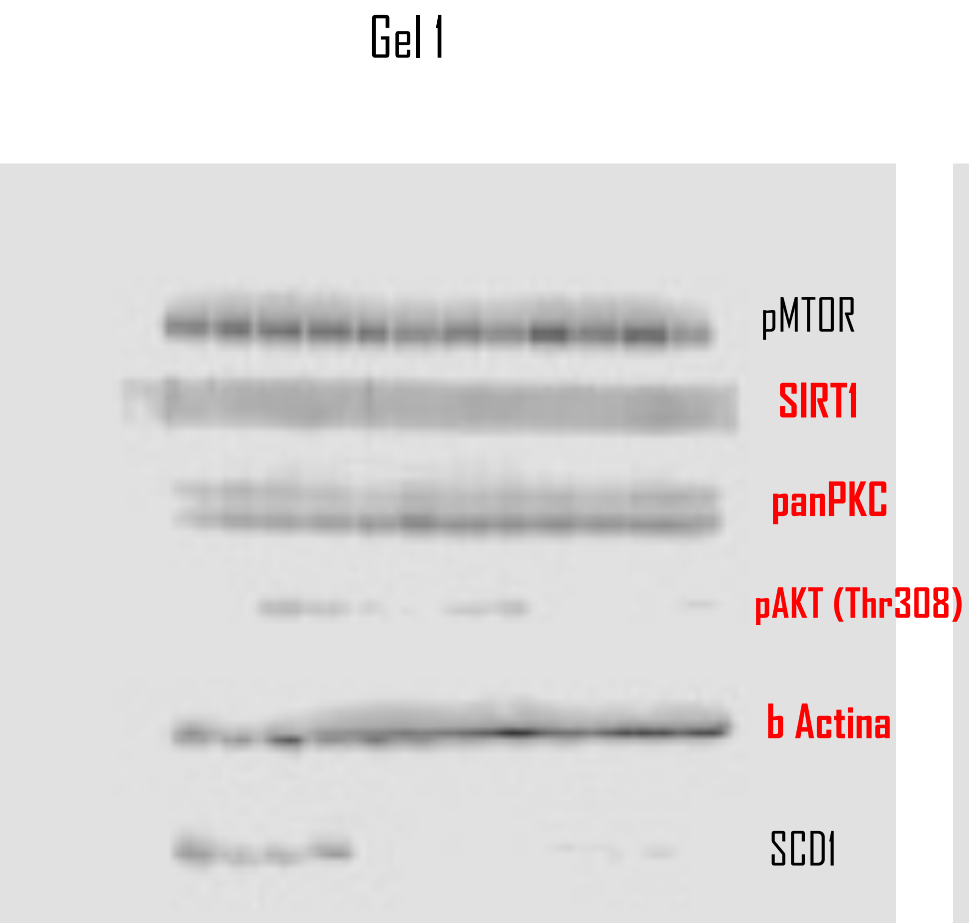

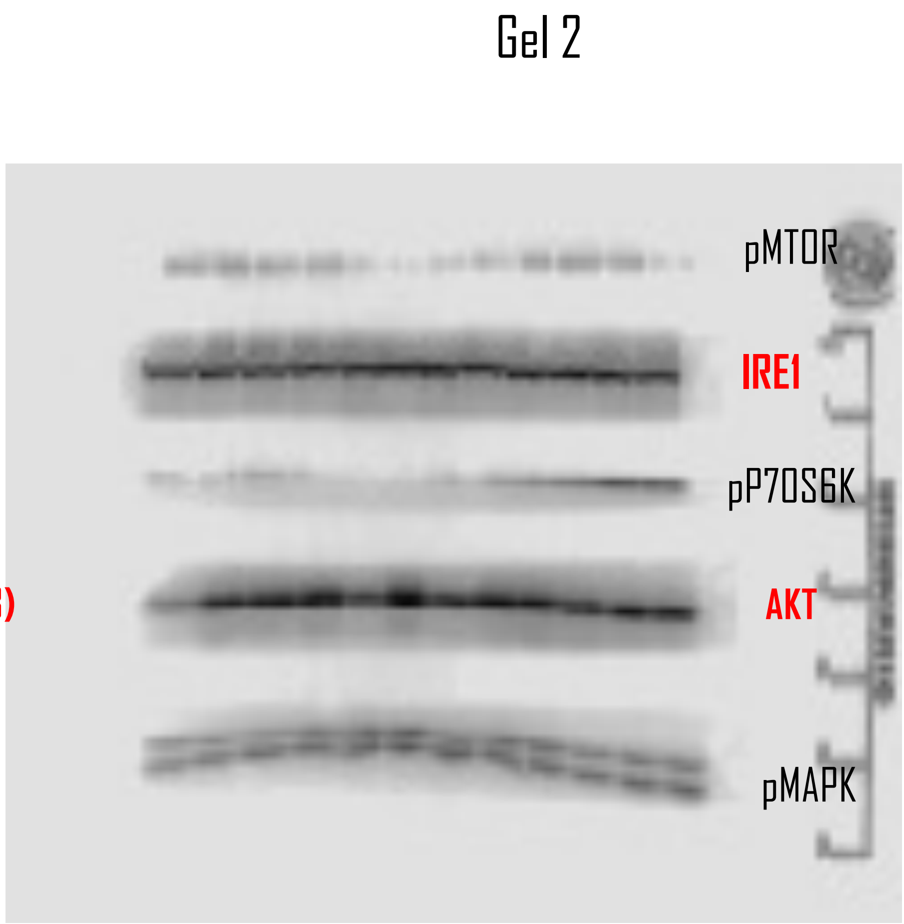

2018

07/11/2018

Gel 1

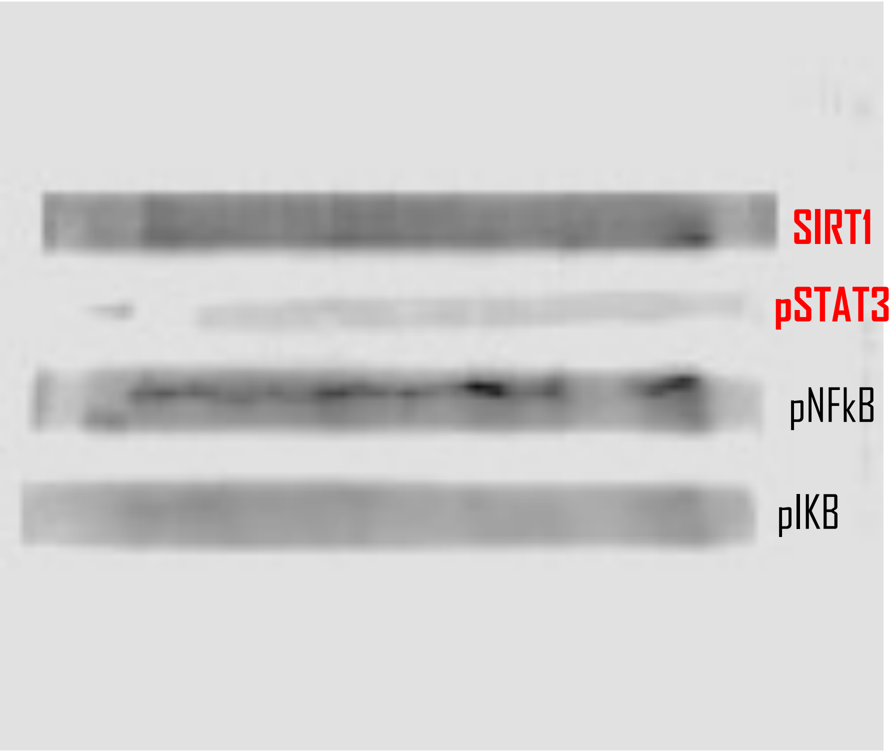

Gel 2

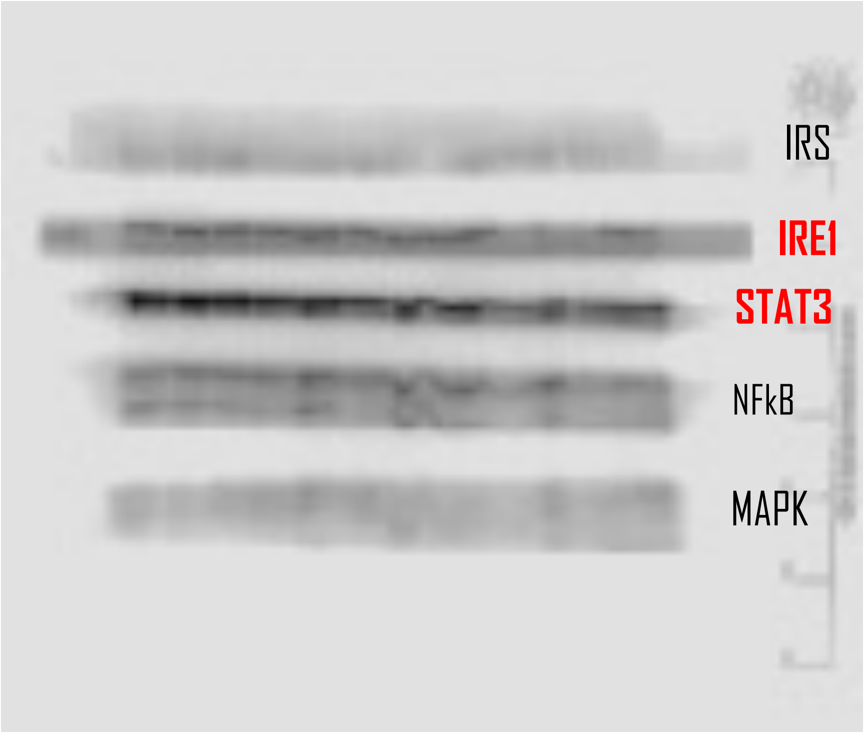

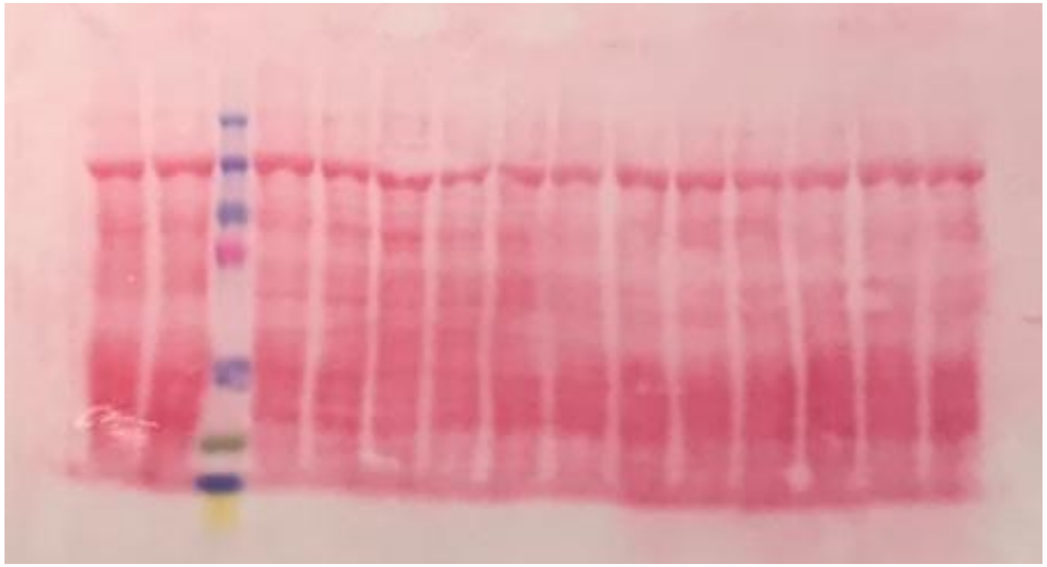

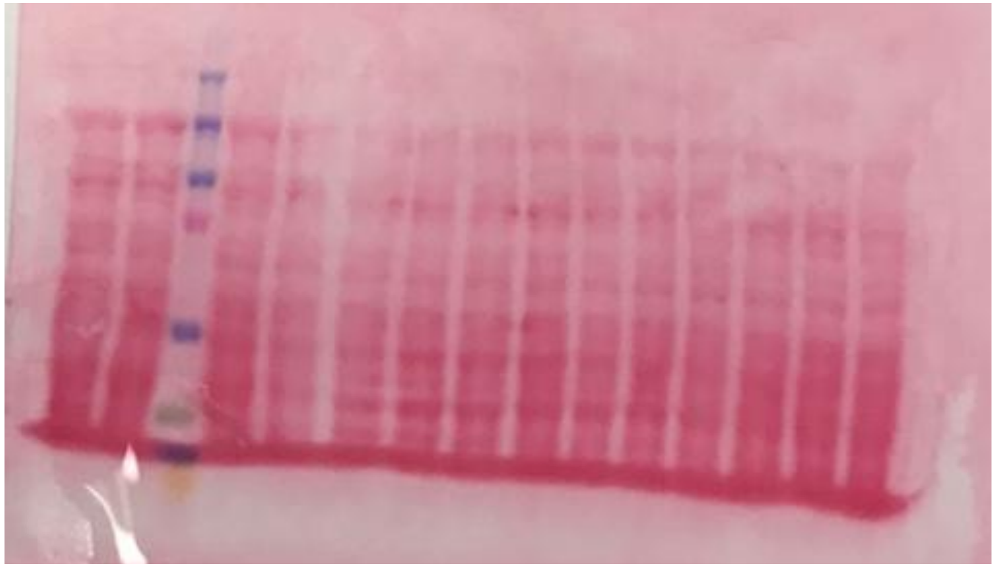

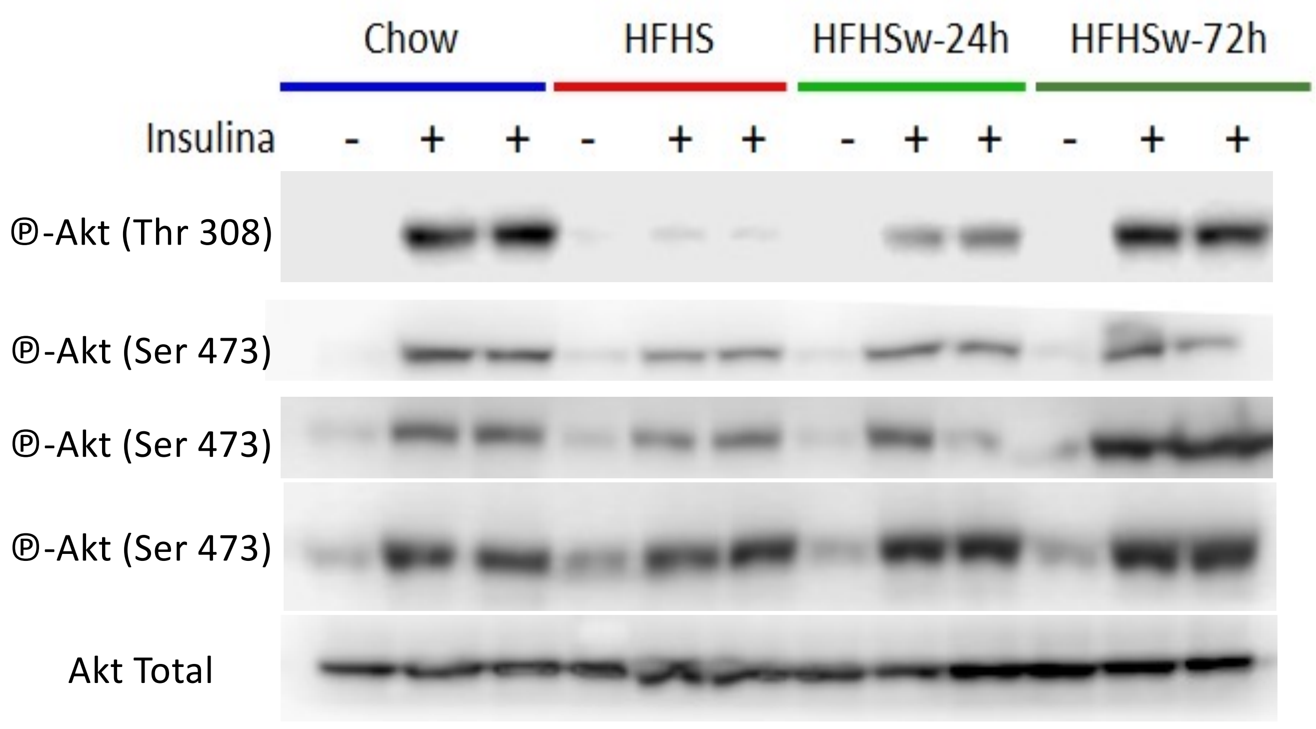

Chow
HFHS
HFHSw-24h
HFHSw-72h
Insulina
- + + - + + - + + - + +
Ⓟ-Akt (Thr 308)
Ⓟ-Akt (Ser 473)
Ⓟ-Akt (Ser 473)
Ⓟ-Akt (Ser 473)
Akt Total

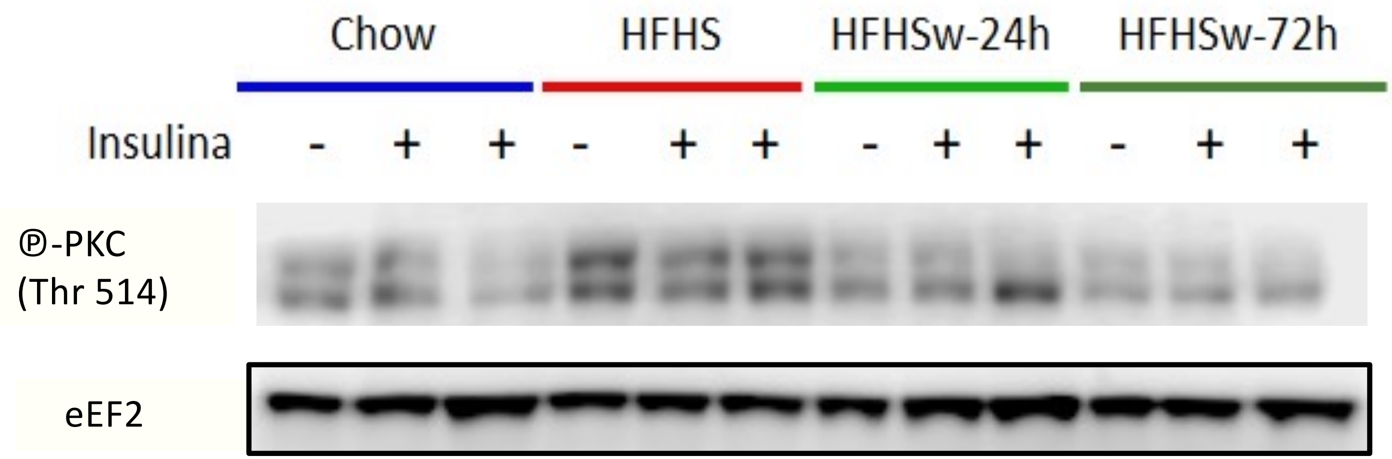
Chow
HFHS
HFHSw-24h
HFHSw-72h
Insulina - + + - + + - + + - + +
℗-PKC
(Thr 514)
eEF2

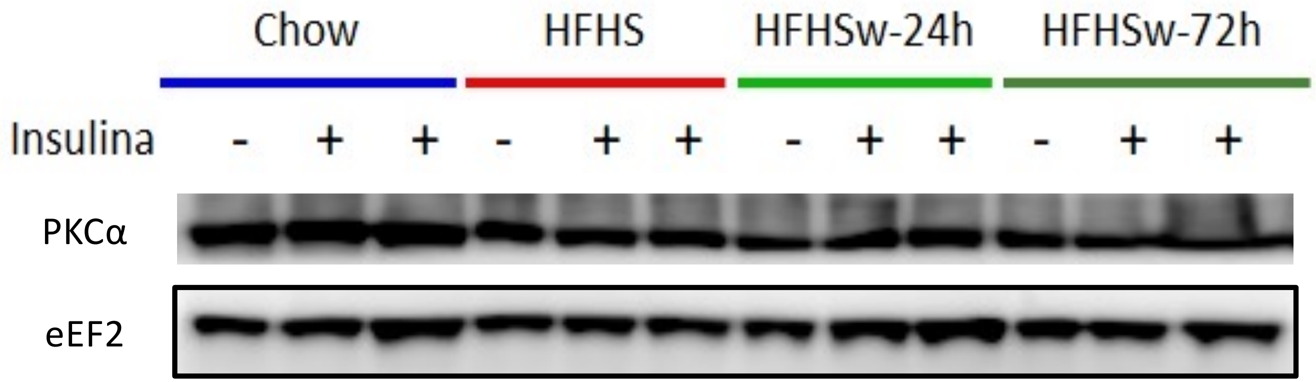
Chow
HFHS
HFHSw-24h
HFHSw-72h
Insulina
- + + - + + - + + - + +
PKCα
eEF2

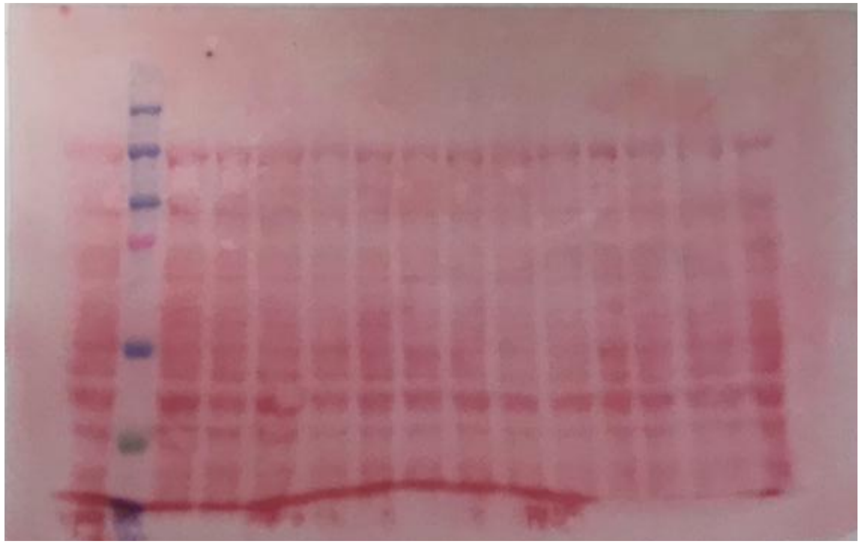
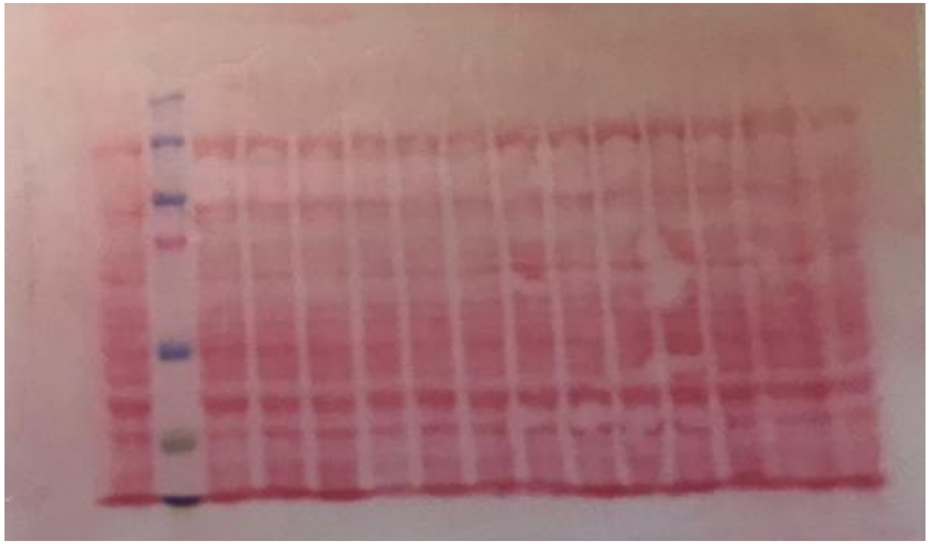

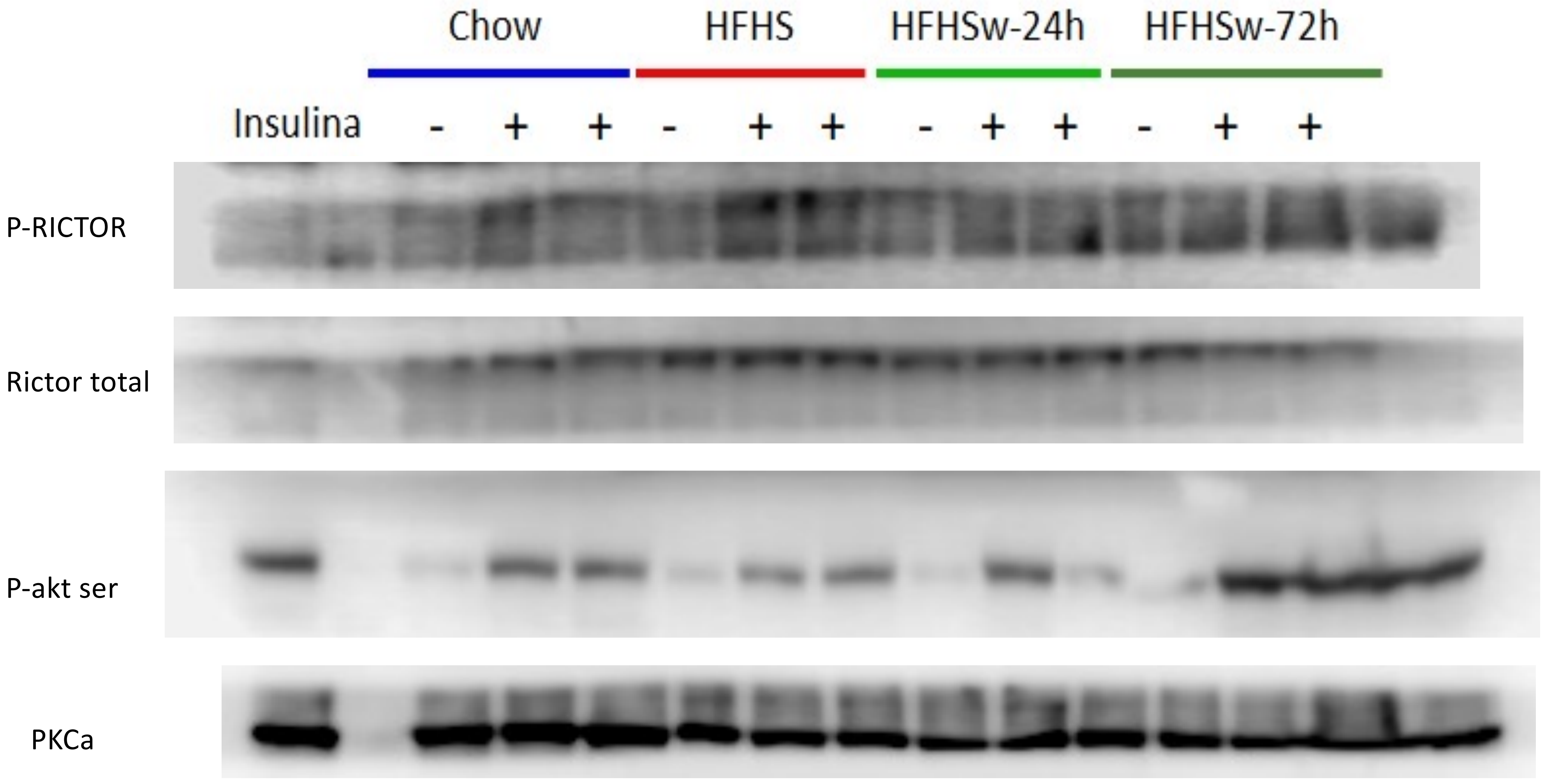
Chow
HFHS
HFHSw-24h
HFHSw-72h
Insulina
- + + - + + - + + - + +
P-RICTOR
Rictor total
P-akt ser
PKCa

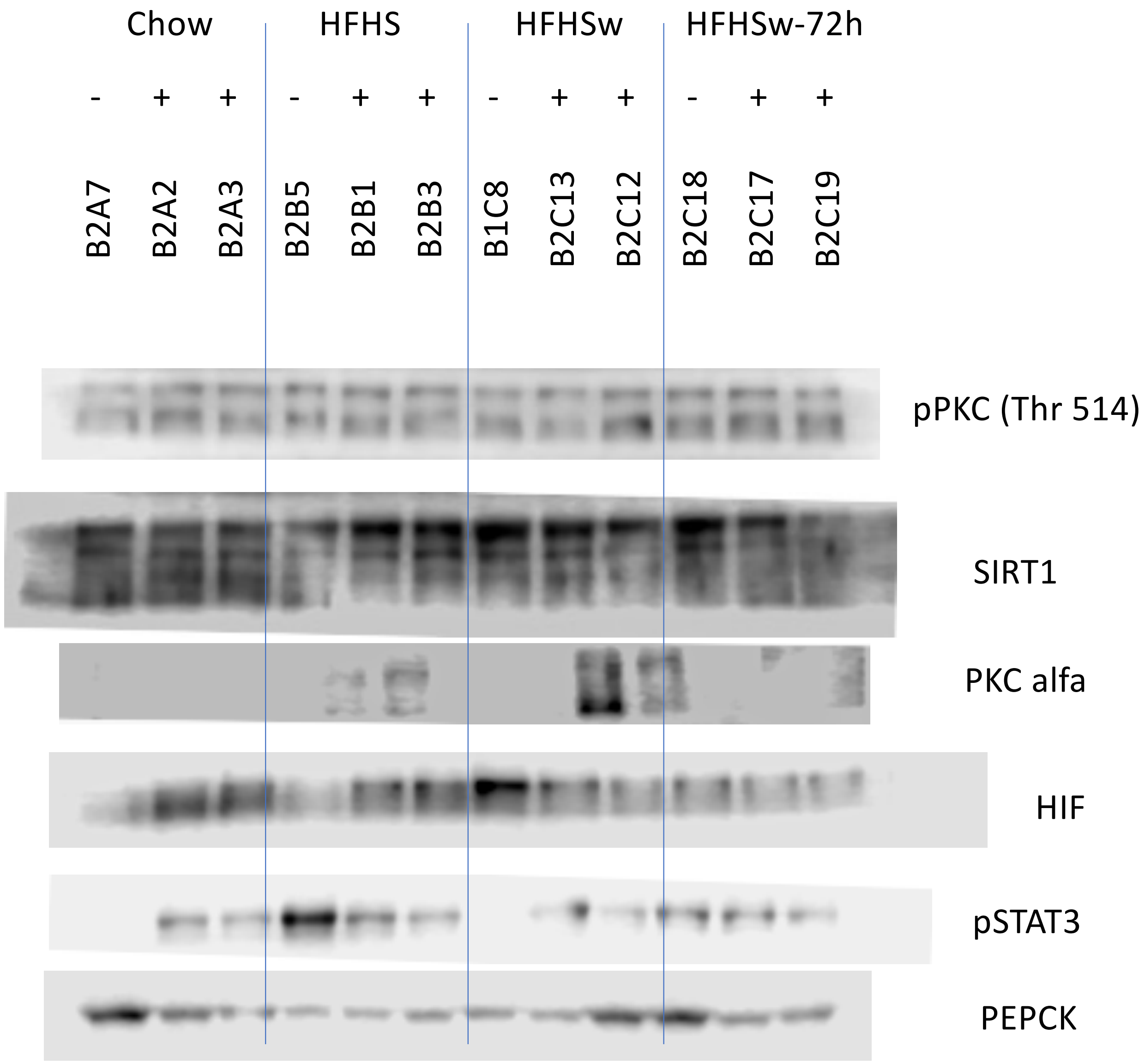
Chow
HFHS
HFHSw
HFHSw-72h
- + + - + + - + + - + +
B2A7
B2A2
B2A3
B2B5
B2B1
B2B3
B1C8
B2C13
B2C12
B2C18
B2C17
B2C19
pPKC (Thr 514)
SIRT1
PKC alfa
HIF
pSTAT3
PEPCK

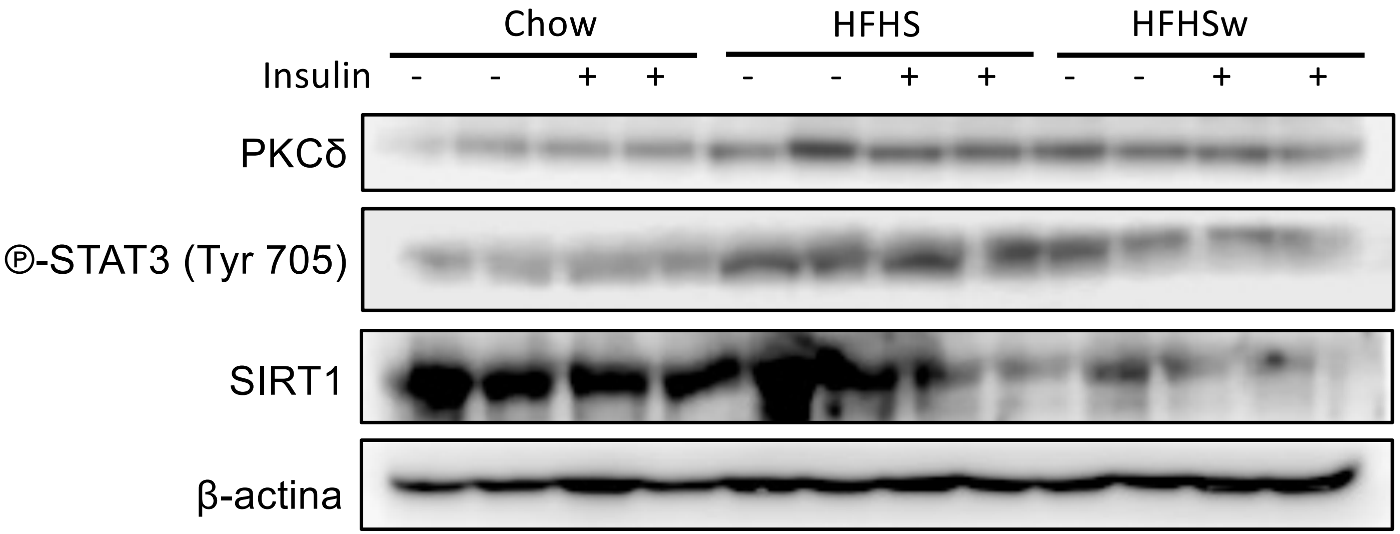
Chow
HFHS
HFHSw
Insulin - - + + - - + + - - + +
PKCδ
℗-STAT3 (Tyr 705)
SIRT1
β-actina

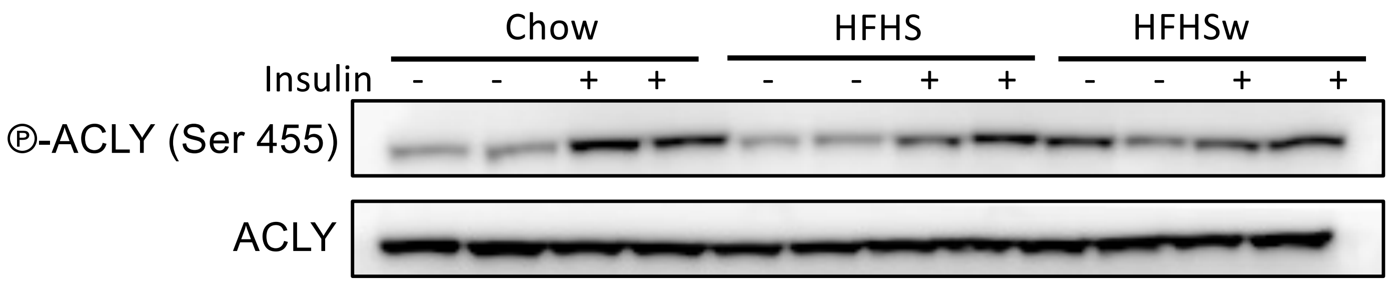
Chow
HFHS
HFHSw
Insulin - - + + - - + + - - + +
℗-ACLY (Ser 455)
ACLY

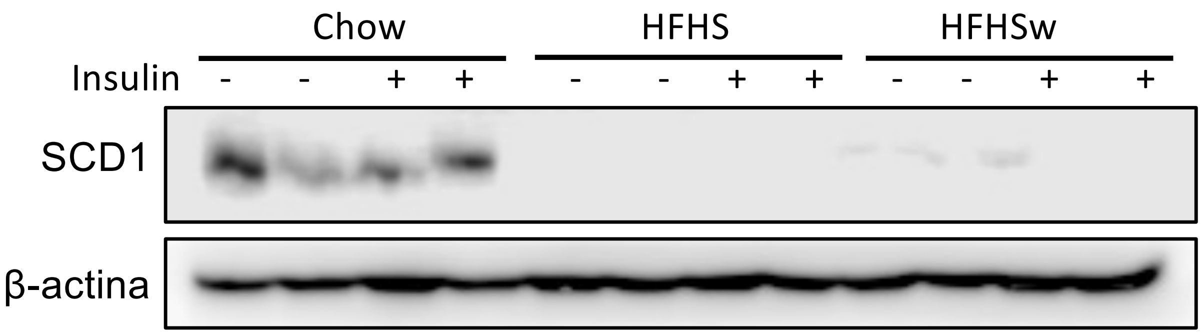
Chow
HFHS
HFHSw
Insulin - - + + - - + + - - + +
SCD1
β-actina

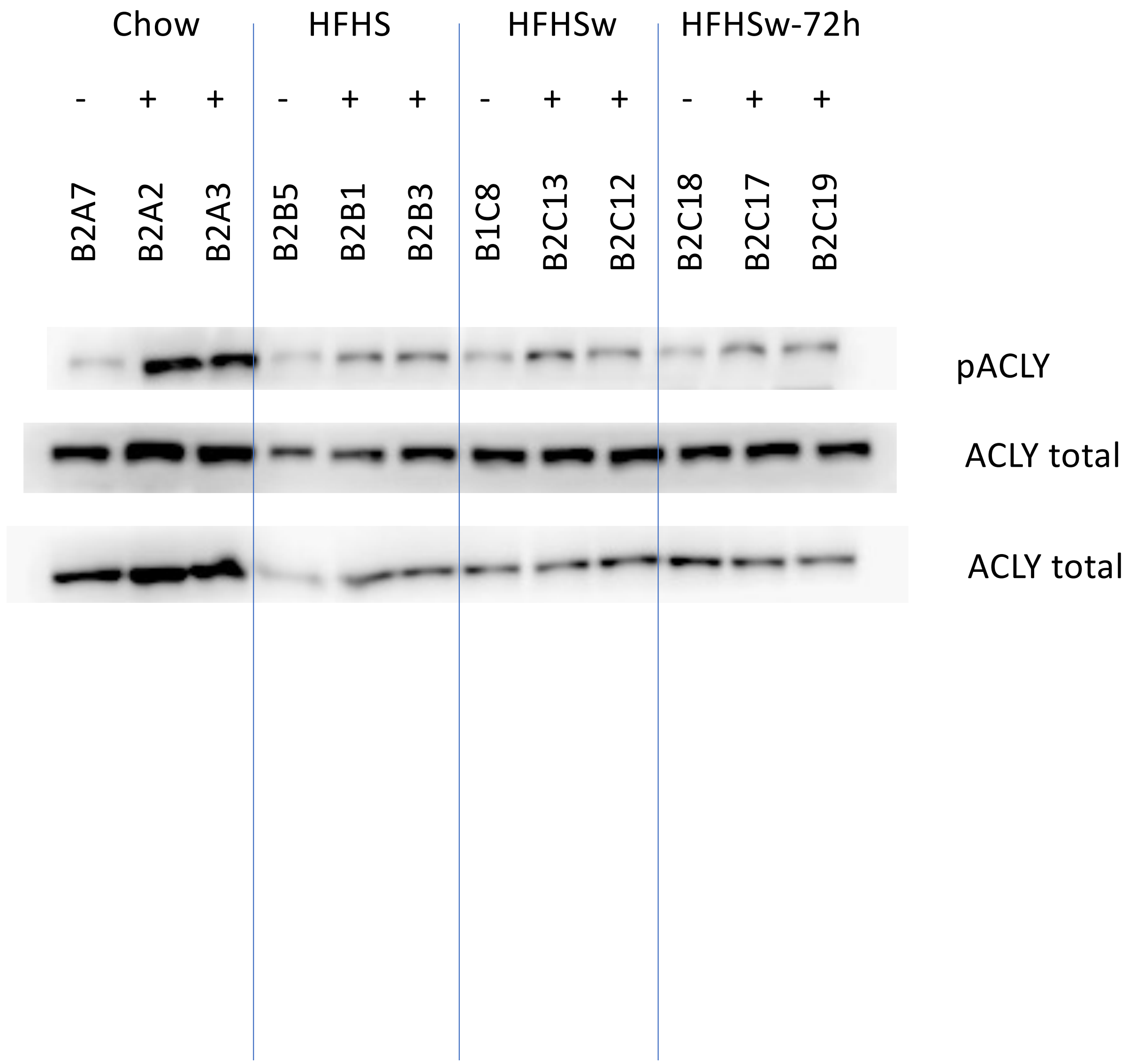
Chow
HFHS
HFHSw
HFHSw-72h
- + + - + + - + + - + +
B2A7
B2A2
B2A3
B2B5
B2B1
B2B3
B1C8
B2C13
B2C12
B2C18
B2C17
B2C19
pACLY
ACLY total
ACLY total

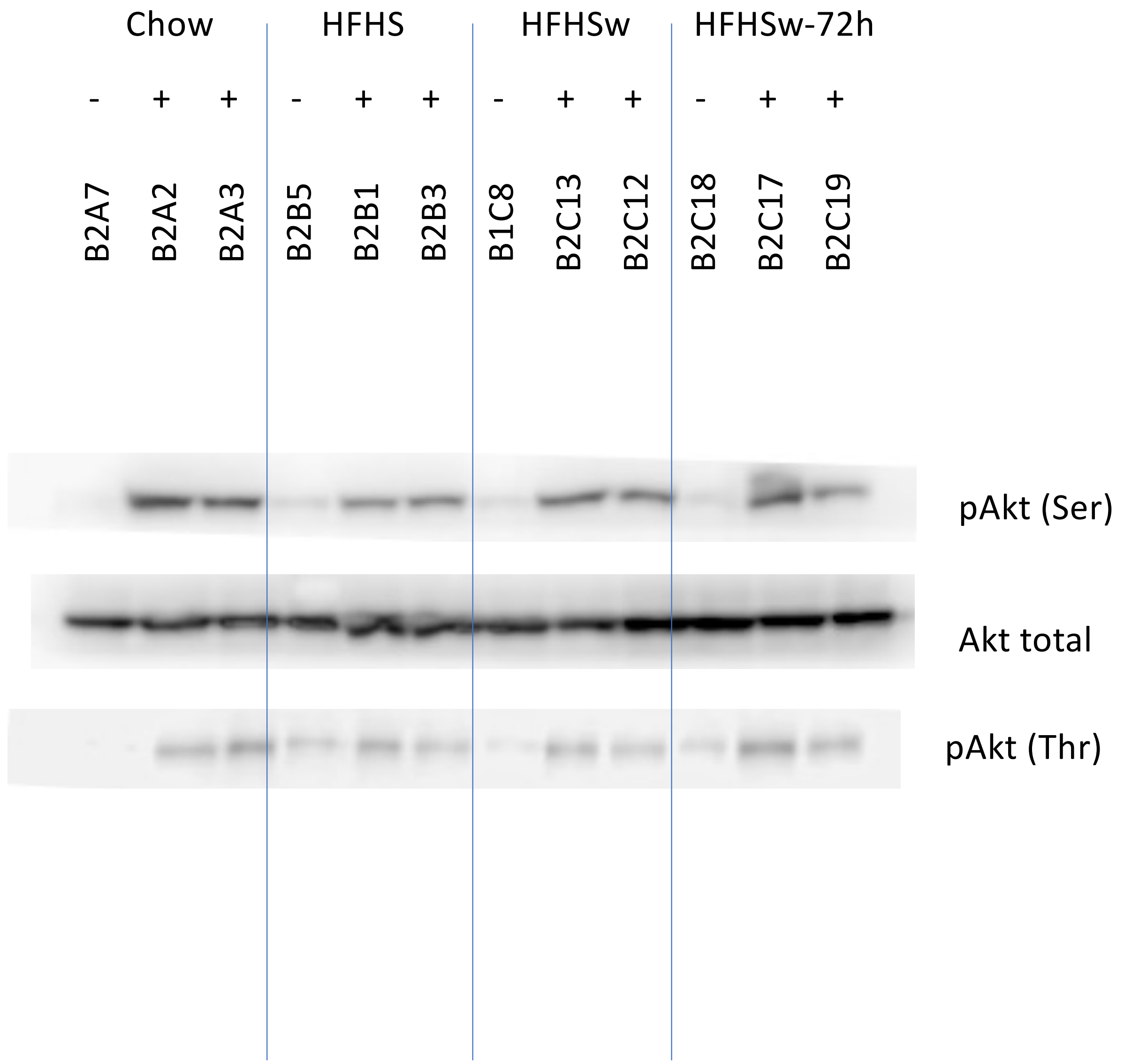

Chow
HFHS
HFHSw
HFHSw-72h
- + + - + + - + + - + +
B2A7
B2A2
B2A3
B2B5
B2B1
B2B3
B1C8
B2C13
B2C12
B2C18
B2C17
B2C19
pAkt (Ser)
Akt total
pAkt (Thr)

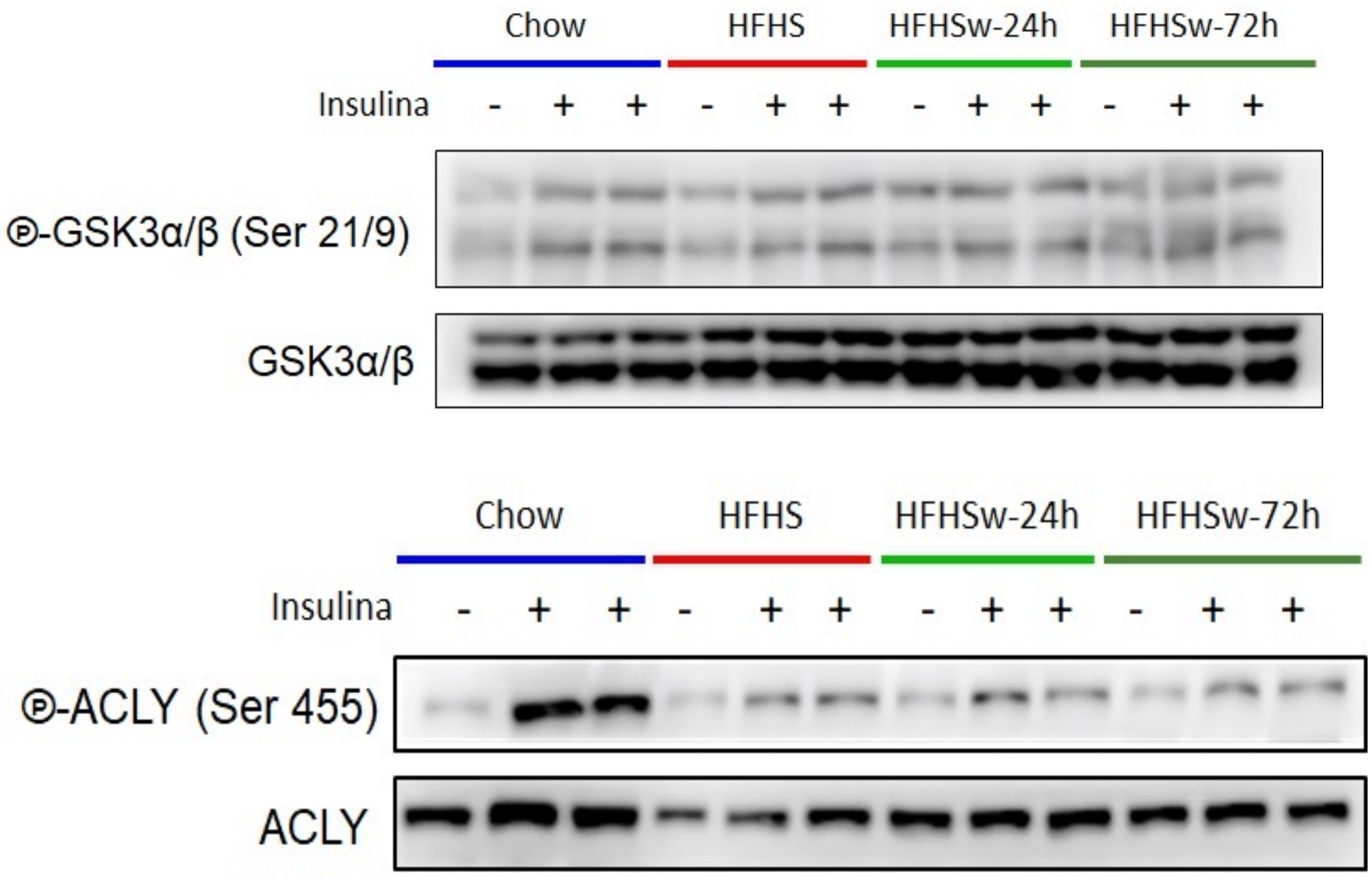
Chow
HFHS
HFHSw-24h
HFHSw-72h
Insulina
- + + - + + - + + - + +
Ⓟ-GSK3α/β (Ser 21/9)
GSK3α/β
Chow
HFHS
HFHSw-24h
HFHSw-72h
Insulina
- + + - + + - + + - + +
Ⓟ-ACLY (Ser 455)
ACLY

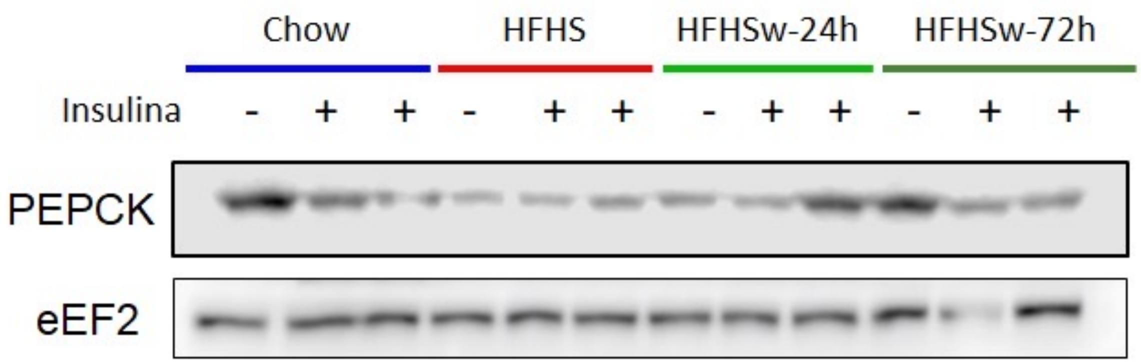
Chow
HFHS
HFHSw-24h
HFHSw-72h
Insulina - + + - + + - + + - + +
PEPCK
eEF2

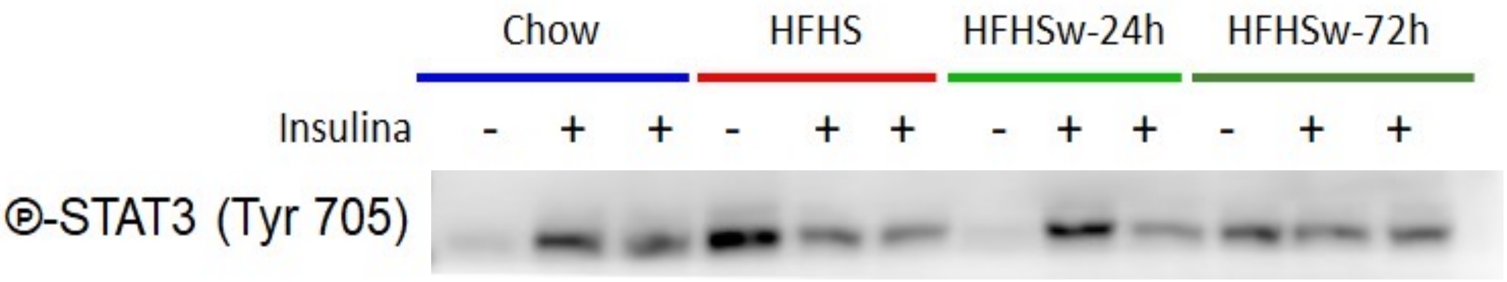
Chow
HFHS
HFHSw-24h
HFHSw-72h
Insulina - + + - + + - + + - + +
Ⓟ-STAT3 (Tyr 705)

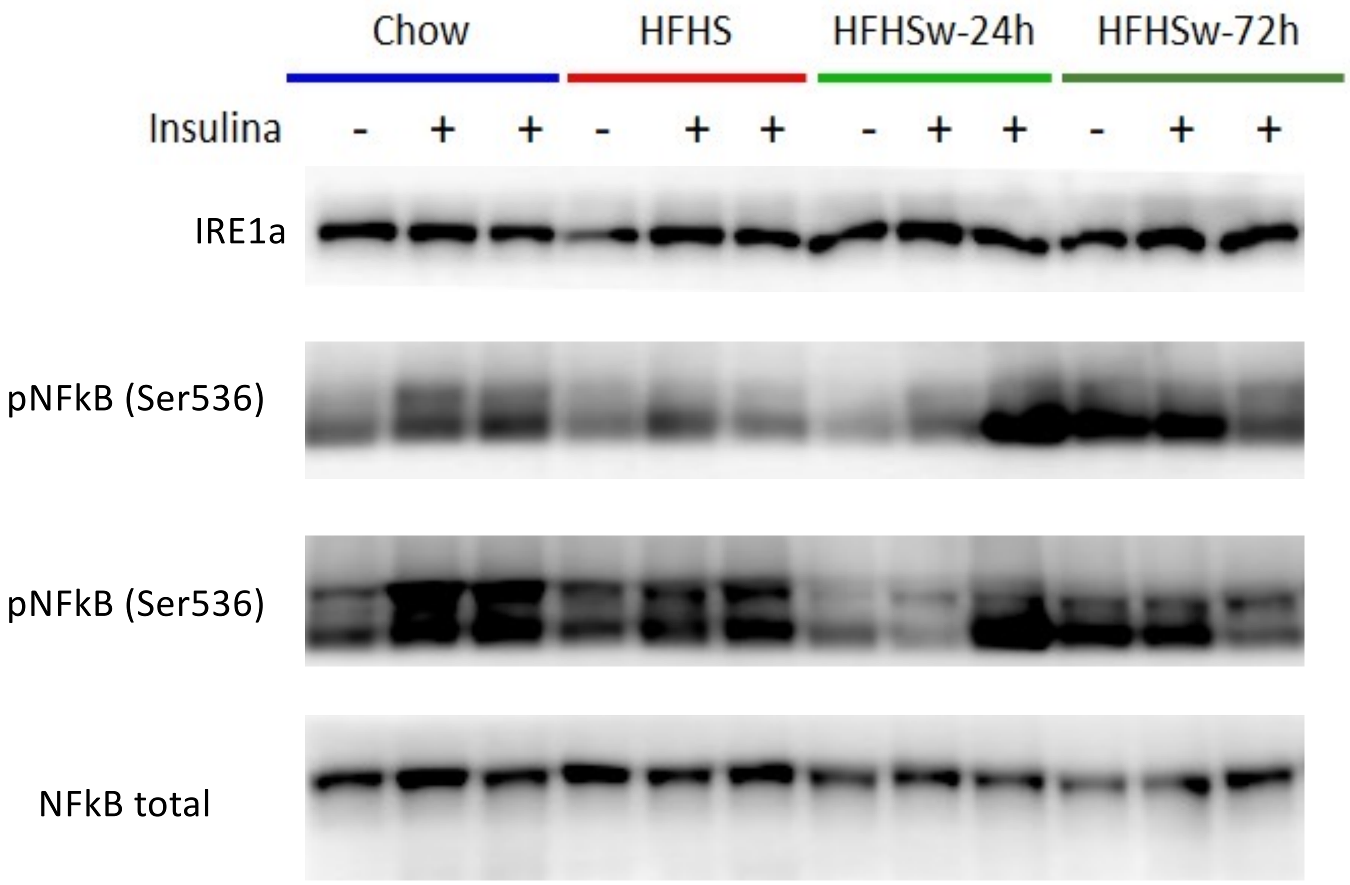

Chow
HFHS
HFHSw-24h
HFHSw-72h
Insulina - + + - + + - + + - + +
IRE1a
pNFkB (Ser536)
pNFkB (Ser536)
NFkB total

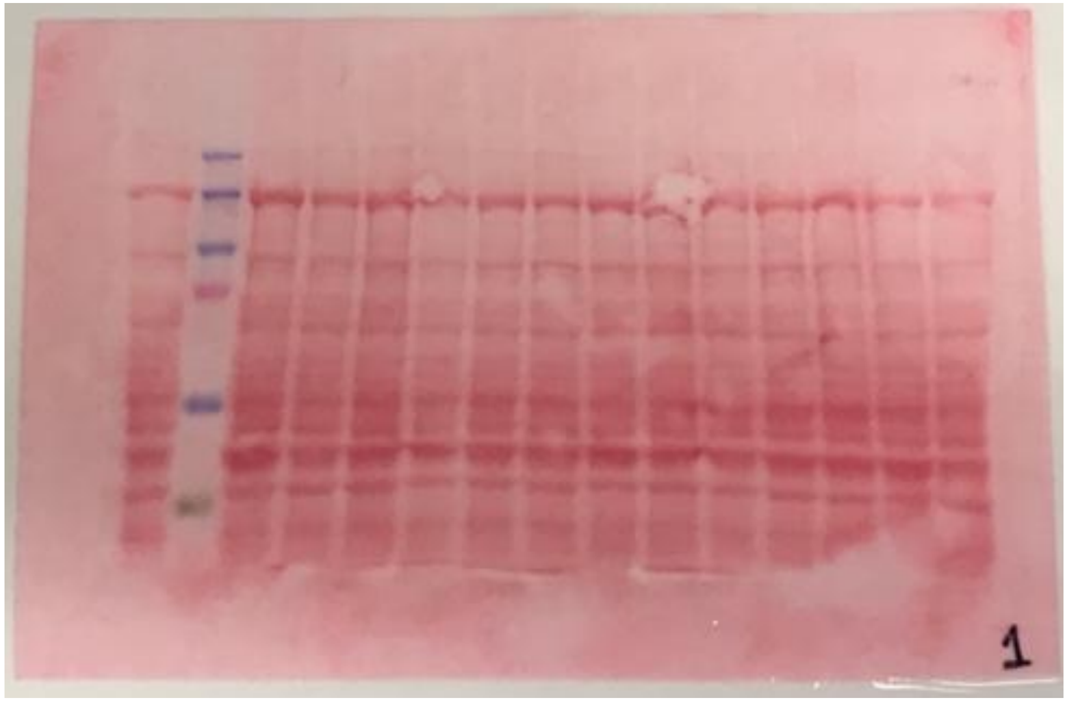
1

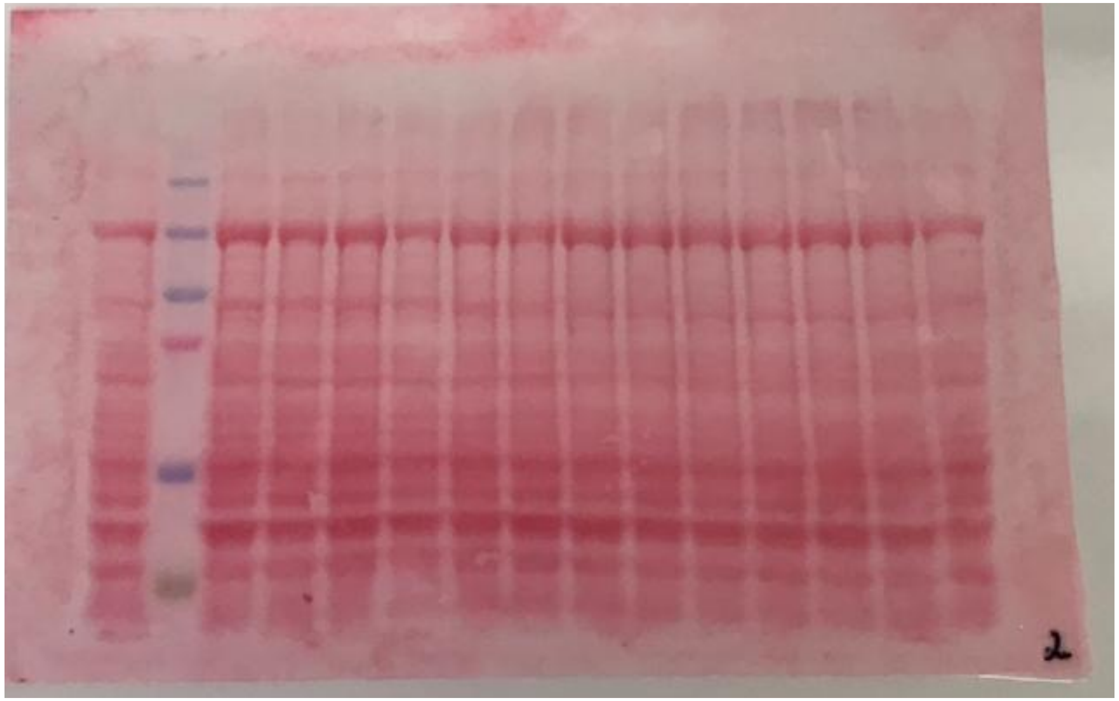
2

Gel 1

- Amostra Chow salina B2A7 → B1A14
- Chow insulina B2A2 e B2A3 → B2A6 e B2A9
- Amostras das pontas: B2A6 (Chow insulina)

B2A9 nunca marcou pmTOR

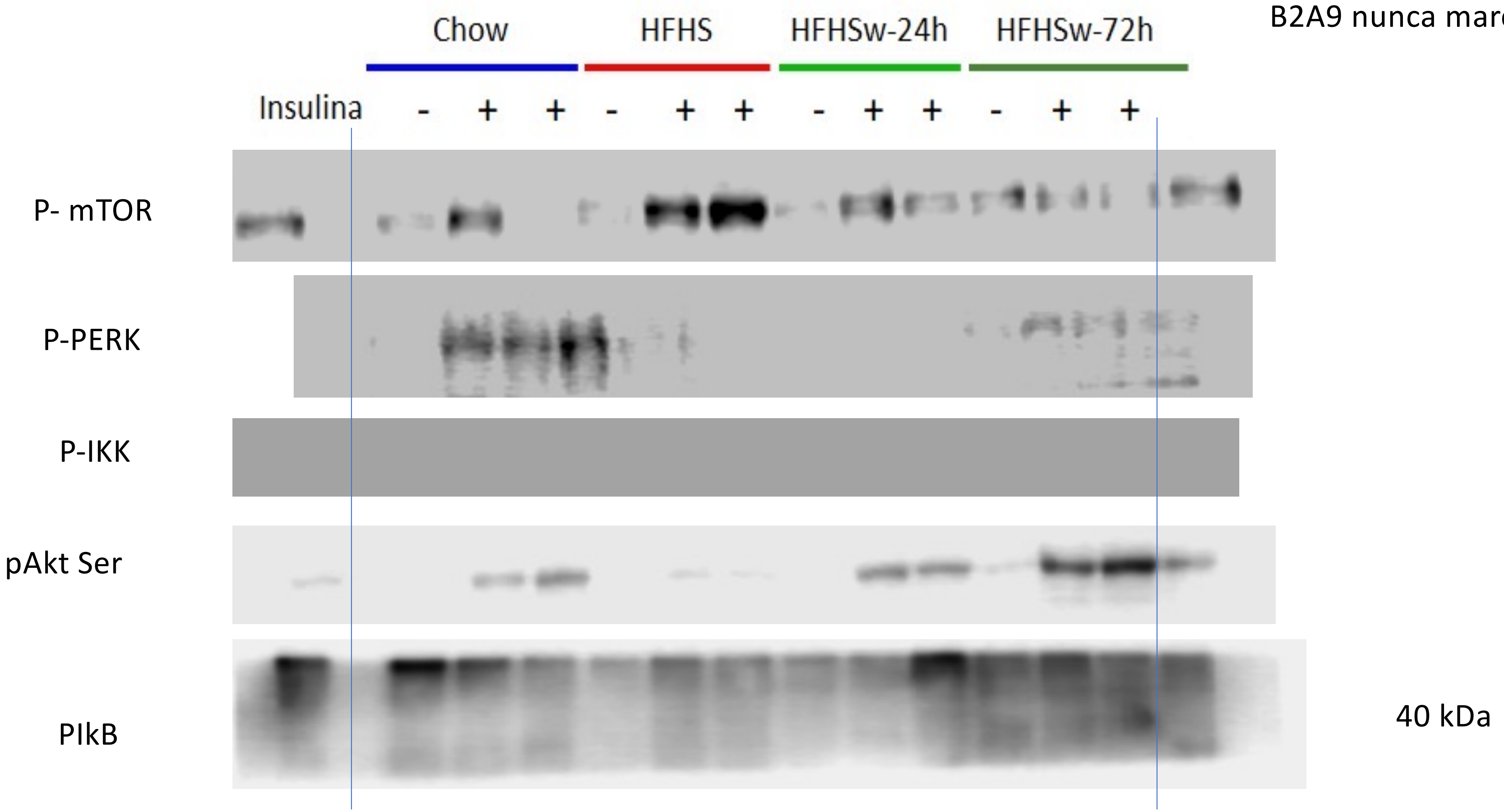

- Amostra Chow salina B2A7 → B1A14
- Chow insulina B2A2 e B2A3 → B2A6 e B2A9
- Amostras das pontas: B2A6 (Chow insulina)

Gel 2

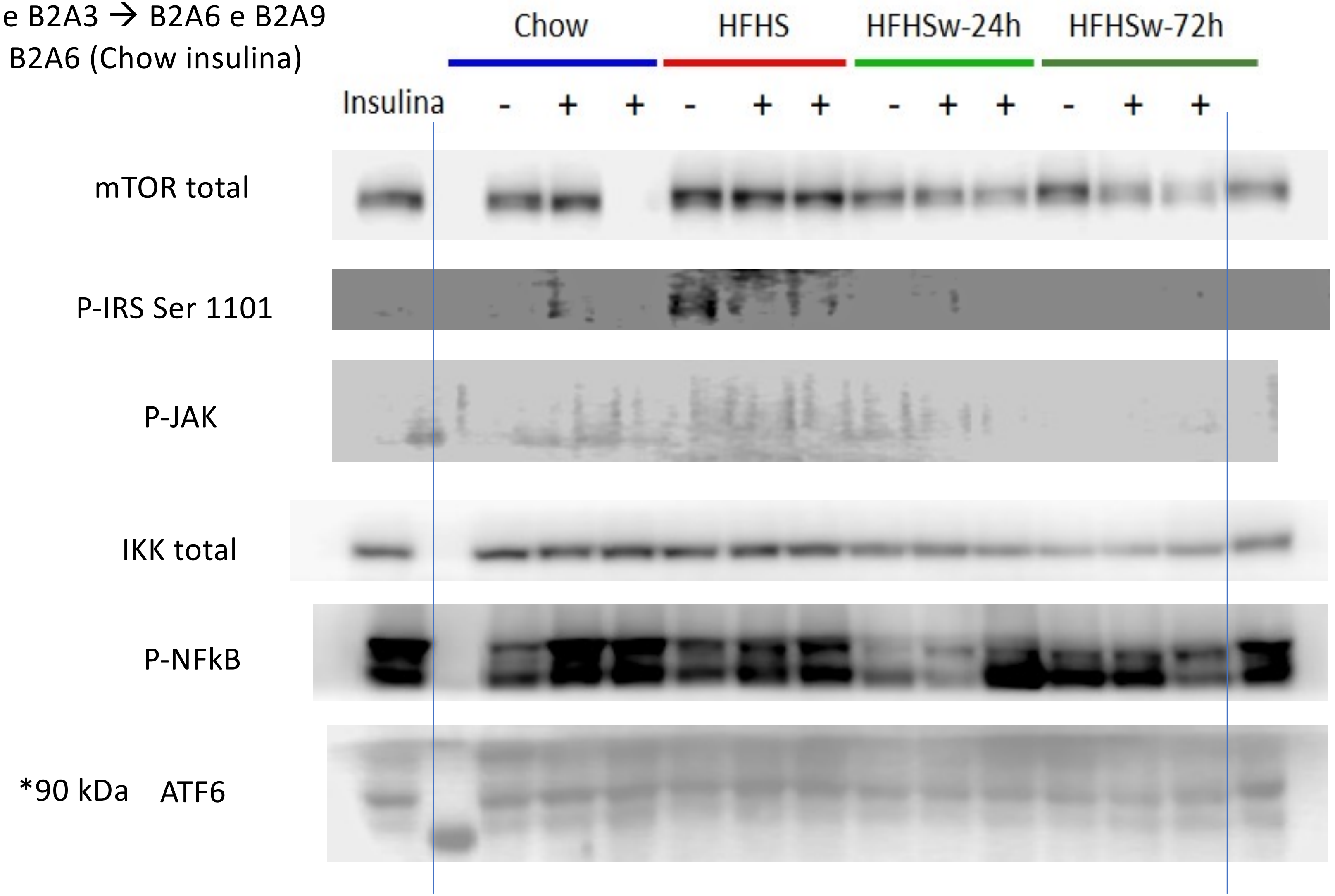

Gel 1 - Stripping

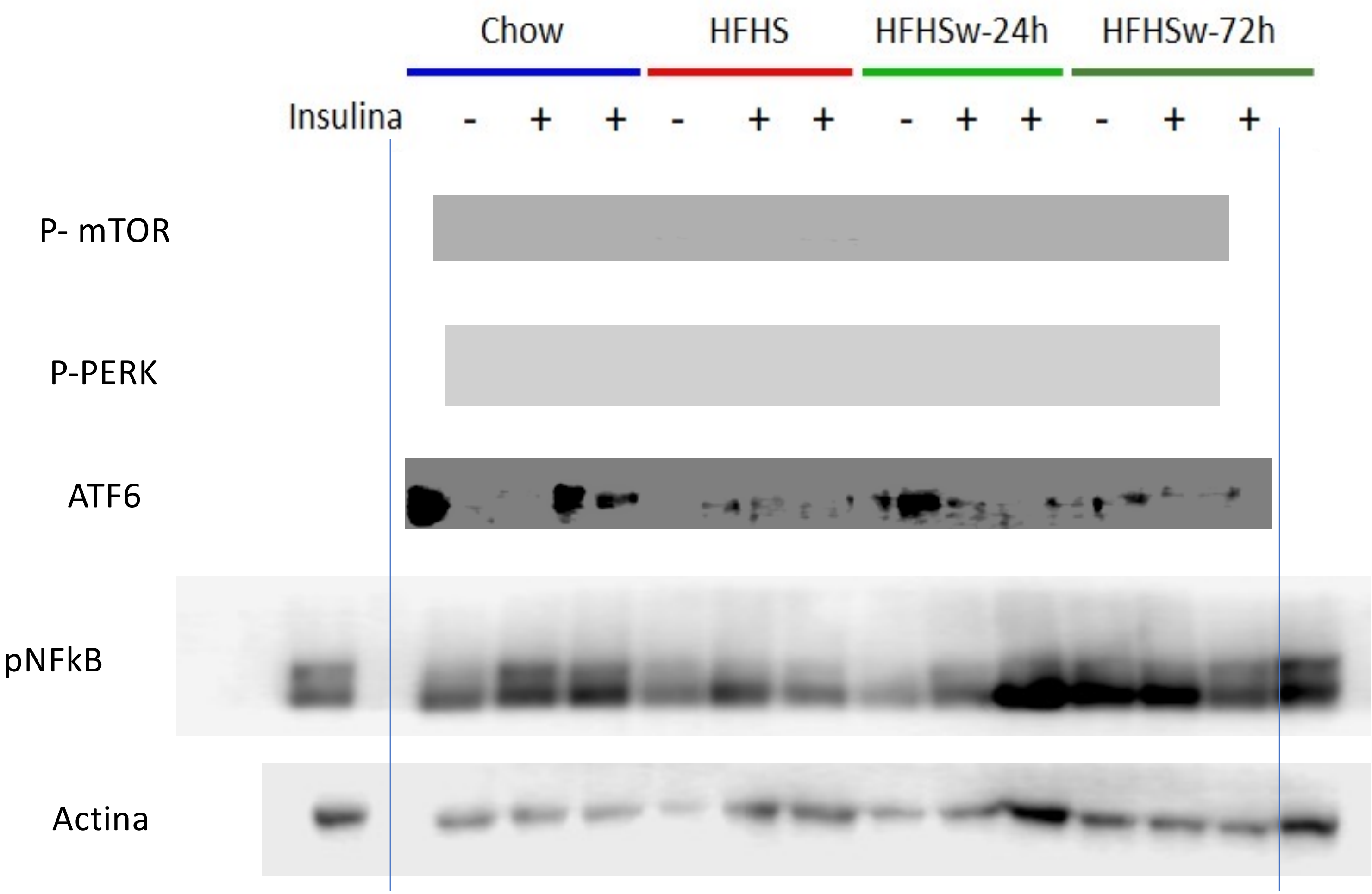

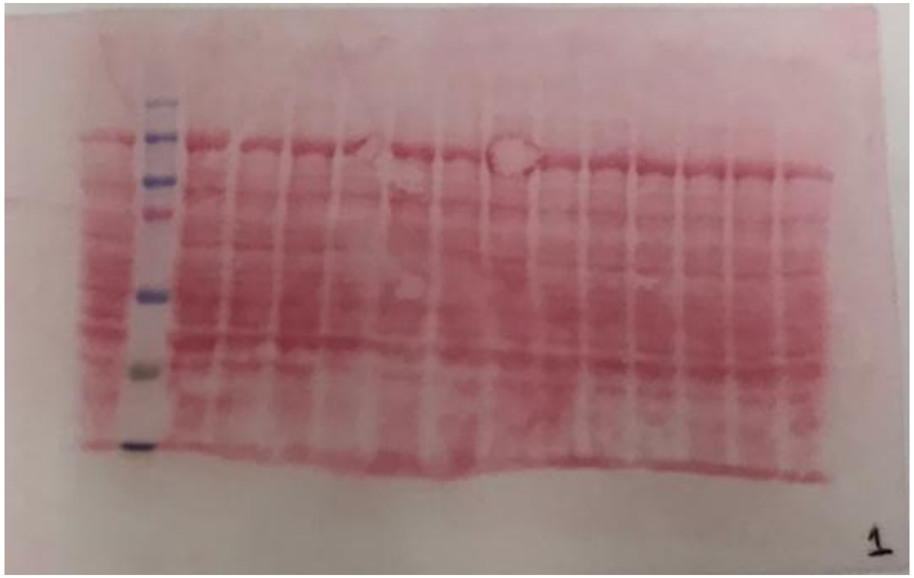
1

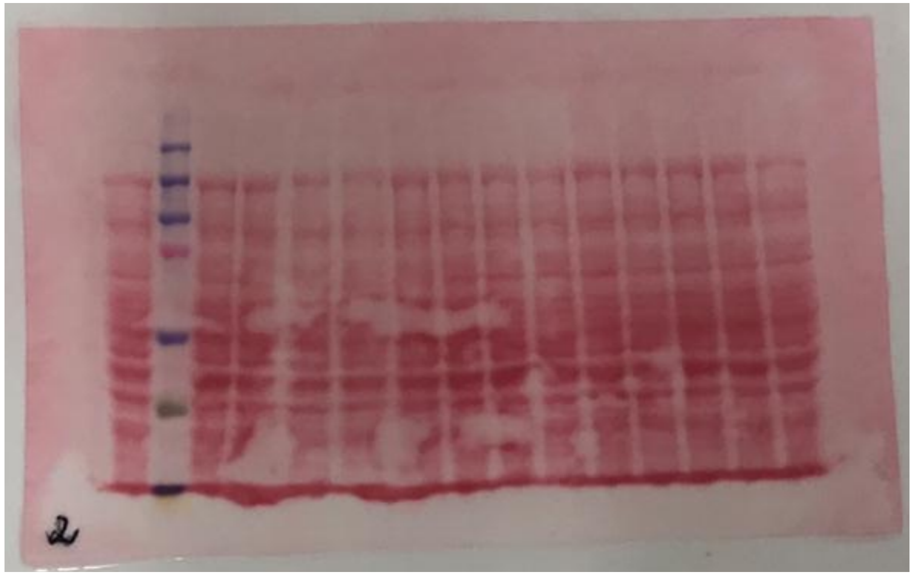
2

Gel 1

- Amostra Chow salina B2A7 → B1A14
- Chow insulina B2A2 e B2A3 → B2A6 e B2A9
- Amostras das pontas: B2A6 (Chow insulina)

B2A9 nunca marcou pmTOR

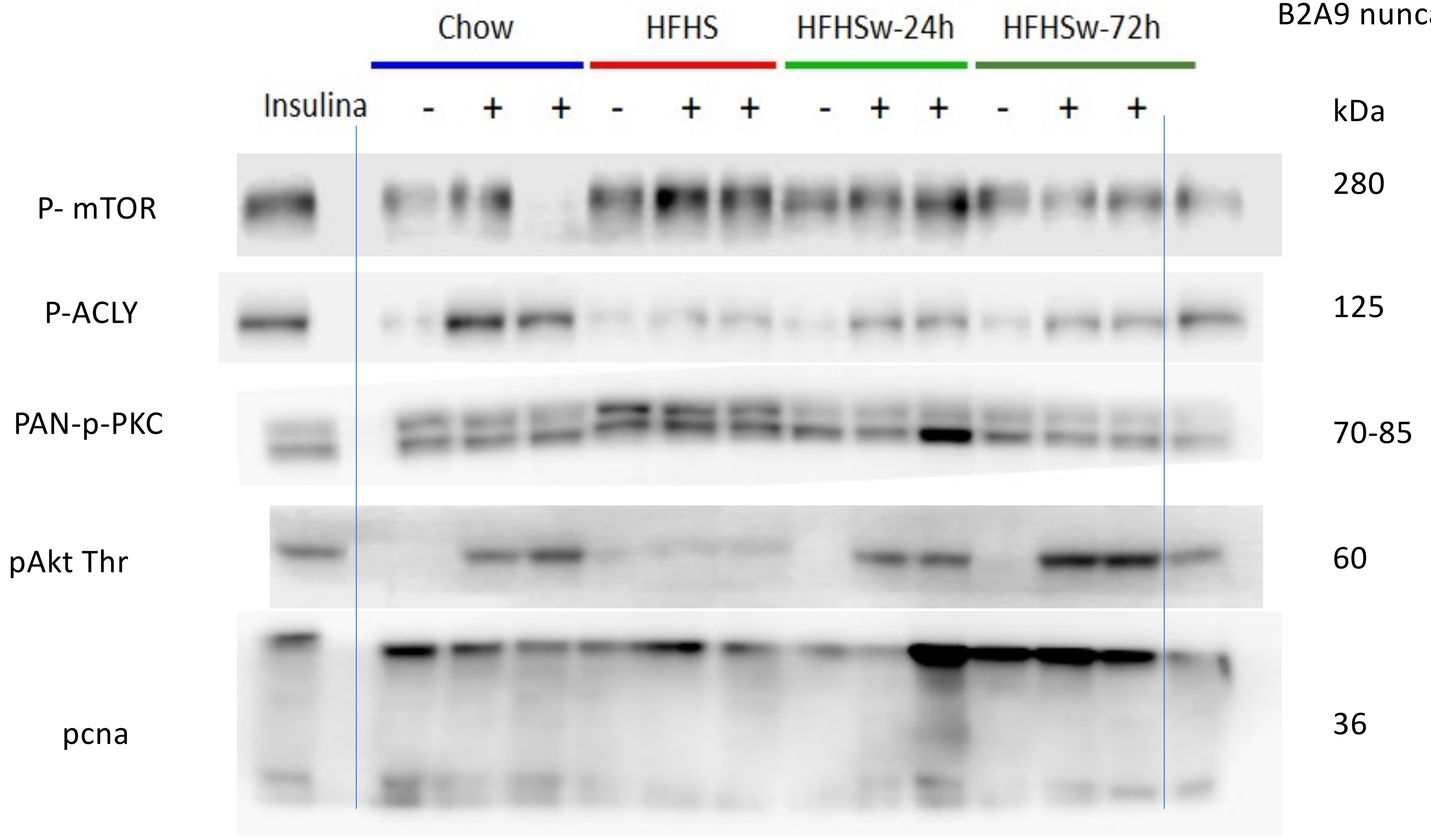

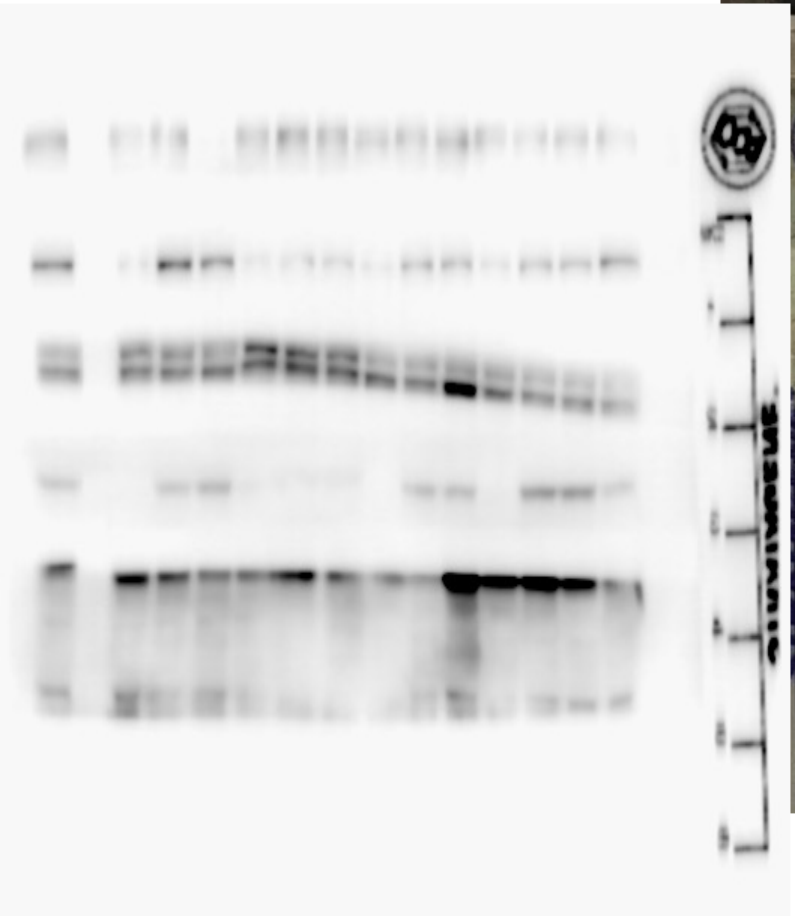

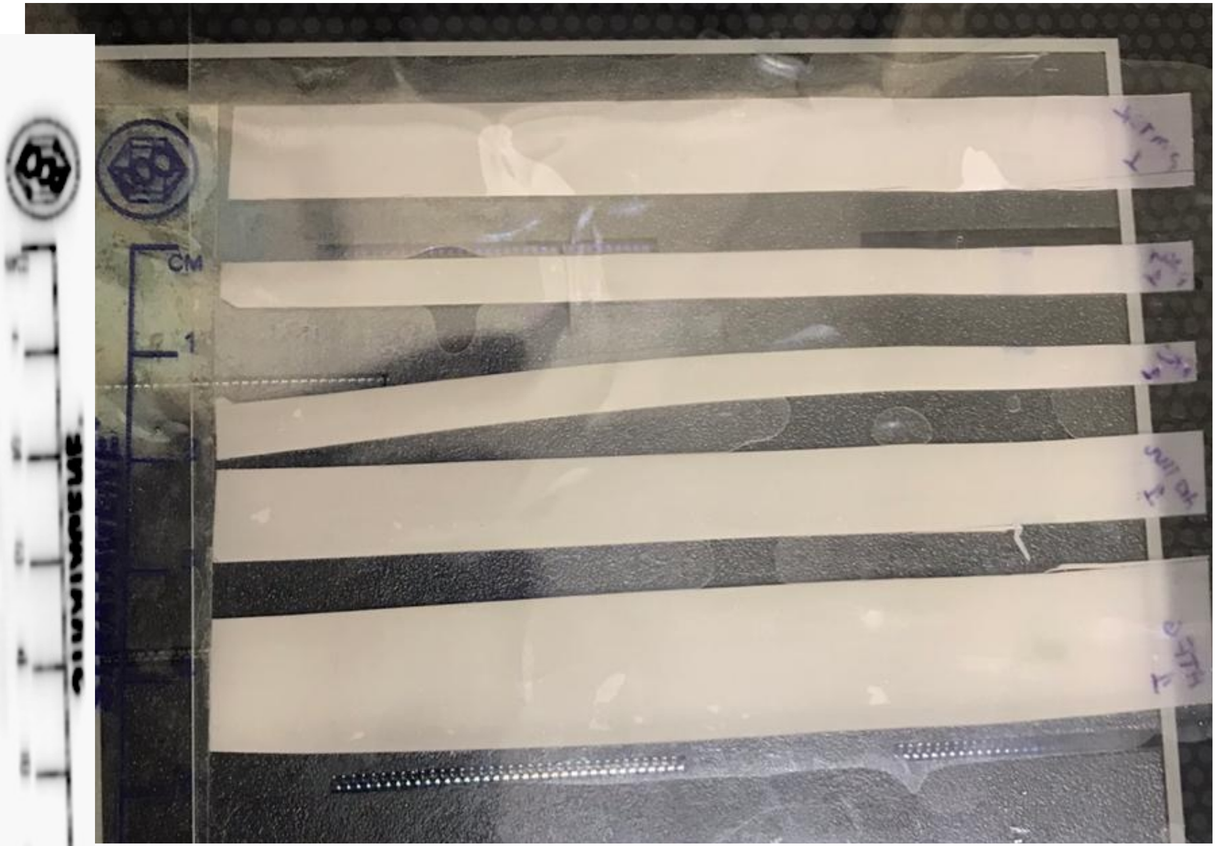

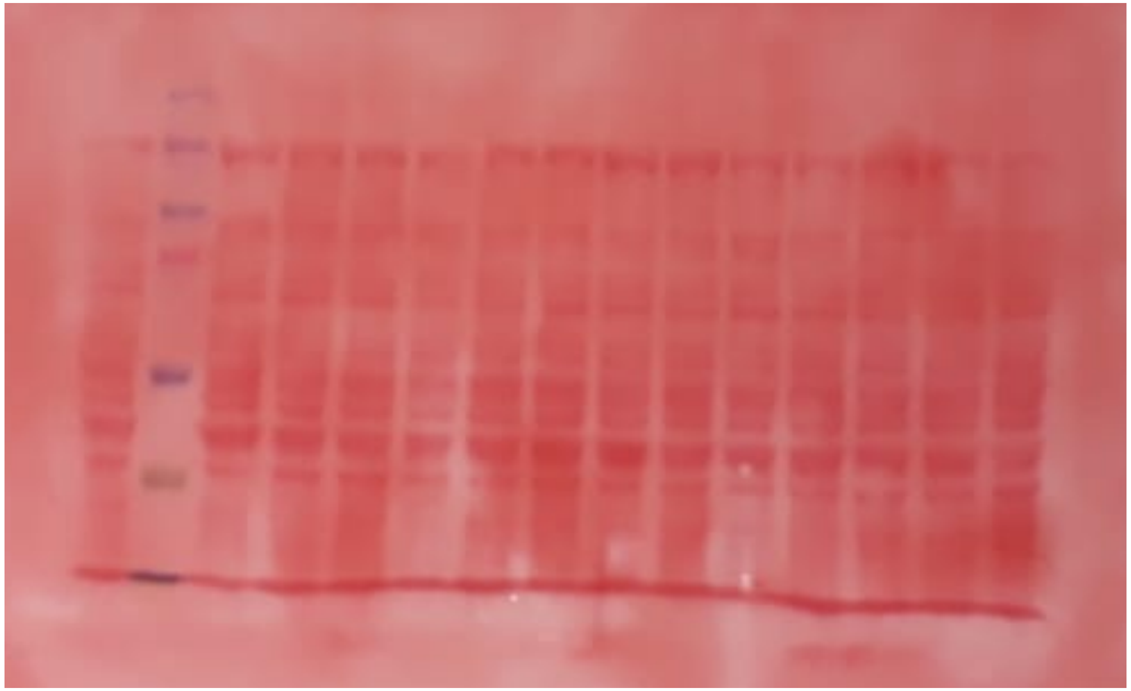

- Amostra Chow salina B2A7 → B1A14
- Amostras das pontas: B2A2 e B2A3 (Chow insulina)

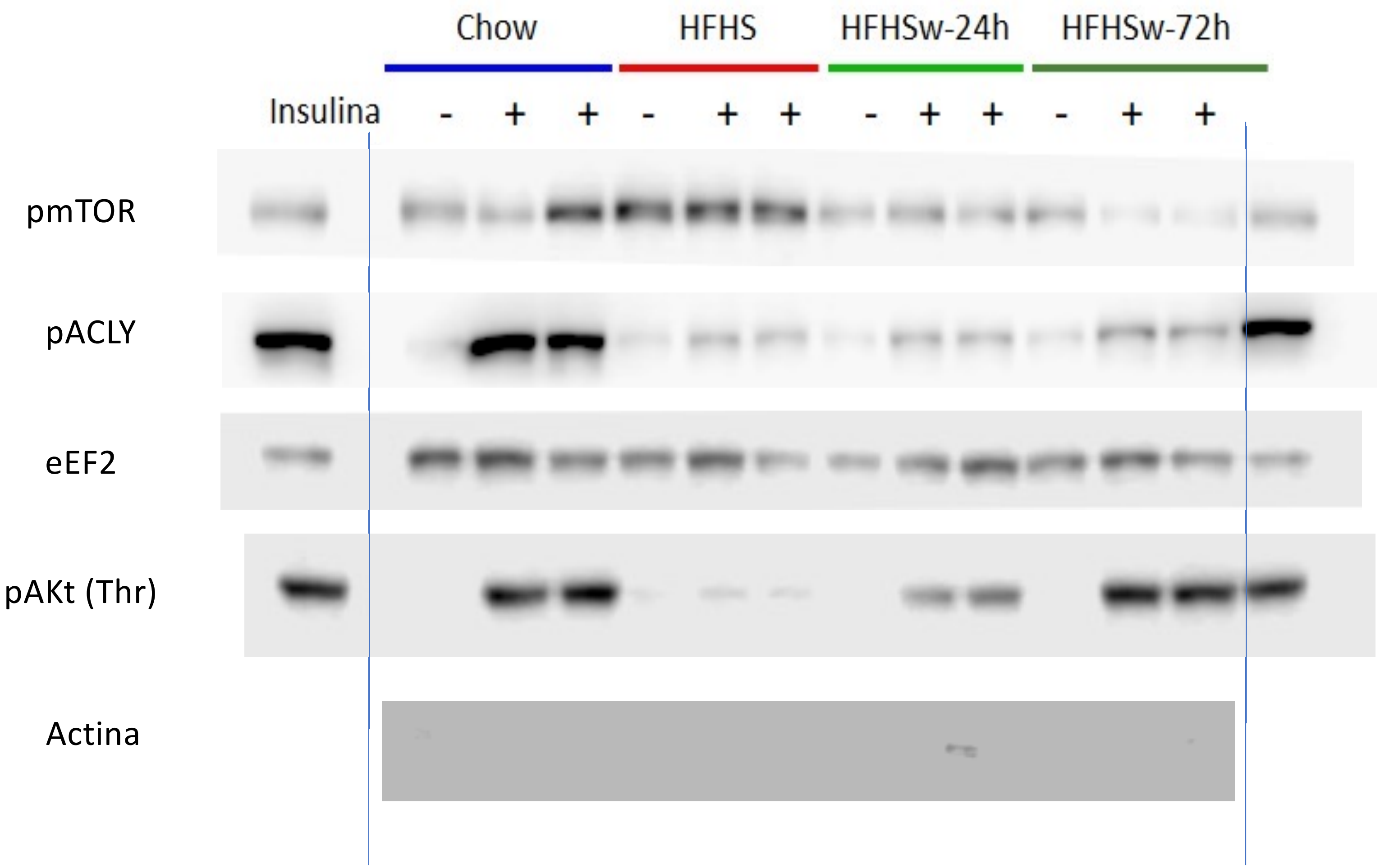

- Amostra Chow salina B2A7 → B1A14
- Amostras das pontas: B2A2 e B2A3 (Chow insulina)

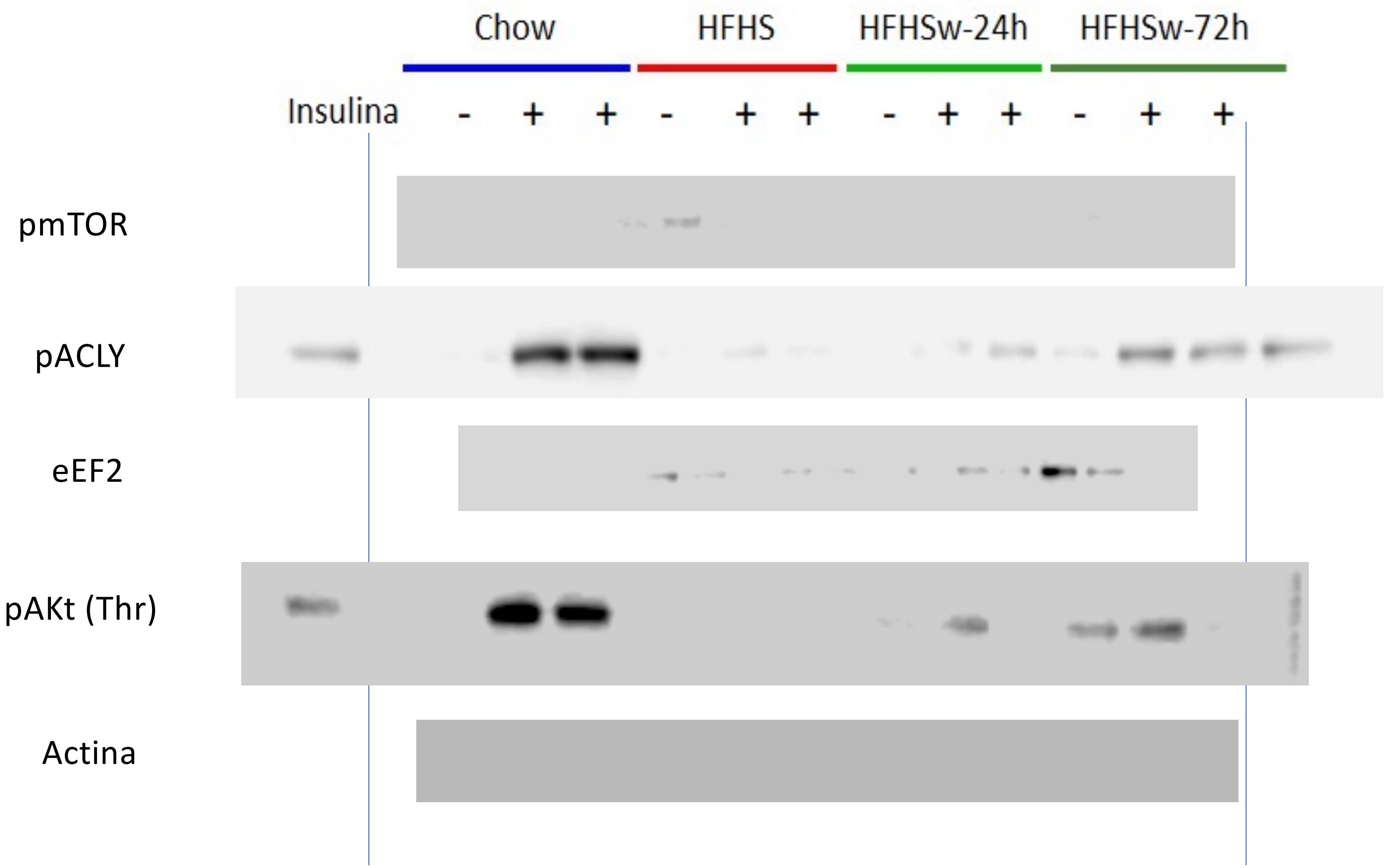

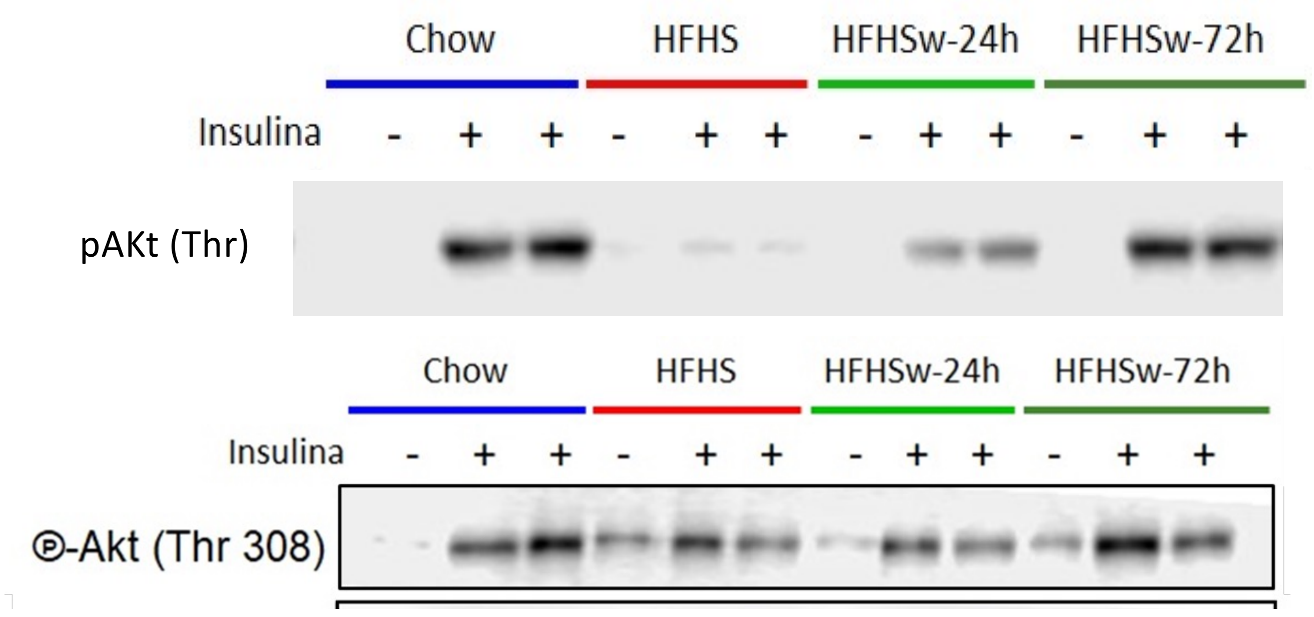
Chow
HFHS
HFHSw-24h
HFHSw-72h
Insulina
- + + - + + - + + - + +
pAKt (Thr)
Chow
HFHS
HFHSw-24h
HFHSw-72h
Insulina
- + + - + + - + + - + +
Ⓟ-Akt (Thr 308)

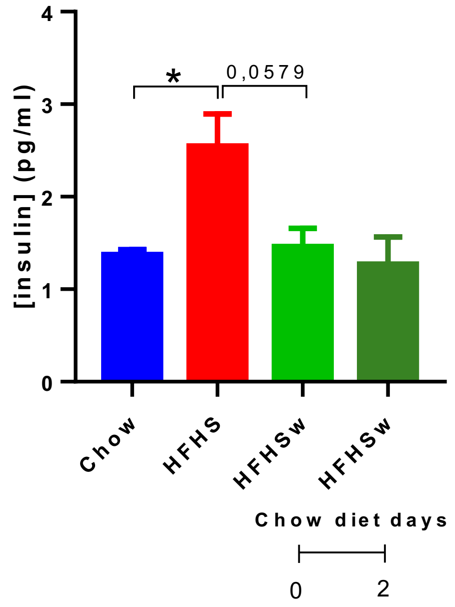
[insulin] (pg/ml)
0
1
2
3
4
*
0,0579
Chow
HFHS
HFHSw
HFHSw
Chow diet days
0
2

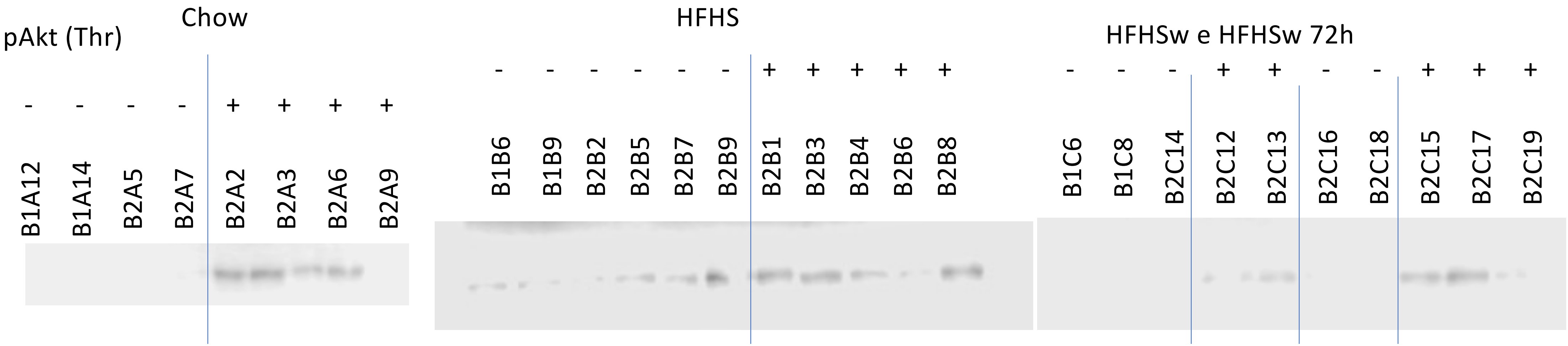
pAkt (Thr)
Chow
- - - - + + + +
B1A12
B1A14
B2A5
B2A7
B2A2
B2A3
B2A6
B2A9
HFHS
- - - - - - + + + + +
B1B6
B1B9
B2B2
B2B5
B2B7
B2B9
B2B1
B2B3
B2B4
B2B6
B2B8
HFHSw e HFHSw 72h
- - - + + - - + + +
B1C6
B1C8
B2C14
B2C12
B2C13
B2C16
B2C18
B2C15
B2C17
B2C19

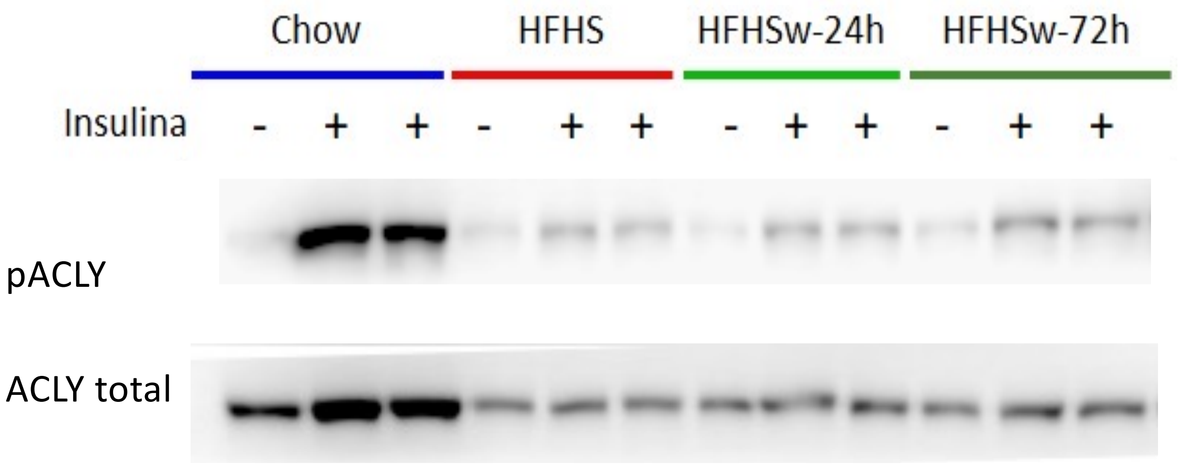
Chow
HFHS
HFHSw-24h
HFHSw-72h
Insulina - + + - + + - + + - + +
pACLY
ACLY total

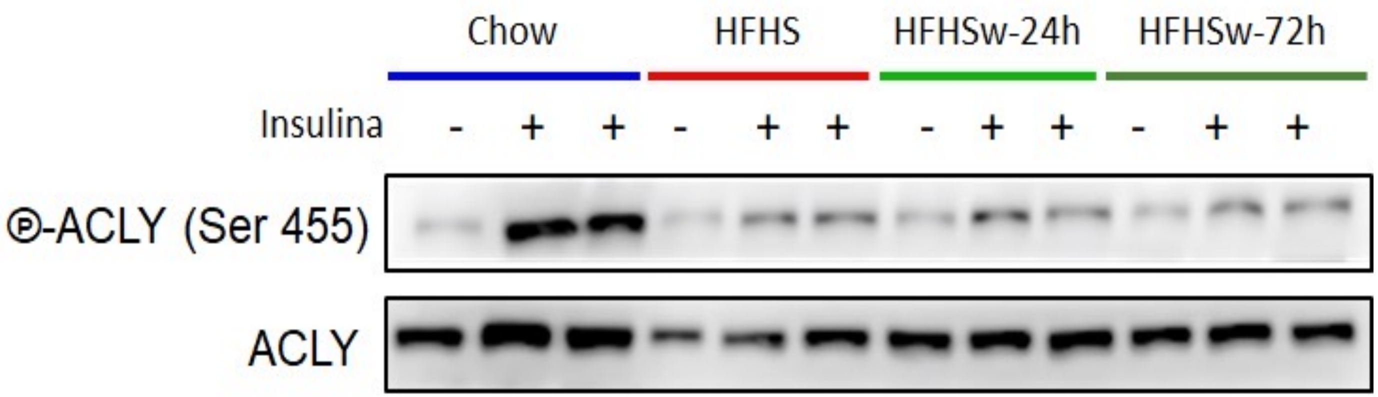
Chow
HFHS
HFHSw-24h
HFHSw-72h
Insulina - + + - + + - + + - + +
Ⓟ-ACLY (Ser 455)
ACLY
